# An Improved Genome-Wide Association Procedure Explores Gene–Allele Constitutions and Evolutionary Drives of Growth Period Traits in the Global Soybean Germplasm Population

**DOI:** 10.3390/ijms24119570

**Published:** 2023-05-31

**Authors:** Can Wang, Xiaoshuai Hao, Xueqin Liu, Yanzhu Su, Yongpeng Pan, Chunmei Zong, Wubin Wang, Guangnan Xing, Jianbo He, Junyi Gai

**Affiliations:** Soybean Research Institute, MARA National Center for Soybean Improvement, MARA Key Laboratory of Biology and Genetic Improvement of Soybean (General), State Key Laboratory for Crop Genetics and Germplasm Enhancement, State Innovation Platform for Integrated Production and Education in Soybean Bio-breeding, Jiangsu Collaborative Innovation Center for Modern Crop Production, Nanjing Agricultural University, Nanjing 210095, China

**Keywords:** soybean (*Glycine max* (L.) Merr.), days of sowing-to-flowering (DSF), days of flowering-to-maturity (DFM), accumulative day-length (ADL), accumulative active temperature (AAT), restricted two-stage multi-locus genome-wide association study based on gene–allele sequence marker (GASM-RTM-GWAS), gene–allele matrix, genetic motivation

## Abstract

In soybeans (*Glycine max* (L.) Merr.), their growth periods, DSF (days of sowing-to-flowering), and DFM (days of flowering-to-maturity) are determined by their required accumulative day-length (ADL) and active temperature (AAT). A sample of 354 soybean varieties from five world eco-regions was tested in four seasons in Nanjing, China. The ADL and AAT of DSF and DFM were calculated from daily day-lengths and temperatures provided by the Nanjing Meteorological Bureau. The improved restricted two-stage multi-locus genome-wide association study using gene–allele sequences as markers (coded GASM-RTM-GWAS) was performed. (i) For DSF and its related ADL_DSF_ and AAT_DSF_, 130–141 genes with 384–406 alleles were explored, and for DFM and its related ADL_DFM_ and AAT_DFM_, 124–135 genes with 362–384 alleles were explored, in a total of six gene–allele systems. DSF shared more ADL and AAT contributions than DFM. (ii) Comparisons between the eco-region gene–allele submatrices indicated that the genetic adaptation from the origin to the geographic sub-regions was characterized by allele emergence (mutation), while genetic expansion from primary maturity group (MG)-sets to early/late MG-sets featured allele exclusion (selection) without allele emergence in addition to inheritance (migration). (iii) Optimal crosses with transgressive segregations in both directions were predicted and recommended for breeding purposes, indicating that allele recombination in soybean is an important evolutionary drive. (iv) Genes of the six traits were mostly trait-specific involved in four categories of 10 groups of biological functions. GASM-RTM-GWAS showed potential in detecting directly causal genes with their alleles, identifying differential trait evolutionary drives, predicting recombination breeding potentials, and revealing population gene networks.

## 1. Introduction

Soybean (*Glycine max* (L.) Merr.) has been cultivated for approximately 5000 years in China [1]. Its planting area has expanded from China as the center of origin to 53° N and 35° S worldwide [2,3]. Originally, soybeans were not suitable for environments at high or low latitudes. As soybeans spread worldwide, they became adapted to the change in environment. The spread was facilitated through four routes, i.e., northward to Northeast China and Russia; eastward to the Korea Peninsular and Japan islands; southward to Southeast Asia, South Asia, Africa, and Australia; and lately westward to northern North America, southern North America, and Central and South America [4].

Soybean is a typical short-day and thermophilic crop. Its growth and development are affected by day-length and temperature [5,6]. Garner and Allard [7] discovered the phenomenon of photoperiodism that regulates the growth period of soybeans. Temperature is another environmental factor affecting the soybean growth period [8]. Therefore, these two factors determine the adaptability of soybean varieties to geographic regions. DSF (days of sowing-to-flowering) and DFM (days of flowering-to-maturity), measured by number of days, do not reflect their relationship with day-length and the temperature of the geographic and climate environments directly. Therefore, the two growth period traits were separated into degree and duration of day-length and temperature, i.e., ADL (accumulative day-length) and AAT (accumulative active temperature), by Wang et al. [9]. The results showed that ADL and AAT for DSF and DFM were markedly different among and within the subgroups of geographic regions and MG-sets (maturity group-sets; worldwide, 3 MG-sets grouped from 13 MGs) [10,11].

For the growth period traits of soybean, 12 major genes, including *E1–E11* and *J*, have been reported [10]. Among them, the potential genes of *E1–E4*, *E9, E10,* and *J* have been isolated. Meanwhile, *FT* genes, *E1La*, *E1Lb*, *PRR3a/3b*, *DT1*, and *DT2*, have been also identified as being related to DSF [12,13,14,15,16,17]. In Arabidopsis, thermo-morphogenesis was found to be controlled by various light signaling pathways, biological clock rhythms [18], plant hormone levels and activities [19,20], and epigenetic mechanism and chromatin level regulation [21,22,23]. However, very few studies regarding these factors in soybeans have been reported. Previous genetic studies on the response to day-length and temperature were mainly carried out for DSF, with little attention being devoted to DFM. As DSF and DFM are measured according to the number of days, which varies with the geographic location at which the materials are tested, the substantial factors that should be tested are their required ADL and AAT. Therefore, the exploration of the gene–allele systems of ADL and AAT required for DSF and DFM is essential to understand the exact relationship between the growth period traits and key environmental factors of ADL and AAT.

Two strategies have been used in genetic study of a target trait; one is to find the individual gene(s) for their functions in a specific material(s), while the other is to explore the complete gene–allele system of the trait in a natural population. Plant breeders are especially interested in finding and converging all of the superior alleles on different loci that exist in historical germplasm populations for cultivar development. Genome-wide association study (GWAS) has been widely used for the genetic dissection of complex traits in germplasm populations. However, previous GWAS procedures concentrated on finding a few major loci, such as the general linear model (GLM) and mixed linear model (MLM) approaches [24,25,26], based on single-locus models, and even the multi-locus mixed-model (MLMM) [27] and multi-locus random-SNP-effect mixed linear model (mrMLM) [28]. Breeders are likely more interested in exploring the whole quantitative trait locus (QTL)–allele constitution in a population for an optimal genetic functioning. He et al. [29,30] proposed the innovative restricted two-stage multi-locus GWAS (RTM-GWAS) procedure.

RTM-GWAS is characterized by two innovations. First, using the genomic single nucleotide polymorphism linkage disequilibrium block (SNPLDB) as markers to meet the requirement of multiple alleles per locus in a germplasm population, and using the genetic similarity coefficient matrix of whole-genome SNPLDBs to correct the population structure bias for reducing false positives and negatives. Second, using the multi-locus model to identify the complete QTL–allele set to avoid false positives inflated by neighboring loci and false negatives caused by stringent significance correction for the single-locus model, as well as using two-stage analysis with trait heritability as the upper limit to control the calculation of noise and false positives based on incorporated precise experimental design. In this way, the significance level of *p* = 0.05 under the multi-locus model is equivalent to Bonferroni criterion of 0.05/m (m is the number of markers) under the single-locus model. The RTM-GWAS procedure has been applied to identify QTL–allele systems for growth period, seed quality, resistance to insects, and tolerance to environmental stress traits in soybeans for germplasm populations and bi-parental populations. The identified QTL–alleles were further used for gene annotation, optimal cross-design, and evolution mechanism studies [31,32,33,34,35,36].

Liu et al. [10] used RTM-GWAS to identify 52 QTLs for days of sowing-to-flowering and 59 QTLs for days of sowing-to-maturity in the global soybean population. From the QTLs, 44 and 36 candidate genes were annotated, respectively, and grouped into a similar set of 10 functional groups. A previous study (Su et al., 2023, accepted by Theoretical and Applied Genetics) suggested using gene–allele sequence as genomic markers (GASM) to replace SNPLDB in direct detection of the gene–allele system to avoid the inference from QTLs to genes based on whole genome sequencing. The present study follows this research strategy to use GASM-RTRM-GWAS to identify direct gene–allele systems of DSF and DFM and their required ADL and AAT.

Because RTM-GWAS can identify a relatively complete set of QTL–alleles, Liu et al. [37] explored the evolutionary mechanism from later to earlier in the annual wild and cultivated soybean in China through comparisons between the QTL–allele matrices of geographic subpopulations. This also made it possible to explore the gene network of a trait in a population using Gene Ontology (GO) analysis (http://www.soybase.org (accessed on 1 January 2021)). The protein–protein interaction (PPI) network is a procedure for analyzing how proteins work together in cells to perform cellular functions in a coordinated manner [38,39,40]. PPI calculation and prediction have been applied in plants such as Arabidopsis [41] and rice [42].

The present study aimed to identify the whole-genome gene–allele constitution of DSF and DFM and their required ADL and AAT using GASM-RTM-GWAS. This was used to explore the genetic mechanism in geographic adaptation and MG-set expansion, to predict the genetic recombination potential of the traits, and to explore their gene–allele networks and identify key gene–alleles to improve the traits in the global soybean population. This study is characterized by two features: first, directly identifying the gene–allele systems of growth periods and their response to day-length and temperature through the innovative GASM-RTM-GWAS; and second, targeting the recombination potentials and evolutionary motivators for further world-wide extension of soybeans based on the complete gene–allele information of the growth periods and related eco-traits.

## 2. Results

### 2.1. Identification of Gene–Allele Systems of DSF and DFM in Global Soybeans

**Figure 1:**
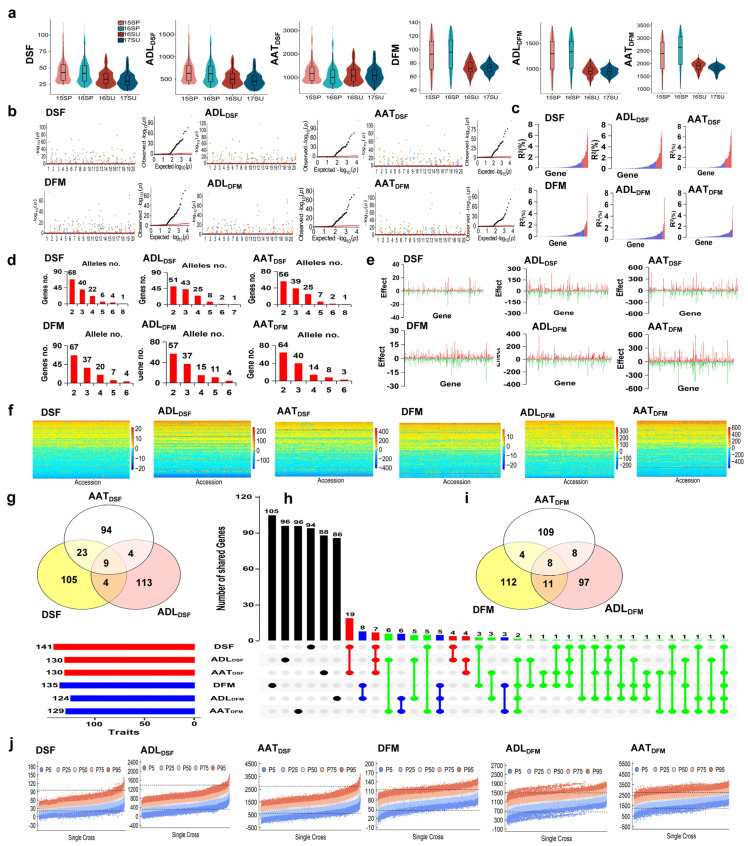
Identification and recombination potential of the gene–allele systems of six DSF (days of sowing-to-flowering)- and DFM (days of flowering-to-maturity)-related traits in the world soybean germplasm population (WSGP). (**a**) The violin diagram of the six DSF- and DFM-related traits in the WSGP. (**b**) Manhattan (**left**) and Q–Q plots (**right**) of the six DSF- and DFM-related traits. (**c**) Phenotypic contribution of genes detected for the six DSF- and DFM-related traits. The vertical axis indicates the genetic contribution of a gene, while the horizontal axis is the genes with red color as *R*^2^ ≥ 1.5% and blue color as *R*^2^ < 1.5%. (**d**) The allele number distribution of genes for the six DSF- and DFM-related traits. (**e**) Distribution of allele effect values of genes for the six DSF- and DFM-related traits. (**f**) The gene–allele matrix of the six DSF- and DFM-related traits. (**g**) Venn diagram of genes conferring the three DSF-related traits. (**h**) Venn diagram of genes conferring the six DSF- and DFM-related traits (note: Red represents shared genes between DSF- related traits; blue represents shared bases between DFM- related traits; green represents shared genes between six DSF- and DFM- related traits). (**i**) Venn diagram of genes conferring the three DFM-related traits. (**j**) The recombination potential prediction of the six DSF- and DFM-related traits in the WSGP based on the linkage model.

DSF and DFM were related to the degree and duration of two basic environment factors, ADL and AAT, respectively. From the center of origin to extended geographic regions, the required ADL and AAT for DSF and DFM changed greatly to adapt to the local geographic and sowing seasonal conditions [9] (Table S1, Figure 1a).

Under the G × E model of GASM-RTM-GWAS, 141 and 135 genes with 406 (2–8 or 2.88/gene) and 386 (2–8 or 2.84/gene) alleles were identified on the 20 chromosomes for DSF and DFM, explaining 76.85% and 55.03% of phenotypic variance (PV), respectively, ranging from 0.00 to 6.95%/gene and 0.00 to 7.20%/gene, respectively. There were 124 and 127 genes with *R*^2^ < 1.5%, explaining 25.22% and 32.63% PV, respectively, and 17 and 8 genes with *R*^2^ ≥ 1.5%, explaining 51.63% and 22.40% PV, respectively; therefore, 18,85% and 25.67% of the genetic variation (*h^2^*−PV_gene_ = 95.70−76.85% = 18.85% and 80.70−55.03% = 25.67%, respectively) were due to a collective of minor genes. The G × E variance contributed 4.20% and 18.80% to the PV for DSF and DFM, respectively, which is relatively low (Table 1, Figure 1b,c). Only seven genes were shared, indicating different genetic systems between the two traits. Comparing the identified QTLs reported by Liu et al. (2021) using a similar population as in the present study, 141 genes vs. 52 QTLs (44 annotated genes with 39 consistent in this study) for DSF and 135 genes vs. 59 QTLs (36 annotated genes with 34 consistent in this study) for DFM were observed. These results indicated that more DSF and DFM genes were identified using GASM-RTM-GWAS than using SNPLDB-RTM-GWAS. In addition, compared with SoyBase (http://www.soybase.org (accessed on 1 January 2021)), 63 (44 loci) and 28 (23 loci) QTLs/genes are similar to the DSF and DFM genes discovered in this study (Tables S2 and S3), while the other 97 and 110 genes explaining 52.45% and 38.14% of the present study PV, respectively, were newly detected. Accordingly, GASM-RTM-GWAS is powerful and effective in identifying gene–allele systems of complex quantitative traits and performed better than SNPLDB-RTM-GWAS. This is due to the GASM markers being more relevant than the SNPLDB markers, meaning that the haplotype number of a GASM fits the allele number of the corresponding causal gene better because GASM targets the same segment of the causal gene in the population.

### 2.2. Identification of Gene–Allele Systems of ADL and AAT Required for DSF and DFM in Global Soybeans

The identified gene–allele results from the GASM-RTM-GWAS G × E model for ADL_DSF_, AAT_DSF_, ADL_DFM_, and AAT_DFM_ are listed in Tables S4–S7, with their summarized results in Table 1 and Table S8. A total of 459 genes were detected for the four traits.

For ADL_DSF_, 130 genes were detected with 390 alleles (2–8 or 3.00/gene), explaining 75.35% PV, ranging from 0.00% to 6.77% per gene. There were 116 genes with *R*^2^ < 1.5%, explaining 30.03% PV, and 14 genes with *R*^2^ ≥ 1.5%, explaining 45.32% PV. In addition, 20.45% of the genetic variation (95.80−75.35% = 20.45%, Table 1) was due to a collective of minor genes. For AAT_DSF_, 130 genes were detected, with 384 alleles (2–6 or 2.95/gene), explaining 73.76% PV, ranging from 0.00% to 7.32% per gene. There were 111 genes with *R*^2^ < 1.5%, explaining 15.52% PV, and 19 genes with *R*^2^ ≥ 1.5%, explaining 58.24% PV. In addition, 25.44% of the genetic variation (99.20−73.76% = 25.44%) was due to a collective of minor genes. The G × E variance accounted for only 3.93% and 0.55% PV for ADL_DSF_ and AAT_DSF_, respectively (Table 1, Figure 1b,c). Comparing the previously reported DSF genes (QTLs) to the present ADL_DSF_ and AAT_DSF_ genes, assuming that DSF and ADL_DSF_ and AAT_DSF_ are interrelated, 67 (44 loci) and 65 (47 loci) reported genes (QTLs), respectively, were close to the DSF genes discovered in this study. Therefore, at least 86 other ADL_DSF_ and 83 other AAT_DSF_ genes were not related to DSF previously, but were newly detected, which explains 51.61% and 51.76% of the present total ADL and AAT PV, respectively (Table 1, Tables S4 and S5).

For ADL_DFM_, 124 genes were detected, with 364 alleles (2–6 or 2.94/gene), explaining 50.24% PV, ranging from 0.00 to 7.18% per gene. There were 119 genes with *R*^2^ < 1.5%, explaining 33.95% PV, and 5 genes with *R*^2^ ≥ 1.5%, explaining 16.29% PV. In addition, 28.26% PV (78.50−50.24% = 28.26%) was due to a collective of minor genes. For AAT_DFM_, 129 genes with 362 alleles (2–6 or 2.81/gene) were detected, explaining 41.54% PV, with each gene ranging from 0.00 to 5.71%. There were 126 genes with *R*^2^ < 1.5%, explaining 31.20% PV, and 3 genes with *R*^2^ ≥ 1.5%, explaining 10.34% PV. In addition, 29.36% PV (70.90−41.54% = 29.36%) was due to a collective of minor genes. The G ×E variance accounted for 20.90% and 29.00% PV for ADL_DFM_ and AAT_DFM_, respectively, which is larger than those for DSF (Table 1, Figure 1b,c). Comparing the previously reported DFM genes (QTLs) to the present ADL_DFM_ and AAT_DFM_ genes, assuming DFM and ADL_DFM_ and AAT_DFM_ were interrelated, 33 (23 loci) and 28 (22 loci) previously reported genes (QTLs), respectively, were close to the DFM genes discovered in this study. Therefore, at least 101 ADL_DFM_ and 107 AAT_DFM_ other genes were not related to DFM, but newly detected in the present study, which explains 33.58% and 29.49% of the present total PV, respectively (Table 1, Tables S6 and S7).

The above results indicated that the gene–allele systems of ADL_DSF_, AAT_DSF_, ADL_DFM_, and AAT_DFM_ were different from each other, whereas those of ADL_DSF_ and AAT_DSF_ were closely related to DSF and those of ADL_DFM_ and AAT_DFM_ were less closely related to DFM. Table 2 shows that there were 13 shared genes between ADL_DSF_ and AAT_DSF_, which explained 32.82% and 38.19% of their PV, respectively, indicating that both traits are genetically related and may form the genetic basis for ADL × AAT, causing the DSF variation. There were 16 shared genes between ADL_DFM_ and AAT_DFM_, which explained only 15.59% and 10.44% of their PV, respectively, and were less closely interrelated at the DFM stage.

The identified genes with their alleles for each trait were arranged in a gene–allele matrix, which consisted of all gene–allele information of the 354 soybean varieties in the global population, including alleles with positive and negative effects (Figure 1d–f).

### 2.3. Differential Contributions of ADL and AAT to DSF and DFM in Global Soybeans

Table 2 summarizes the shared genes among DSF, ADL_DSF_, and AAT_DSF_, as well as among DFM, ADL_DFM_, and AAT_DFM_. Nine genes were common among DSF, ADL_DSF_, and AAT_DSF_, which explained 35.09%, 32.60%, and 37.89% PV, respectively, indicating that ADL and AAT are a large part of the common genetic basis for DSF. In addition, DSF and AAT_DSF_ shared 23 other genes (24.16%), while DSF and ADL_DSF_ shared only 4 other genes (0.38%). This indicates that ADL and AAT are both important to DSF, but AAT has a greater impact on DSF variation (59.25%) than ADL (35.47%). In addition, DSF, ADL_DSF_, and AAT_DSF_ had 94, 96, and 88 trait-unique genes, respectively, with only 14.75%, 31.65%, and 10.49% contributions to their respective PVs. These trait-unique genes were scattered among the four functional categories of 10 groups (Table 3), indicating that different genes with similar functions were trait-unique for different traits. For example, seven genes related to light and circadian rhythm were present in DSF, six of which were unique, while in ADL_DSF_, eight genes related to light and circadian rhythm were present, of which six were unique genes (Figure 1g,h).

In DFM, ADL_DFM_, and AAT_DFM_, eight common genes were found, which explained 11.54%, 11.33%, and 8.63% of their respective PVs. DFM and ADL_DFM_ shared 11 genes (12.77%) and DFM and AAT_DFM_ shared four genes (2.39%). This indicates that ADL_DFM_ (24.31%) contributed more genetically than AAT_DFM_ (13.93%) to DFM, but it was less when compared with that in DSF; therefore, DFM was less genetically influenced by ADL and AAT than DSF. In addition, in DFM, ADL_DFM_, and AAT_DFM_, 105, 86, and 96 trait-unique genes, respectively, explained 26.59%, 18.43%, and 28.33% of their respective PVs. These trait-unique genes were scattered among the four functional categories of 10 groups (Table 4), indicating that different genes with similar functions were unique for the three traits (Figure 1h,i).

Therefore, DSF shared more ADL and AAT contributions than DFM, or in other words, the three DFM-related gene systems involving the DFM traits are more independent from each other than those of the DSF-related traits. Furthermore, DSF shared more genes and PVs with AAT_DSF_ than ADL_DSF_, while DFM shared more genes and PVs with ADL_DFM_ than AAT_DFM_, indicating that AAT was more important in determining DSF and ADL was more important in determining DFM in global soybeans. The relative importance of ADL_DFM_ to DFM length is a new concept that coincided with the report of Han and Gai [49].

In addition, the three DSF-related traits among the nine shared genes, *Glyma06g23580*, *Glyma10g26450,* and *Glyma16g03320*, are close to the confirmed *E1, E4*, and *LHY1a*, respectively, while *Glyma01g22830*, *Glyma02g04190*, *Glyma08g03210*, *Glyma13g07110*, *Glyma17g08460,* and *Glyma17g09500* have not been reported in previous studies. There were 94, 96, and 88 trait-specific genes in DSF, ADL_DSF_, and AAT_DSF_, but they only explained 14.75%, 31.65%, and 10.49% of their respective PVs, thus these specific genes provided small contributions to their PV. For the three DFM-related traits, among the eight shared genes, *Glyma19g34740* is close to the *Dt1/TFL1b* gene, while *Glyma02g00371*, *Glyma07g40260*, *Glyma08g15870*, *Glyma12g06580*, *Glyma12g06950*, *Glyma13g09470*, and *Glyma13g25480* have not been reported in previous studies. There were 105, 86, and 96 trait-specific genes in DFM, ADL_DFM_, and AAT_DFM_, which explained 26.59%, 18.43%, and 28.33% of their respective PVs, that is, a little more than the contributions of DSF-related traits (Table 2).

### 2.4. Differentiated Evolutionary Motivators in Geographic Adaptation and MG Expansion for Growth Period Traits of Global Soybeans

Based on the relatively thorough identification of genome-wide genes–alleles, the dynamic allele changes due to the different motivators were calculated from the five geographic submatrices (O, A, B, C, and D) and three MG-set submatrices (MG I-VII or P MG-set, MG 0-000 or E MG-set, and MG VIII-X or L MG-set) for the six traits. Here, the designations of the geographic and MG-set submatrices are listed in Table 4.

In adaptation to geographic regions, the allele changes showed a similar tendency between DSF, ADL_DSF_, and AAT_DSF_ and DFM, ADL_DFM_, and AAT_DFM_. In the three DSF-related traits, there were 340–364 alleles in the center of origin O. From O to A, B, C, and D, a dominant share of the alleles, 307–336, were passed down; a small share of the alleles (14–35, less than 10%) were excluded; and a number of new alleles (23–39, less than 11%) emerged. Altogether, the emerged alleles in ABCD (i.e., A+B+C+D) were added to the 42–50 ones with 16–23 negative effect ones and 17–32 positive effect ones, and the excluded alleles in ABCD were added to 0–1 alleles with 0–1 positive ones and 0–1 negative ones. In the three DFM-related traits, there were 320–340 alleles in the center of origin O. From O to A, B, C, and D, a dominant share of the alleles, 292–316, were passed down; a small share of the alleles (18–31, less than 10%) were excluded; and a number of new alleles (21–36, less than 11%) emerged. Altogether, the emerged alleles in ABCD were added to 41–44 ones, with 21–22 negative effect ones and 20–22 positive effect ones, and the excluded alleles in ABCD were added to 0 with 0 positive ones and 0 negative ones (Figure S1a,c).

In the MG-set expansion, the allele changes showed a similar tendency for DSF- and DFM-related traits, but were different from those in geographic adaptation. In the three DSF-related traits, there were 383–405 alleles in the primary MG-set. From the P MG-set to the E and L MG-sets, a dominant share of the alleles, 275–352, were passed down; a share of the alleles (47–109, about 12–27%) were excluded; and a few new alleles (0–1, approximately 0%) emerged. Altogether, the emerged alleles in E and L MG-sets were added to 1 allele with a positive effect, and the excluded alleles in E and L MG-sets were added to 21–30 alleles with 10–15 negative ones and 9–20 positive ones. In the three DFM-related traits, there were 362–384 alleles in the P MG-set. From the P MG-set to the E and L MG-sets, a dominant share of the alleles, 274–332, were passed down; a share of the alleles (52–92, about 13–24%) were excluded; and no new alleles emerged. Altogether, the emerged alleles in E and L MG-sets were added to 0, and the excluded alleles in E and L MG-sets were added to 23–41 with 9–15 negative ones and 10–26 positive ones (Figure S1b,d).

The trends of the evolutionary gene–allele changes in DSF and DFM due to geographic region adaptation and MG-set expansion in the present study were consistent with ADL_DSF_ and AAT_DSF_ and with ADL_DFM_ and AAT_DFM_. In both the geographic adaptation and MG-set expansion, allele inheritance (or migration) was always the dominant part. However, in geographic adaptation, new allele emergence (or mutation) was a joint major dynamic motivator for individual adapted sub-regions and total sub-regions, while allele exclusion (or selection) appeared in individually adapted sub-regions, but not in the total adapted sub-regions, even almost without allele exclusion (only one allele). Meanwhile, in the MG-set expansion, allele exclusion was a major dynamic motivator, even without new allele emergence, in all individual and total expanded MG-sets. Therefore, in the two evolutionary processes, new allele emergence was an active motivator for geographic adaptation, while allele exclusion was an active motivator for MG expansion. This is true for all six traits. In addition to allele inheritance, emergence, and exclusion, allele recombination based on the allele changes should also be an important and constant dynamic motivator, which is shown in the next section.

In both allele emergence and exclusion, the changed alleles might have positive or negative effects, which indicate that the actual mutation and selection were not unidirectional. Both negative and positive alleles were emerged or excluded regardless of geographic adaptation and MG-set expansion, but the final comprehensive results were consistent with the target of the breeding effort.

### 2.5. Genetic Recombination Potential of DSF and DFM and Their Required ADL and AAT in Global Soybeans

To evaluate the genetic recombination potential of the six traits, the 25th percentile and 95th percentile progenies in each of the 62,128 possible crosses (parents’ pairs) among the 354 varieties were predicted based on linkage and independent assortment models. The linkage model means that the original linkages among the GASMs are reserved, while the independent assortment model means that the original linkages among GASMs are not considered. The predicted results (Table 5) showed a similar outcome for the six traits under the linkage model, in which a wide range of the predicted 25th and 95th percentile progenies, distributed among the 62,128 parental pairs, were much lower and higher than the extremes of the 354 varieties, respectively. These showed lower and higher parent transgressive segregations in 992–6352 crosses for 25th percentile segregation and 1631–27,142 crosses for 95th percentile segregation in the six traits (Table 5, Figure 1j). Interestingly, for the six traits in the global population, the predicted results under the independent assortment model were similar to those under the linkage model. This indicates that no extra increment can be expected from further elimination of the linkage drags.

Considering the genetic structure of the accessions of the global population (Figure 1f), few accessions had their gene–allele structure composed of all alleles with the lowest (or highest) effects or all accessions in a complementary mode. As there were 124–141 genes identified for each of the six traits, the recombination potential among the large number of gene–alleles should be sufficiently rich, even more than the actual allele emergence or exclusion. This explains why significant progress has been made during the recent couple of centuries while the mutation rate has usually only been at the 10^−6^ level.

### 2.6. Gene Functions of DSF and DFM and Their Required ADL and AAT in Global Soybeans

**Figure 2:**
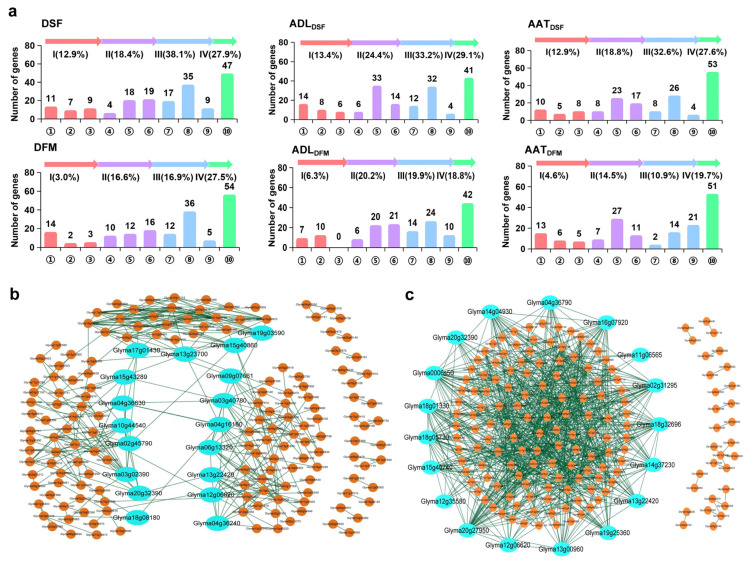
Gene functional classifications and protein–protein interaction networks of the six DSF- and DFM-related traits in the WSGP. (**a**) Gene ontology classifications of the six DSF- and DFM-related traits in the WSGP. The four gene ontology categories with their groups are as follows: Category I: Genes related to flowering, seed and stem development, or response to light and temperature stimulation, including Group ①, genes related to flower development and growth; Group ②, genes related to light and circadian rhythm; and Group ③, genes related to temperature response. Category II: Translocation signal transduction; defense response; and genes related to DNA methylation, transcription, RNA processing, and chromosome modification, including Group ④, genes related to histone variants and chromosome modification; Group ⑤, genes related to DNA methylation, transcription and RNA processing; and Group ⑥, genes related to signal transduction and transport. Category III: Primary metabolism genes related to secondary metabolism, including Group ⑦, genes related to plant hormones; Group ⑧, genes related to protein and lipid metabolism; and Group ⑨, genes related to sugar metabolism. Category IV: Genes related to biological processes and unknown functions, including Group ⑩, genes related to other processes or unannotated. (**b**) Part of the protein–protein interaction (PPI) network associated with ADL and AAT genes for DSF in the WSGP. (**c**) Part of the protein–protein interaction (PPI) network associated with ADL and AAT genes for DFM in the WSGP. The blue ball represents the top 10% of nodes of the betweenness centrality (BC) value.

The identified genes are candidate genes before their functions have been demonstrated. A relatively thorough detection of the gene–alleles makes it possible to explore the functional composition of the gene system for a trait. According to SoyBase (https://www.soybase.org (accessed on 1 January 2021)), the identified 124–141 genes, out of a total of 661 genes of the six traits (some shared among traits) (Figure S2), were annotated based on their functions, which were grouped further into a same set of four categories of 10 groups of biological processes for each trait, although different genes may involve a similar function among the traits. The detailed category-group list for each trait is presented in Table 3 and Tables S2–S8, Figure 2a. The total 661 genes were annotated as follows: Category I: Group ①, 61 genes related to flower development and growth; Group ②, 31 genes related to light and circadian rhythm; and Group ③, 20 genes related to temperature response. Category II: Group ④, 30 genes related to histone variants and chromosome modification; Group ⑤, 112 genes related to DNA methylation, transcription, and RNA processing; and Group ⑥, 88 genes related to signal transduction and transport. Category III: Group ⑦, 64 genes related to plant hormones; Group ⑧, 141 genes related to protein and lipid metabolism; and Group ⑨, 29 genes related to sugar metabolism. Category IV: Group ⑩, 240 genes related to other or unannotated processes. Each trait is defined by the above four categories of 10 groups of genes, including some with still unknown functions. This indicates that the six traits involved in the DSF and DFM responses to degree and duration of day-length and temperature were defined by different gene sets, but with similar category-groups of functions.

## 3. Discussion

### 3.1. Advantages of GASM-RTM-GWAS in Exploring the Gene–Allele System and Gene Network

To explore the complete genetic system of a trait, a large germplasm population has to be used, especially for understanding the multiple allele composition. RTM-GWAS has been demonstrated to be powerful [32,33,34,35,50,51,52,53,54]. In the present study, it was an innovation to use the genomic marker GASM to replace another genomic marker, SNPLDB, to identify the gene–allele system directly to avoid inference of candidate genes from QTLs, while also merging the two-step process into a single step. As indicated above, GASM-RTM-GWAS was more powerful than SNPLDB-RTM-GWAS, in that more genes were identified compared with the results in [10], because the number of GASM–alleles better fit that of the causal genes owing to the extra-large SNPLDB segments and corresponding extra alleles being avoided. An additional advantage is that the GASM marker fits all populations that use the same reference genome, making the gene–allele results comparable among different studies.

The relative completeness and accuracy in identifying genes with their alleles from GASM-RTM-GWAS made possible the identification of further results on the population. These include the establishment of the gene–allele matrix to demonstrate the gene–allele structure of each variety and the whole population [37,55], population evolutionary genetic study through direct comparisons among the matrices of derived subpopulations, optimal cross prediction of the population and subpopulations [56], gene network exploration of the population, and the identification of major genes with their major alleles. Without the relatively thorough identification of the gene–allele system, these extended results are not possible. In addition, more genetic information can be obtained from the identified gene–allele system, such as the allele frequency changes in the evolutionary processes and G × E interactions of each gene–allele as well as the whole gene–allele system.

In addition, because the *R*^2^ and probability (*-lg*(*p*) value) of all of the identified genes in global soybeans were explored relatively thoroughly using GASM-RTM-GWAS, the importance of the identified genes and their alleles in the gene–allele system was evaluated [30]. For example, in the present global soybean population, 141 and 135 DSF and DFM genes were identified, respectively, and the individual phenotypic contributions were evaluated. Among these genes, six “*E*” genes (*E1*, *E2, E3, E4*, *E9*, and *E10* [43,44,46,57,58,59]), *DT1* [17], *J* [16], and *FT* [13] family genes were found near the present detected genes; especially, *Glyma06g23580* for DSF was close to *E1*. Likewise, *Glyma04g07430* and *Glyma04g04810* were near the *J* gene, and *Glyma19g34740* was near the *Dt1* (*TFL1b*) gene. However, some of the previously identified genes were not found to be important in the present global soybean germplasm population; therefore, the present results may have provided a check for further evaluating the relative importance of the previously identified DSF and DFM genes/alleles, which was impossible owing to the lack of multiple allele information.

### 3.2. Comparison of Gene–Allele Matrices between Ancestral and Filial Subpopulations as a New but Simple Approach in Exploring Evolutionary Drives

In the present study, the active factors of geographic adaptation and MG-set expansion included new allele emergence and old allele exclusion in addition to allele inheritance or migration. In each geographic region, new alleles emerged, with some being shared among the regions, and the total emerged alleles in the extended regions were less than the sum of the extended complex regions for all six traits, but old alleles were excluded in each region, with the sum of all regions being almost zero or no excluded alleles for the extended complex regions in comparison with those in the O region (ABCD vs. O in Table 4). Likewise, in each new MG-set, no new allele emerged, with the total emerged allele being zero, while old alleles were excluded in each MG-set, with some overlapping in new MG-sets, and the total excluded alleles were less than the sum of the total new MG-sets for all six traits (EL vs. P in Table 4). About 90% of alleles in the extended regions or expanded MG-sets were inherited from the O region or primary MG-set, while 0–11% of alleles emerged or were excluded, which caused the soybean to adapt to all environments around the world and new MG-sets to form. However, the phenotypic changes, such as DSF ranging from 26.4 to 70.6 d (37.0 to 68.5% changes) among the eco-regions and 23.0 to 77.5 d (41.2 to 98.2% changes) among MG-sets (Table S1, Figure S3), were not consistent with the allele changes of 0–11% (including both positive and negative alleles). Therefore, allele recombination based on allele changes is an important genetic drive, especially when accompanying gene interactions. Therefore, in addition to allele inheritance (migration), the population evolution motivator was not due only to new allele emergence, but to new allele emergence plus old allele exclusion and allele recombination. New gene interactions might form, which implies remarkable potential for evolution in a self-pollinated germplasm population (in a cross-pollinated population, the population genotypic structure maintains equilibrium depending on gene frequencies).

The above results were obtained from comparisons among gene–allele matrices between the original subpopulation (O for geographic region and P for MG-set) and the derived subpopulations, which were based on a relatively thorough identification of genes with their alleles through GASM-RTM-GWAS. This analysis would have been impossible without the relatively complete gene–allele identification. In previous studies on evolutionary mechanisms, the comparison of gene frequencies was usually used, but the emerged and excluded genes with their alleles were not detected, and thus were not included in those comparisons. In other words, the important events/information in evolution studies were neglected. Therefore, the comparison of gene–allele matrices among related consecutive populations based on GASM-RTM-GWAS is a new and exact, but simple approach to explore evolutionary drives or evolutionary mechanisms [10,37,51]. Furthermore, in addition to new allele emergence and old allele exclusion based on GASM-RTM-GWAS, the changes in allele frequency can be evaluated to explore natural vs. artificial selection functions.

### 3.3. Gene Interaction Network and Important Gene–Alleles of the Growth Period Eco-Traits of Global Soybeans

To understand how genes with different functions work together, the identified genes combined with the information collected from the STRING data center [60] were used for PPI analysis [40]. The 174 DSF, ADL_DSF_, and AAT_DSF_ genes demonstrated PPI effects, forming four PPI networks; three were completely connected with obvious node genes, while one was partially connected but without an obvious node gene (Figure 2b). However, the 175 DFM, ADL_DFM_, and AAT_DFM_ genes formed a larger network containing 145 (82.9%) genes that were completely connected with nodes, while the remained genes were partially connected without a node as another separate network (Figure 2c). This indicates that the regulatory modes were different between DSF and DFM, but both regulation patterns showed similar characteristics.

In the PPIs of DSF and DFM, seven and nine genes were related to light and circadian rhythm, respectively, indicating that light and circadian rhythm played an important role not only in DSF, but for both. While 12 genes related to temperature response were in the DSF PPI, only 5 genes were in the DFM PPI, indicating that temperature was important in the whole growth process, but was more important in DSF. In addition, the other genes in the PPIs of DSF and DFM were the protein- and lipid-metabolism-related genes and DNA methylation, transcription, and RNA-processing-related genes, which accounted for a large portion, followed by plant-hormone-related genes and signal-transduction- and transportation-related genes.

In addition, the genes with high betweenness centrality [61] (BC, the measure of node/hub importance) values were important to PPI. In DSF, the top five genes with high BC (*Glyma03g40780, Glyma03g0239, Glyma06g13320, Glyma02g45790,* and *Glyma04g16180*) were involved in genes related to protein and lipid metabolism; light and circadian rhythm; temperature response; DNA methylation, transcription, and RNA processing; signal transduction; and transport. In DFM, the top five high BC genes (*Glyma12g35580, Glyma13g22420, Glyma18g05730, Glyma18g01330,* and *Glyma20g27950*) were mainly involved in histone variants and genes related to protein and lipid metabolism. These results suggest that the genetic systems of the six growth-period-related traits are a complex gene system composed of different gene functions or networks, which should be further explored (Figure 2b,c).

For the identification of important genes and their alleles from the 661 genes and 1876 alleles, the alleles that can meet two or more of the following criteria were considered: (i) genes with a large contribution to PV (*R*^2^ ≥ 1.5%), (ii) genes with a top 10% BC score in the PPI network, (iii) genes with new emerged alleles, and (iv) genes shared among traits. For DSF, ADL_DSF_, and AAT_DSF_, 62 alleles from 20 genes were nominated, including 35 inherited and 27 emerged alleles. Among DFM, ADL_DFM_, and AAT_DFM_, 31 alleles from 12 genes were nominated, including 22 inherited and 9 emerged alleles. It is evident that the nominated important genes and alleles were from a global soybean population, and some of the previously reported genes (but without allele information) were included in the global nomination, while most of the previously reported genes were not included. Therefore, the presently nominated genes and alleles are particularly worth studying further (Table S9).

## 4. Materials and Methods

### 4.1. Plant Materials and Field Experiments

A total of 354 soybean varieties from 27 countries were selected as a representative sample of the world soybean germplasm population (WSGP) from the germplasm storage at the National Center for Soybean Improvement, Nanjing Agricultural University, Nanjing, China. According to a previous study by Liu et al. [4], the source of the materials was grouped into five regions, coded as O, A, B, C, and D (please refer to Table 4 notes for the detailed codes of the sub-regions). “O” represents the materials that came from the center of origin of the soybean in China, including HCHN and SCHN; “A” represents the materials from the northern dissemination route, including NCHN, RUFE, and SSWE; “B” represents the materials from the eastern dissemination route, including KORP and JPAN; “C” represents the materials from the southern dissemination route, including SEAS, SASI, and AFRI; and “D” represents the materials from the Western dissemination route, including NNAM, SNAM, and CSAM. The MG-set system of soybeans was first established in 1944 when MG I–MG VII was defined, and then the earlier MGs (MG 000–MG 0) and later MGs (MG VIII–MG X) were developed and added to the system. The soybean accessions include MG 000–X, with 13 MGs altogether [11], which were grouped into three MG-sets and designated as E (000–0), P (I–VII), and L (VIII–X) MG-sets.

The global soybeans were tested in 2015 spring (April 23 sowing), 2016 spring (April 25 sowing), 2016 summer (June 18 sowing), and 2017 summer (June 22 sowing) at the Jiangpu Experimental Station (32°07′ N, 118°62′ E), Jiangpu, Nanjing, China. A randomized complete blocks design was used with two replications, drill sowing, row length of 2 m, row spacing of 0.4 m, 10 seedlings per row, and conventional field management.

### 4.2. Measurement of Growth Period Traits and Their Required ADL and AAT

According to Fehr and Caviness [62], the emergence date (Ve), first flowering date (R1), and maturity date (R8) were recorded for all of the tested samples in the experiments, from which DSF and DFM were calculated.

Th day-length and temperature data were obtained from the Public Service of Nanjing Meteorological Bureau. The daily maximum and minimum temperature were used to calculate the daily average temperature, from which the daily active temperature was calculated as the accumulated daily average temperature over all of the days, except for those days whose average temperature was less than 10 °C. Likewise, the daily sunrise and sunset time were obtained, from which the day-length was calculated from their difference.

The required ADL and AAT for DSF and DFM were calculated from the summation of daily day-length and daily active temperature, which were designated as ADL_DSF_ and AAT_DSF_ and ADL_DFM_ and AAT_DFM_, respectively.

### 4.3. Statistical Analysis

For DSF and DFM and their required ADL and AAT of the four environments, eight blocks were calculated using SAS/STAT 9.4 software package (SAS Institute Inc., Cary, NC, USA). The PROC UNIVARIATE was used to perform descriptive statistical analysis of the traits and the significance between the subpopulations was calculated using the t-criterion. PROC GLM was used to perform variance analysis and regression analysis for the total data. The statistical model for analysis of variance is as follows: $y_{ijk}=\mu +t_{i}+g_{k}+r_{j\left(i\right)}+{\left(gt\right)}_{ik}+\varepsilon_{ijk}$, where μ is the population mean, $g_{k}$ is the effect of the *k*th genotype, $t_{i}$ is the effect of the *i*th environment, $r_{j\left(i\right)}$ is the *j*th block effect in the *i*th environment, ${\left(gt\right)}_{ik}$ represents the G × E effect between the *i*th genotype and *j*th environment, and $\varepsilon_{ijk}$ is the residual. The heritability was calculated as follows: $h^{2}=\sigma_{g}^{2}/\left(\sigma_{g}^{2}+\sigma_{ge}^{2}/n+\sigma_{\varepsilon }^{2}/rn\right)$, where $\sigma_{g}^{2}$ is the genetic variance, $\sigma_{ge}^{2}$ is the variance of G × E, $\sigma_{\varepsilon }^{2}$ is the error variance, *n* is the number of environments, and *r* is the number of replications in each environment. The genetic coefficient of variation is calculated as $GCV=\sigma_{g}/\mu$, where *μ* is the population mean [63,64].

In testing the significance of allele exclusion, the binomial probability of sampling error is estimated as *P_0_* = *C_n_^0^p^0^q^n^*, where *P_0_* is the sampling probability of all individuals without this allele in the subgroup; *p* = allele probability of the excluded allele, *q* = *1* − *p*, *n* = subgroup size. The *p*-value here is estimated using the allele frequency of the old subgroup of a specific allele [10].

### 4.4. SNP Genotyping and GASM Assembly

The restriction-site-associated DNA sequencing (RAD-seq) was conducted at BGI Tech, Shenzhen, China for genotyping the WSGP, which has been reported in detail by Liu et al. [4]. A total of 97,706 SNPs were confirmed for the 354 soybean varieties. All SNPs in a gene were assembled directly into GASM based on the reference genome Williams 82.a1.v1.1 (http://www.soybase.org (accessed on 1 January 2021)) [65]. The GASMs were generated using RTM-GWAS software with the haplotype/allele MAF less than 0.01 being superseded with a highest frequency haplotype/allele [30]. In total, 7801 GASMs with 18,111 haplotypes/alleles, each GASM with 2–8 haplotypes/alleles, in an average of 2.32 /GASM were obtained in the WSGP (Figure S4).

### 4.5. Identification of Gene–Alleles of DSF and DFM and Their Required ADL and AAT Using GASM-RTM-GWAS

Based on the GASMs, the RTM-GWAS procedure [30] was used to identify the gene–allele systems of DSF, DFM, ADL_DSF_, AAT_DSF_, ADL_DFM_, and AAT_DFM_. At both stages, the top 10 principal vectors of the genetic similarity coefficient matrix were constructed based on the GASMs and were used as covariates for the correction of the population structure bias. At the first stage of RTM-GWAS, using the general linear regression in the single-locus model, 6530, 6507, 6574, 6193, 6529, and 6550 GASMs were pre-selected at the significance level of *p* < 0.05 for the six traits, respectively. At the second stage of RTM-GWAS, the stepwise regression featured the forward selection and backward elimination under the multi-locus model and identified 141, 135, 130, 130, 124, and 129 genes with 406, 390, 384, 384, 364, and 362 alleles, respectively, for a total of 789 genes and 2290 alleles, or 661 genes with 1876 alleles when duplicates were not included for the six traits. As this experiment is used for population genetic research, it is necessary to excavate the complete gene system of relevant traits as much as possible, and the default significance level of *p* < 0.05 is still used in the second step. If you want to obtain relevant genes for gene-cloning-related experiments, you can use the significance level after Bonferroni correction (a/m) as the significance level of each step in multiple stepwise regression to identify significantly associated genes, where a and m are significance level (0.05) and the number of candidate markers, respectively. The RTM-GWAS software was publicly obtained from https://github.com/njau-sri/rtm-gwas or https://gitee.com/njau-sri/rtm-gwas (accessed on 1 January 2021) [30].

The identified gene system was annotated according to SoyBase (http://www.soybase.org (accessed on 1 January 2021)) based on the reference genome Wm82.a1.v1.1, from the following databases: GO (Gene Ontology, http://geneontology.org/docs/download-ontology/ (accessed on 1 January 2021)) and TAIR (Arabidopsis Information Resource, http://www.arabidopsis.org/ (accessed on 1 January 2021)).

### 4.6. Analysis of Genetic Motivators in Geographic Adaptation and MG-Set Expansion of Global Soybeans

To reveal the gene–allele changes and evolutionary motivators of the six eco-traits from the center of origin to the various geographic regions and from the primary MG-set (I-VII) to the emerged-early MG-set (000-0) and emerged-late MG-set (VIII-X), the whole population gene–allele matrix for each trait was separated into their component submatrices. The inherited, excluded, and emerged alleles were calculated and compared directly to those of the center of origin and of the primary MG-set to evaluate the relative importance of the evolutionary motivators.

### 4.7. Prediction of Genetic Recombination Potentials of DSF and DFM and Their Required ADL and AAT in Global Soybeans

Based on the gene–allele matrix, the possible parent crosses with their progenies among all the accessions were simulated. A total of 2000 homozygous progenies were calculated under the linkage and independent assortment models [30]. The 25th and 95th percentile of a cross were used as the recombination potential indicator for comparisons between the crosses. To determine the transgressive crosses, low-parent heterosis and high-parent heterosis were calculated as LPT = F_25th_−LPV and HPT = F_95th_−HPV, respectively, where F_25th_ and F_95th_ are the 25th and 95th percentile value of a cross population, respectively, while LPV and HPV are the low and high parent values, respectively.

### 4.8. Gene Functional Annotation and Gene Network Analysis

The identified genes were annotated by GO analysis for their functional groups according to SoyBase (http://www.soybase.org (accessed on 1 January 2021)). To explore the gene interactions, protein sequences of the genes were obtained from the phytozome database (http://phytozome.jgi.doe.g.,ov (accessed on 1 January 2021)), then STRING (http://string-db.org (accessed on 1 January 2021)) [66] was used to generate PPI networks [67]. In the PPI network analysis, proteins are described as nodes/hubs and edges. The importance of each node is expressed by its centrality, including betweenness, degree, and closeness, whereas the edge is usually undirected and unweighted [61,68,69,70].

## 5. Conclusions

The DSF and DFM of the global soybean germplasm population were traced to their required accumulative day-length and active temperature to explore their eco properties. (i) Through an improved genome-wide association study with gene–allele sequence as markers (GASM-RTM-GWAS), the six gene–allele systems for DSF, DFM, ADL_DSF_, AAT_DSF_, ADL_DFM_, and AAT_DFM_ (124–141 genes with 362–406 alleles per trait) were explored. DSF shared more ADL and AAT contributions than DFM, while AAT contributed more than ADL to DSF, but vice versa for DFM. (ii) The genetic adaptation from the origin to the geographic sub-regions was characterized by allele emergence (mutation), while genetic expansion from primary maturity group (MG)-sets to early/late MG-sets featured allele exclusion (selection) without allele emergence in addition to inheritance (migration). (iii) Optimal crosses with transgressive segregations in both directions were predicted for breeding purposes, and allele recombination in soybean is an important evolutionary drive. (iv) Genes of the six traits were mostly trait-specific involved in four categories of 10 groups of biological functions. Therefore, GASM-RTM-GWAS showed potential in directly detecting causal genes with their alleles, identifying differential trait evolutionary drives, predicting recombination breeding potentials, and revealing population gene networks.

## Figures and Tables

**Table 1:** Summary of the detected gene–allele system conferring the six DSF- and DFM-related traits in the WSGP.

Gene–Allele	DSF		ADL_DSF_		AAT_DSF_	
	Main-Effect (*R*^2^ %)	Gene × Env. (*R*^2^ %)	Main-Effect (*R*^2^ %)	Gene × Env. (*R*^2^ %)	Main-Effect (*R*^2^ %)	Gene × Env. (*R*^2^ %)
Gene	76.85 (141, 0.00–6.95)	4.05 (11, 0.19–1.01)	75.35 (130, 0.00–6.77)	3.93 (4, 0.45–1.86)	73.76 (130, 0.00–7.32)	0.55 (3, 0.11–0.27)
LC-major gene	51.63 (17, 1.55–6.95)		45.32 (14, 1.61–6.77)	1.86 (1,1.86)	58.24 (19, 1.59–7.32)	
SC-major gene	25.22 (124, 0.00–1.42)	4.05 (11, 0.19–1.01)	30.03 (116, 0.00–1.39)	20.7 (3, 0.45–1.10)	15.52 (111, 0.00–1.16)	0.55 (3, 0.11–0.27)
Unmapped gene	18.85	0.15	20.45	0.17	25.44	0.15
*h^2^*	95.7	4.2	95.8	4.1	99.2	0.7
Positive allele	195 (0.04–20.83)	11 (1.67–20.29)	191 (0.21–239.12)	4 (23.24–123.88)	186 (1.56–473.94)	3 (87.37–473.94)
Negative allele	211 (−21.69 to–0.02)	21 (−20.29 to −1.12)	199 (−187.33 to −0.32)	5 (−123.88 to −3.21)	198 (−405.98 to −0.23)	5 (−289.27 to −34.43)
Total	406 (−21.69–20.83)	32 (−20.29–20.29)	390 (−187.33–239.12)	9 (−123.88–123.88)	384 (−405.98~473.94)	8 (−289.27–473.94)
Gene–allele	DFM		ADL_DFM_		AAT_DFM_	
	Main-effect (*R*^2^ %)	Gene × Env. (*R*^2^ %)	Main-effect (*R*^2^ %)	Gene × Env. (*R*^2^ %)	Main-effect (*R*^2^ %)	Gene × Env. (*R*^2^ %)
Gene	55.03 (135, 0.00–7.20)	18.11 (5, 1.34–7.36)	50.24 (124, 0.01–7.18)	20.36 (5, 1.46–8.32)	41.54 (129, 0.01–5.71)	28.86 (20, 0.41–6.71)
LC-major gene	22.40 (8, 1.59–7.20)	16.77 (4, 2.12–7.36)	16.29 (5, 1.56–7.18)	18.90 (4, 1.51–8.32)	10.34 (3, 1.56–5.71)	18.55 (5, 2.37–6.71)
SC-major gene	32.63 (127, 0.00–1.44)	1.34 (1, 1.34)	33.95 (119, 0.01–1.45)	1.46 (1, 1.46)	31.20 (126, 0.01–1.34)	10.31 (15, 0.41–1.43)
Unmapped gene	25.67	0.69	28.26	0.54	29.36	0.04
*h^2^*	80.7	18.8	78.5	20.9	70.9	29
Positive allele	185 (0.01–17.94)	11 (0.96–9.42)	186 (0.13–354.47)	11 (0.13–77.63)	178 (0.14–609.99)	33 (3.80–610.00)
Negative allele	199 (−24.63 to–0.01)	9 (−9.68 to −1.08)	178 (−261.34 to−0.38)	9 (−143.00 to −10.50)	184 (−596.44 to −0.61)	34 (−596.44 to −17.12)
Total	384 (−24.63–17.94)	20 (−9.68–9.42)	364 (−261.34–354.47)	20 (−143.00–77.63)	362 (−596.44–609.99)	67 (−596.44–610.00)

Note: DSF: days from sowing-to-flowering; ADL_DSF_: DSF required accumulative day-length; AAT_DSF_: DSF required accumulative active temperature; DFM: days from flowering-to-maturity; ADL_DFM_: DFM required accumulated day-length; AAT_DFM_: DFM required accumulative active temperature. Main-effect: main-effect gene. Gene × Env.: gene by environment interaction. *R*^2^: genetic contribution of a gene. LC-major gene: large-contribution major gene with *R*^2^ ≥ 1.5%. SC-major gene: small-contribution major gene with *R*^2^ < 1.5%. Unmapped gene: unmapped minor genes. In parentheses of gene rows, the first number is the number of identified genes, followed by a range of single gene contributions to phenotypic variance. In parentheses of allele rows is the range of single allele effects with the unit of d for DSF and DFM, d · h for ADL, and d · °C for AAT.

**Table 2:** Shared genes among DSF, ADL_DSF_, and AAT_DSF_ and among DFM, ADL_DFM_, and AAT_DFM_ in the WSGP.

Common Genes	Gene Code	*R* ^2^	Gene Code	*R* ^2^	Gene Code	*R* ^2^	Cloned Gene Reported
*Glyma01g22830*	***^§^*** ***g-DSF-01-2***	3.05	***g-ADL_DSF_-01-3***	2.90	***g-AAT_DSF_-01-3***	4.26	
*Glyma02g04190*	***g-DSF-02-3***	2.98	*g-ADL_DSF_-02-2*	1.39	***g-AAT_DSF_-02-2***	3.12	
*Glyma06g23580*	***g-DSF-06-5***	4.25	***g-ADL_DSF_-06-3***	4.24	***g-AAT_DSF_-06-6***	4.41	*E1* (696.3 kb) [43]
*Glyma08g03210*	***g-DSF-08-1***	6.07	***g-ADL_DSF_-08-1***	6.04	***g-AAT_DSF_-08-1***	6.14	
*Glyma10g26450*	***g-DSF-10-3***	1.69	*g-ADL_DSF_-10-4*	0.17	***g-AAT_DSF_-10-2***	1.79	*E4* (2112.8 kb) [44]
*Glyma13g07110*	***g-DSF-13-1***	5.60	***g-ADL_DSF_-13-1***	5.67	***g-AAT_DSF_-13-2***	6.25	
*Glyma16g03320*	***g-DSF-16-2***	2.24	***g-ADL_DSF_-16-1***	2.70	***g-AAT_DSF_-16-2***	2.30	*LHY1a* (1306.3 kb) [45]
*Glyma17g08460*	***g-DSF-17-4***	6.95	***g-ADL_DSF_-17-3***	6.77	***g-AAT_DSF_-17-3***	7.32	
*Glyma17g09500*	***g-DSF-17-6***	2.26	***g-ADL_DSF_-17-4***	2.72	***g-AAT_DSF_-17-4***	2.30	
9		35.09		32.60		37.89	
*Glyma03g27970*	*g-DSF-03-4*	0.16	*g-ADL_DSF_-03-4*	0.40			
*Glyma09g31087*	*g-DSF-09-6*	0.10	*g-ADL_DSF_-09-3*	0.37			
*Glyma14g37330*	*g-DSF-14-7*	0.08	*g-ADL_DSF_-14-6*	0.25			
*Glyma20g08091*	*g-DSF-20-1*	0.04	*g-ADL_DSF_-20-3*	0.07			
4		0.38		1.09			
*Glyma02g03920*	*g-DSF-02-1*	1.25			***g-AAT_DSF_-02-1***	1.60	
*Glyma02g37350*	*g-DSF-02-8*	0.63			*g-AAT_DSF_-02-6*	0.65	
*Glyma02g48010*	***g-DSF-02-11***	2.08			***g-AAT_DSF_-02-9***	2.10	
*Glyma04g36240*	*g-DSF-04-3*	1.06			*g-AAT_DS_F-04-5*	1.16	
*Glyma05g31250*	*g-DSF-05-3*	0.19			*g-AAT_DSF_-05-2*	0.09	
*Glyma06g36380*	***g-DSF-06-6***	1.93			***g-AAT_DSF_-06-8***	1.91	
*Glyma06g47590*	*g-DSF-06-9*	0.11			*g-AAT_DSF_-06-12*	0.04	
*Glyma07g09170*	*g-DSF-07-2*	0.14			*g-AAT_DSF_-07-2*	3.6 × 10^−3^	
*Glyma09g02470*	***g-DSF-09-1***	2.81			***g-AAT_DSF_-09-1***	2.95	
*Glyma09g25215*	*g-DSF-09-5*	0.33			*g-AAT_DSF_-09-4*	1.6 × 10^−3^	
*Glyma09g34850*	***g-DSF-09-7***	1.79			***g-AAT_DSF_-09-8***	1.85	
*Glyma10g35960*	*g-DSF-10-7*	0.97			*g-AAT_DSF_-10-4*	0.99	*E2* (545.6 kb) [46]
*Glyma11g14500*	*g-DSF-11-3*	1.42			***g-AAT_DSF_-11-3***	1.79	
*Glyma11g19670*	*g-DSF-11-5*	1.04			*g-AAT_DSF_-11-4*	1.11	
*Glyma12g08000*	***g-DSF-12-2***	1.59			***g-AAT_DSF_-12-1***	1.73	*PRR3b* (152.2 kb) [47]
*Glyma12g34830*	*g-DSF-12-4*	0.36			*g-AAT_DSF_-12-3*	0.08	
*Glyma13g21340*	*g-DSF-13-4*	0.01			*g-AAT_DSF_-13-3*	0.07	
*Glyma13g25480*	*g-DSF-13-5*	0.01			*g-AAT_DSF_-13-5*	1.3 × 10^−3^	
*Glyma15g08420*	*g-DSF-15-2*	0.08			*g-AAT_DSF_-15-3*	0.04	
*Glyma15g34840*	***g-DSF-15-5***	3.05			***g-AAT_DSF_-15-7***	3.22	
*Glyma18g08410*	***g-DSF-18-2***	1.74			***g-AAT_DSF_-18-3***	1.61	
*Glyma18g17395*	***g-DSF-18-4***	1.55			***g-AAT_DSF_-18-5***	1.59	
*Glyma18g17515*	*g-DSF-18-5*	0.02			*g-AAT_DSF_-18-6*	0.02	
23		24.16				24.61	
*Glyma06g32870*			*g-ADL_DSF_-06-4*	0.08	*g-AAT_DSF_-06-7*	0.27	
*Glyma09g28620*			*g-ADL_DSF_-09-2*	0.07	*g-AAT_DSF_-09-6*	3.5 × 10^−3^	
*Glyma18g26120*			*g-ADL_DSF_-18-4*	3.7E-03	*g-AAT_DSF_-18-9*	3.8 × 10^−3^	
*Glyma19g23640*			*g-ADL_DSF_-19-3*	0.07	*g-AAT_DSF_-19-1*	0.02	
4				0.22		0.30	
Specific gene	94	14.75	96	31.65	88	10.49	
Total	141	74.38	130	65.56	130	73.29	
*Glyma02g00371*	***g-DFM-02-1***	2.66	***g-ADL_DFM_-02-1***	2.72	*g-AAT_DFM_-02-1*	1.33	
*Glyma07g40260*	*g-DFM-07-8*	0.69	*g-ADL_DFM_-07-8*	1.00	*g-AAT_DFM_-07-7*	0.57	
*Glyma08g15870*	*g-DFM-08-4*	1.21	*g-ADL_DFM_-08-3*	1.45	*g-AAT_DFM_-08-5*	1.27	
*Glyma12g06580*	*g-DFM-12-2*	0.77	*g-ADL_DFM_-12-2*	0.18	*g-AAT_DFM_-12-2*	0.28	
*Glyma12g06950*	*g-DFM-12-4*	0.06	*g-ADL_DFM_-12-3*	0.01	*g-AAT_DFM_-12-4*	0.15	
*Glyma13g09470*	*g-DFM-13-3*	1.25	*g-ADL_DFM_-13-1*	1.14	*g-AAT_DFM_-13-2*	1.27	
*Glyma13g25480*	***g-DFM-13-8***	2.59	***g-ADL_DFM_-13-4***	2.16	***g-AAT_DFM_-13-7***	3.07	
*Glyma19g34740*	***g-DFM-19-9***	2.31	***g-ADL_DFM_-19-6***	2.67	*g-AAT_DFM_-19-6*	0.69	*DT1* (*TFL1b*, 2633.3 Kb) [17]
8		11.54		11.33		8.63	
*Glyma03g02940*	*g-DFM-03-1*	0.08	*g-ADL_DFM_-03-2*	0.10			
*Glyma03g06483*	*g-DFM-03-2*	1.09	*g-ADL_DFM_-03-4*	1.31			
*Glyma06g40670*	*g-DFM-06-4*	0.49	*g-ADL_DFM_-06-7*	0.60			
*Glyma07g09420*	*g-DFM-07-3*	1.44	***g-ADL_DFM_-07-2***	1.56			
*Glyma10g29970*	*g-DFM-10-3*	0.76	*g-ADL_DFM_-10-6*	0.97			
*Glyma11g10800*	*g-DFM-11-2*	0.15	*g-ADL_DFM_-11-2*	0.35			
*Glyma13g28880*	*g-DFM-13-12*	0.17	*g-ADL_DFM_-13-5*	0.34			
*Glyma19g23740*	*g-DFM-19-3*	1.10	*g-ADL_DFM_-19-2*	0.93			
*Glyma19g30690*	***g-DFM-19-6***	7.20	***g-ADL_DFM_-19-4***	7.18			
*Glyma19g33210*	*g-DFM-19-7*	0.05	*g-ADL_DFM_-19-5*	0.02			
*Glyma20g03100*	*g-DFM-20-1*	0.24	*g-ADL_DFM_-20-2*	0.65			
11		12.77		14.01			
*Glyma05g26620*	*g-DFM-05-6*	0.05			*g-AAT_DFM_-05-4*	0.01	
*Glyma11g13111*	*g-DFM-11-3*	0.01			*g-AAT_DFM_-11-4*	0.12	
*Glyma12g06620*	*g-DFM-12-3*	0.21			*g-AAT_DFM_-12-3*	0.15	
*Glyma16g25500*	***g-DFM-16-3***	2.12			*g-AAT_DFM_-16-2*	0.99	*E9* (1225.6 kb) [48]
4		*2.39*				1.27	
*Glyma05g04561*			*g-ADL_DFM_-05-1*	1.22	*g-AAT_DFM_-05-2*	0.58	
*Glyma06g19480*			*g-ADL_DFM_-06-5*	0.52	*g-AAT_DFM_-06-6*	0.36	
*Glyma07g10541*			*g-ADL_DFM_-07-3*	0.18	*g-AAT_DFM_-07-3*	0.03	
*Glyma07g29650*			*g-ADL_DFM_-07-6*	0.62	*g-AAT_DFM_-07-5*	0.17	
*Glyma08g15400*			*g-ADL_DFM_-08-2*	0.03	*g-AAT_DFM_-08-4*	0.10	
*Glyma16g33100*			*g-ADL_DFM_-16-8*	1.39	*g-AAT_DFM_-16-5*	0.48	
*Glyma17g01160*			*g-ADL_DFM_-17-1*	0.2	*g-AAT_DFM_-17-1*	0.05	
*Glyma17g19084*			*g-ADL_DFM_-17-5*	0.1	*g-AAT_DFM_-17-5*	0.04	
8				4.26		1.81	
Specific gene	105	26.59	86	18.43	96	28.33	
Total	135	53.29	124	48.03	129	40.04	

Note: Gene code: for example, g-DSF-06-4, -06 represents chromosome 6 and -4 represents its order on the chromosome according to its physical position. The position corresponds to the Williams 82 reference genome version 1 (Wm82.a1). §: bold gene code means *R*^2^ of gene ≥1.5%.

**Table 3:** The functional categories and their groups of identified genes for six DSF- and DFM-related traits in the WSGP.

Category -Group		DSF (*R*^2^ %)		ADL_DSF_ (*R*^2^ %)		AAT_DSF_ (*R*^2^ %)		DFM (*R*^2^ %)		ADL_DFM_ (*R*^2^ %)		AAT_DFM_ (*R*^2^ %)	
I	①	27 (12.9)	11 (3.5)	28 (13.4)	14 (8.6)	23 (12.9)	10 (3.3)	19 (3.0)	14 (2.4)	17 (6.3)	7 (3.7)	24 (4.6)	13 (1.4)
	②		7 (4.5)		8 (1.2)		5 (5.2)		2 (0.4)		10 (2.6)		6 (1.9)
	③		9 (4.9)		6 (3.7)		8 (4.4)		3 (0.2)		0 (0.0)		5 (1.3)
II	④	41 (18.4)	4 (4.0)	53 (24.4)	6 (4.5)	48 (18.8)	8 (4.2)	38 (16.6)	10 (6.5)	47 (20.2)	6 (3.2)	45 (14.5)	7 (5.1)
	⑤		18 (10.2)		33 (15.0)		23 (10.8)		12 (5.0)		20 (8.6)		27 (6.7)
	⑥		19 (4.2)		14 (4.9)		17 (3.8)		16 (5.1)		21 (8.4)		11 (2.7)
III	⑦	61 (38.1)	17 (10.5)	48 (33.2)	12 (9.7)	38 (32.6)	8 (8.3)	53 (16.9)	12 (3.3)	48 (19.9)	14 (6.4)	37 (10.9)	2 (1.4)
	⑧		35 (18.2)		32 (15.8)		26 (16.3)		36 (12.6)		24 (10.6)		14 (3.7)
	⑨		9 (9.4)		4 (7.6)		4 (8.0)		5 (0.9)		10 (3.0)		21 (5.8)
IV	⑩	47 (27.9)	47 (27.9)	41 (29.1)	41 (29.1)	53 (27.6)	53 (27.6)	54 (27.5)	54 (27.5)	42 (18.8)	42 (18.8)	51 (19.7)	51 (19.7)
Total			176 (87.9)		170 (100.1)		162 (91.9)		164 (63.9)		154 (65.3)		157 (49.7)

Note: The numbers in parentheses represent the sum of *R*^2^ for a certain category or group. The four gene ontology categories with their groups are as follows: Category I: Genes related to flowering, seed and stem development, or response to light and temperature stimulation, including Group ①, genes related to flower development and growth; Group ②, genes related to light and circadian rhythm; and Group ③, genes related to temperature response. Category II: Translocation signal transduction; defense response; and genes related to DNA methylation, transcription, RNA processing, and chromosome modification, including Group ④, genes related to histone variants and chromosome modification; Group ⑤, genes related to DNA methylation, transcription, and RNA processing; and Group ⑥, genes related to signal transduction and transport. Category III: Primary metabolism genes related to secondary metabolism, including Group ⑦, genes related to plant hormones; Group ⑧, genes related to protein and lipid metabolism; and Group ⑨, genes related to sugar metabolism. Category IV: Genes related to biological processes and unknown functions, including Group ⑩, genes related to other processes or unannotated.

**Table 4:** Changes of genes–alleles from the center of origin to the derived geographic regions and from the primary MG-set to expanded MG-sets.

Trait	Contrast	Total		Inherent		Emerged		Excluded	
		Allele No.	Gene	Allele No.	Gene	Allele No.	Gene	Allele No.	Gene
Geographic adaptation									
DSF	O	364 (188,176)	141						
	A vs.O	352 (186,166)	141	329 (171,158)	141	23 (15, 8) (8,5) *	22	35 (17,18) (15,10) *	29
	B vs. O	359 (190,169)	141	336 (177,159)	141	23 (13,10) (7,3) *	22	28 (11,17) (6,6) *	26
	C vs. O	362 (187,175)	141	330 (170,160)	141	32 (17,15) (10,10) *	29	34 (18,16) (12,7) *	30
	D vs. O	361 (184,177)	141	334 (171,163)	141	27 (13,14) (5,4) *	24	30 (17,13) (11,8) *	26
	ABCD vs.O	405 (210,195)	141	363 (187,176)	141	42 (23,17) (23,17) *	37	1 (1,0) (1,0) *	1
ADL_DSF_	O	340 (181,159)	130						
	A vs.O	336 (177,159)	130	307 (165,142)	130	29 (12,17) (9,11) *	28	33 (16,17) (7,10) *	31
	B vs. O	347 (185,162)	130	319 (172,147)	130	28 (13,15) (9,7) *	26	21 (9,12) (3,5) *	20
	C vs. O	365 (189,176)	130	326 (175,151)	130	39 (14,25) (9,13) *	34	14 (6,8) (1,4) *	14
	D vs. O	346 (181,165)	130	312 (171,141)	130	34 (10, 24) (6,12) *	30	28 (10,18) (5,9) *	27
	ABCD vs.O	390 (199,191)	130	340 (181,159)	130	50 (18, 32) (18,32) *	44	(0,0)	0
AAT_DSF_	O	342 (182,160)	130						
	A vs.O	336 (178,158)	130	312 (166,146)	130	24 (12,12) (6,7) *	23	30 (16,14) (13,8) *	27
	B vs. O	346 (186,160)	130	318 (173,145)	130	28 (13,15) (8,6) *	25	24 (9,15) (7,4) *	23
	C vs. O	354 (182,172)	130	318 (168,150)	130	36 (14,22) (4,14) *	33	24 (14,10) (9,3) *	20
	D vs. O	344 (179,165)	130	315 (169,146)	130	29 (10,19) (5,6) *	24	27 (13,14) (8,7) *	24
	ABCD vs.O	383 (198,185)	130	341 (182,159)	130	42 (16,26) (16,26) *	38	1 (0,1) (0,0) *	1
DFM	O	340 (177,163)	135						
	A vs.O	348 (177,171)	135	312 (161,151)	135	36 (16,20) (7,9) *	33	28 (16,12) (8,8) *	26
	B vs. O	347 (180,167)	135	316 (164,152)	135	31 (16,15) (10,8) *	28	24 (13,11) (4,7) *	23
	C vs. O	340 (174,166)	135	314 (163,151)	135	26 (11,15) (3,5) *	24	26 (14,12) (7,6) *	24
	D vs. O	345 (175,170)	135	315 (158,157)	135	30 (17,13) (8,7) *	27	25 (19,6) (12,3) *	23
	ABCD vs.O	384 (199,185)	135	340 (177,163)	135	44 (22,22) (22,22) *	39	(0,0)	0
ADL_DFM_	O	323 (157,166)	124						
	A vs.O	325 (155,170)	124	299 (142,157)	124	26 (13,13) (6,10) *	24	24 (15,9) (11,7) *	21
	B vs. O	328 (162,166)	124	300 (146,154)	124	28 (16,12) (7,4) *	26	23 (11,12) (5,6) *	21
	C vs. O	317 (158,159)	124	296 (146,150)	124	21 (12,9) (3,2) *	20	27 (11,16) (5,6) *	25
	D vs. O	320 (154,166)	124	292 (143,149)	124	28 (11,17) (10,9) *	26	31 (14,17) (8,12) *	29
	ABCD vs.O	364 (178,186)	124	323 (157,166)	124	41 (21,20) (21,20) *	36	(0,0)	0
AAT_DFM_	O	320 (162,158)	129						
	A vs.O	321 (167,154)	129	292 (152,140)	129	29 (15,14) (10,10) *	29	28 (10,18) (8,12) *	25
	B vs. O	325 (168,157)	129	302 (156,146)	129	23 (12,11) (4,3) *	22	18 (6,12) (4,5) *	17
	C vs. O	322 (163,159)	129	297 (151,146)	129	25 (12,13) (6,8) *	24	23 (11,12) (3,5) *	21
	D vs. O	327 (171,156)	129	301 (156,145)	129	26 (15,11) (8,5) *	24	19 (6,13) (4,6) *	18
	ABCD vs.O	362 (184,178)	129	320 (162,158)	129	42 (22,20) (22,20) *	38	(0,0)	0
MG expansion									
DSF	P	405 (211,194)	141						
	E vs. P	309 (161,148)	141	309 (161,148)	141	0 (0,0)	0	96 (50,46) (49,44) *	73
	L vs. P	353 (182,171)	141	352 (182,170)	141	1 (0,1) (0,1) *	1	53 (29,24) (27,23) *	47
	EL vs. P	381 (196,185)	141	380 (196,184)	141	1 (0,1) (0,1) *	1	25 (15,10) (15,10) *	11
ADL_DSF_	P	390 (199,191)	130						
	E vs. P	281 (153,128)	130	281 (153,128)	130	0 (0,0)	0	109 (46,63) (45,61) *	84
	L vs. P	336 (174,162)	130	336 (174,162)	130	0 (0,0)	0	54 (25,29) (25,29) *	42
	EL vs. P	360 (189,171)	130	360 (189,171)	130	0 (0,0)	0	30 (10,20) (10,20) *	27
AAT_DSF_	P	383 (198,185)	130						
	E vs. P	275 (145,130)	130	275 (145,130)	130	0 (0,0)	0	108 (53,55) (52,52) *	77
	L vs. P	337 (172,165)	130	336 (172,164)	130	1 (0,1) (0,1) *	1	47 (26,21) (26,18) *	36
	EL vs. P	363 (186,177)	130	362 (186,176)	130	1 (0,1) (0,1) *	1	21 (12,9) (12,9) *	18
DFM	P	384 (199,185)	135						
	E vs. P	292 (152,140)	135	292 (152,140)	135	0 (0,0)	0	92 (47,45) (47,43) *	70
	L vs. P	332 (165,167)	135	332 (165,167)	135	0 (0,0)	0	52 (34,18) (32,18) *	42
	EL vs. P	359 (184,175)	135	359 (184,175)	135	0 (0,0)	0	25 (15,10) (15,10) *	24
ADL_DFM_	P	364 (176,186)	124						
	E vs. P	274 (134,140)	124	274 (134,140)	124	0 (0,0)	0	90 (44,46) (44,46) *	65
	L vs. P	293 (143,150)	124	293 (143,150)	124	0 (0,0)	0	71 (35,36) (35,33) *	56
	EL vs. P	323 (163,160)	124	323 (163,160)	124	0 (0,0)	0	41 (15,26) (15,26) *	37
AAT_DFM_	P	362 (184,178)	129						
	E vs. P	283 (148,135)	129	283 (148,135)	129	0 (0,0)	0	79 (36,43) (35,43) *	59
	L vs. P	302 (157,145)	129	302 (157,145)	129	0 (0,0)	0	60 (27,33) (27,31) *	50
	EL vs. P	339 (175,164)	129	339 (175,164)	129	0 (0,0)	0	23 (9,14) (9,14) *	22

Note: In the Trait column, GS: geographic sub-population. “O” represents the center of origin; “A” represents Northeast China, far-east of Russia, and southern Sweden; “B” represents the Korea Peninsular and Japan Islands; “C” represents Southeast Asia, South Asia, and Africa; “D” represents northern North America, southern North America, and Central and South America. MG: maturity group; “E” represents the early MG-set (MG 000 ~ 0); “P” represents the primary MG-set (MG I ~ VII); “L” represents the late MG-set (MG VIII ~ X). In the Contrast column, A vs. O means the comparison of alleles between A and O, similarly for B vs. O and C vs. O, as well as for D vs. O and ABCD vs. O; E vs. P means the comparison of alleles between E and P, similarly for L vs. P and EL vs. P. In columns of Allele no., the number outside the parentheses is the number of alleles and the number inside the parentheses is the number of negative (left) and positive (right) alleles. Gene: the number of genes involved. Inherent: alleles passed from O or P subpopulation. In the Emerged column and Excluded column, the number with * after the second parentheses indicates the significant alleles that emerged or were excluded in the respective comparisons.

**Table 5:** Predicted recombination potential for six DSF- and DFM-related traits under the linkage and independent assortment model in the WSGP.

Prediction Model	Trait	No. Crosses	25% Percentile				No. Crosses	LPT			
			Min.	Max.	Mean	*CV* (%)		Min.	Max.	Mean	*CV* (%)
Linkage model	DSF (d)	62,128	5.43	89.24	33.24	31.34	5149	−20.19	−0.09	−5.28	−54.59
	ADL_DSF (_d · h)	62,128	161.76	1314.71	489.67	31.53	6352	−202.57	−0.08	−78.61	−43.63
	AAT_DSF_ (d · °C)	62,128	245.25	2628.83	975.05	29.76	4663	−416.02	−0.72	−136.33	−52.21
	DFM (d)	62,128	20.36	108.65	72.78	16.71	942	−22.41	−0.10	−9.25	−42.40
	ADL_DFM_ (d·h)	62,128	443.29	1436.31	999.43	15.36	992	−307.52	−0.58	−143.73	−43.60
	AAT_DFM_ (d · °C)	62,128	774.41	2641.53	1898.16	16.44	1732	−643.04	−0.38	−299.76	−35.78
	Trait	Cross number	95% percentile				Cross number	HPT			
			Min.	Max.	Mean	*CV* (%)		Min.	Max.	Mean	*CV* (%)
	DSF (d)	62,128	21.98	140.03	58.42	28.77	2090	0.51	58.18	18.46	54.75
	ADL_DSF (_d · h)	62,128	328.37	1872.39	882.47	24.90	1742	0.42	632.14	227.99	57.05
	AAT_DSF (_d · °C)	62,128	661.21	3750.91	1679.24	26.21	1631	11.01	1120.67	404.14	61.25
	DFM (d)	62,128	50.83	153.23	107.97	11.97	19857	0.04	47.14	19.23	32.91
	ADL_DFM_ (d · h)	62,128	735.37	1995.88	1425.32	10.87	21821	0.38	743.29	232.89	42.62
	AAT_DFM_ (d · °C)	62,128	1400.68	3895.99	2712.61	9.94	27145	0.81	1545.11	420.20	46.29
Prediction model	Trait	Cross number	25% percentile				Cross number	LPT			
			Min.	Max.	Mean	*CV* (%)		Min.	Max.	Mean	*CV* (%)
Independent assortment model	DSF (d)	62,128	6.80	89.58	32.87	31.69	5784	−22.77	−0.04	−5.37	−49.71
	ADL_DSF (_d · h)	62,128	156.68	1319.42	474.07	32.49	8269	−212.94	−0.25	−88.18	−40.35
	AAT_DSF_ (d · °C)	62,128	244.29	2590.16	944.7.1	31.08	7041	−473.13	−1.57	−159.19	−44.65
	DFM (d)	62,128	26.15	108.65	70.80	17.66	1890	−22.54	−0.15	−10.92	−38.56
	ADL_DFM_ (d · h)	62,128	413.22	1436.31	986.99	15.48	1141	−309.67	−0.70	−144.60	−42.82
	AAT_DFM_ (d · °C)	62,128	622.26	2641.53	1847.34	17.18	2667	−680.01	−2.03	−321.39	−36.25
	Trait	Cross number	95% percentile				Cross number	HPT			
			Min.	Max.	Mean	*CV* (%)		Min.	Max.	Mean	*CV* (%)
	DSF (d)	62,128	21.98	154.33.	59.62	28.96	2633	0.10	76.37	24.15	56.71
	ADL_DSF (_d · h)	62,128	328.37	1936.45	919.74	24.32	2631	0.01	685.29	248.36	51.68
	AAT_DS F_ (d · °C)	62,128	661.21	3849.81	1756.04	25.22	2148	0.54	1187.69	439.01	57.75
	DFM (d)	62,128	50.83	159.41	112.79	12.03	27922	0.14	53.48	23.70	33.73
	ADL_DFM_ (d · h)	62,128	735.37	2066.57	1458.78	10.50	26383	0.95	646.95	246.47	35.88
	AAT_DFM_ (d · °C)	62,128	1400.68	4358.57	2842.92	10.99	37073	195	1763.93	559.27	43.49

Note: LPT: low-parent transgression; HPT: high-parent transgression.

## Data Availability

The data presented in this study are available in the Supplementary Materials.

## Supplementary Materials

The supporting information can be downloaded at https://www.mdpi.com/article/10.3390/ijms24119570/s1.
