# Supplementary material for: An Improved Genome-Wide Association Procedure Explores Gene–Allele Constitutions and Evolutionary Drives of Growth Period Traits in the Global Soybean Germplasm Population
Source: Int J Mol Sci. 2023 May 31;24(11):9570. doi: 10.3390/ijms24119570 (PMC10253808; doi:10.3390/ijms24119570)
Supplement: Supplementary file 1 [file ijms-24-09570-s001.zip › ijms-2351213-supplementary.pdf]

Supplementary Materials

Supplementary Figures

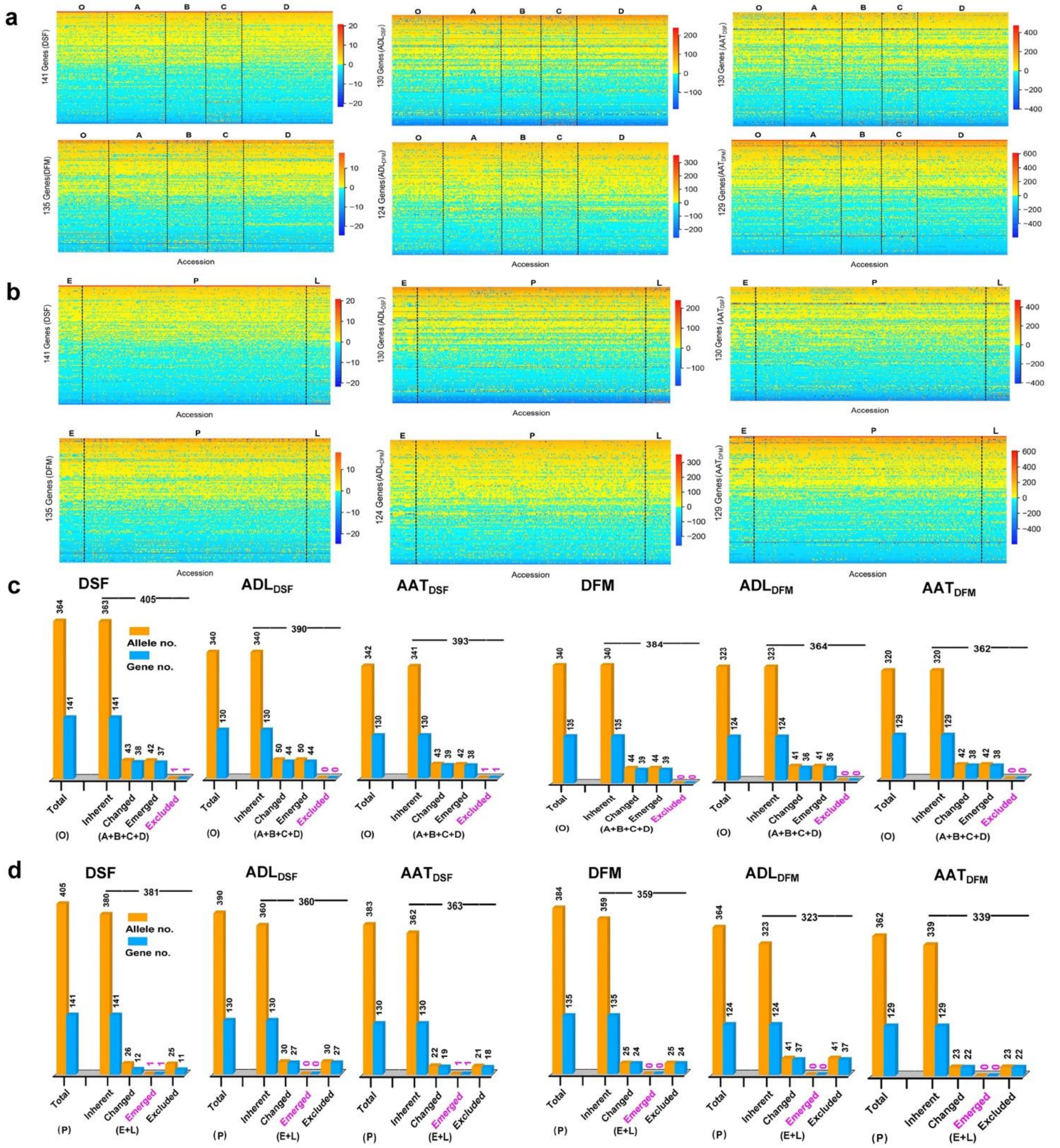

Figure S1. Allelic differentiation of six DSF- and DFM-related traits among geographic and MG-set subpopulations in the WSGP.

(a) The allele matrix of required ADL and AAT for growth period traits among geographic subpopulations in the WSGP; **(b)** The allele matrix of required ADL and AAT for growth period traits among maturity groups in the WSGP; **(c)** The alleles of required ADL and AAT for growth period traits changed in A+B+C+D compared with those in O in the WSGP;

(d) The alleles of required ADL and AAT for growth period traits changed in E+L compared with those in P in the WSGP. Note: DSF: days from sowing-to-flowering; ADL_{DSF}: DSF required accumulative day-length; AAT_{DSF}: DSF required accumulative active temperature; DFM: days from flowering-to-maturity; ADL_{DFM}: DFM required accumulative day-length; AAT_{DFM}: DFM required accumulative active temperature. “O” represents the center of origin; “A” represents Northeast China (NCHN), far-east of Russia (RUFE), and southern Sweden (SSWE); “B” represents the Korea Peninsular (KORP) and Japan Islands (JPAN); “C” represents Southeast Asia (SEAS), South Asia (SASI), and Africa (AFRI); “D” represents northern North America (NNAM), southern North America (SNAM), and Central and South America (CSAM). MG: maturity group; “E” represents the early MG-set (MG 000-0); “P” represents the primary MG-set (MG I-VII), “L” represents the late MG-set (MG VIII-X); A+B+C+D: represents a subpopulation composed of geographic subpopulation materials from A, B, C, and D; E+L: represents a subpopulation composed of geographic subpopulation materials from E and L.

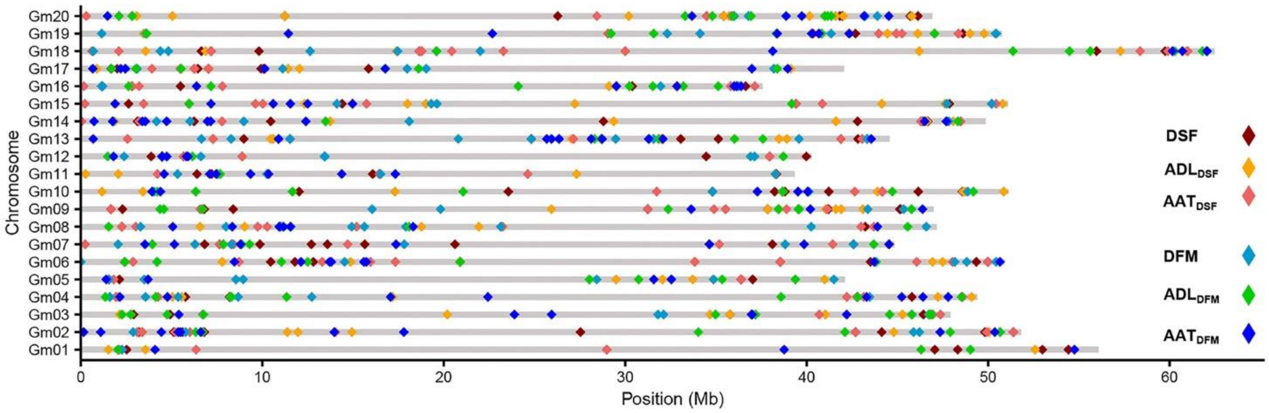

Figure S2. Distribution of all genes associated with six DSF- and DFM-related traits on chromosomes in the WSGP Note: DSF: days from sowing-to-flowering; ADL_{DSF}: DSF required accumulative day-length; AAT_{DSF}: DSF required accumulative active temperature; DFM: days from flowering-to-maturity; ADL_{DFM}: DFM required accumulative day-length; AAT_{DFM}: DFM required accumulative active temperature.

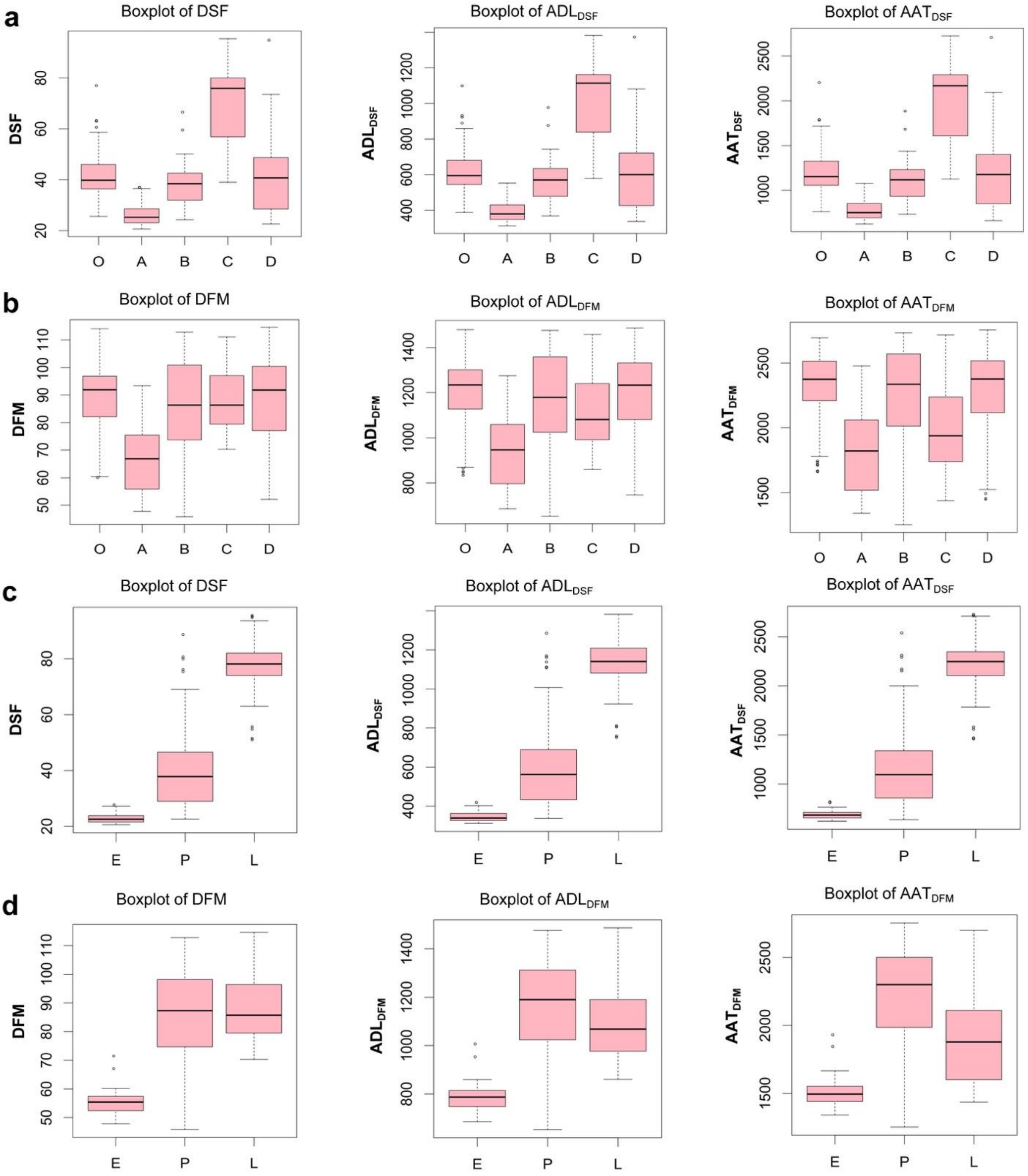

Figure S3. Boxplots of six DSF- and DFM-related traits for geographic subpopulations and MG-sets in the WSGP. Note: DSF: days from sowing-to-flowering; ADL_{DSF}: DSF required accumulative day-length; AAT_{DSF}: DSF required accumulative active temperature; DFM: days from flowering-to-maturity; ADL_{DFM}: DFM required accumulative day-length; AAT_{DFM}: DFM required accumulative active temperature. "O" represents the center of origin; "A" represents Northeast China (NCHN), far-east of Russia (RUFE), and southern Sweden (SSWE); "B" represents the Korea Peninsular (KORP) and Japan Islands (JPAN); "C" represents Southeast Asia (SEAS), South Asia (SASI), and Africa (AFRI); "D" represents northern North America (NNAM), southern North America (SNAM), and Central and South America (CSAM). MG: maturity group; "E" represents the early MG-set (MG 000-0); "P" represents the primary MG-set (MG I-VII); "L"

represents the late MG-set (MG VIII-X).

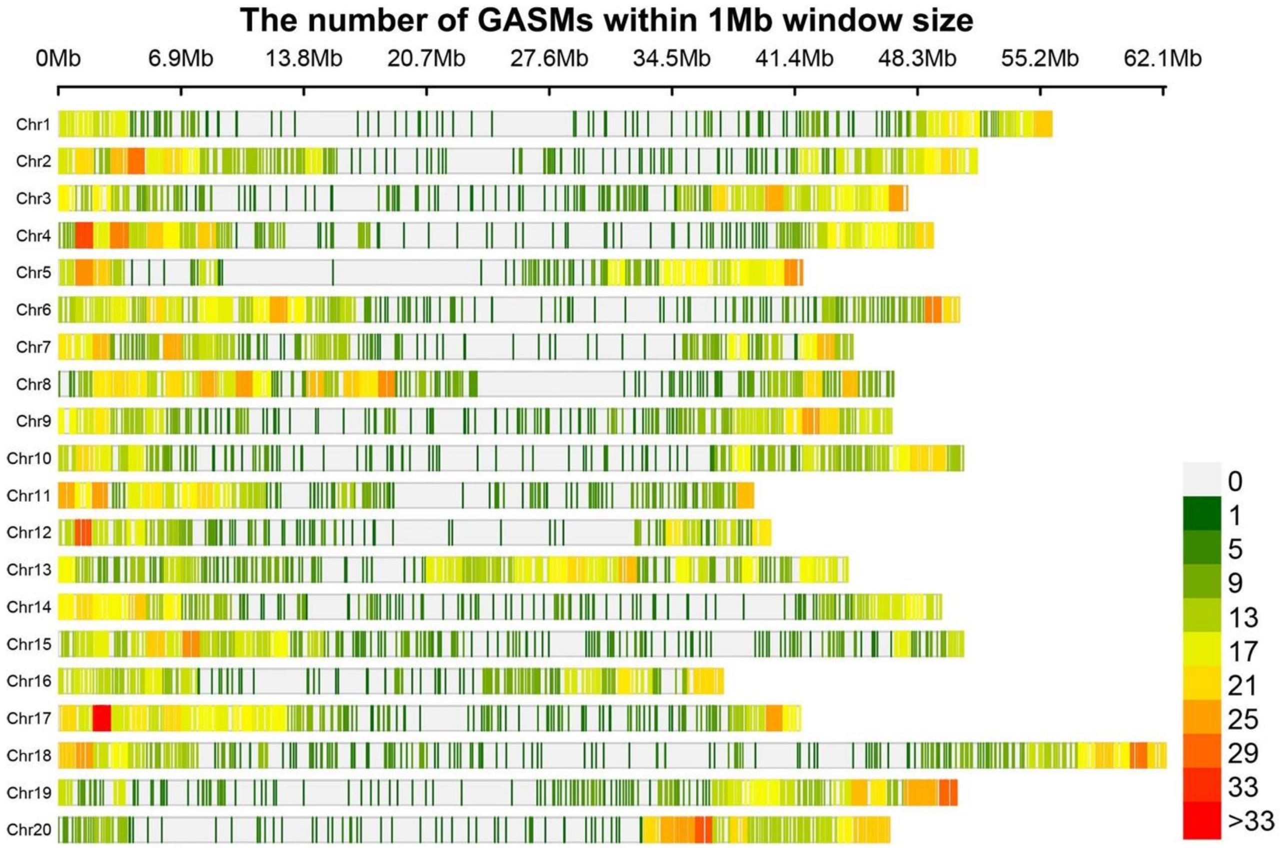

Figure S4. The distribution of GASMs on chromosomes in the WSGP.

Supplementary Tables

Table S1 Variation in six DSF- and DFM-related traits among geographic regions and MG-sets in the WSGP.

Factor	Sub-population	No.	DSF (d) (h^2 = 95.7%)	ADL_{DSF} (d•h) (h^2 = 95.8%)	AAT_{DSF} (d•°C) (h^2 = 99.2%)
			Mean (Range)	Mean (Range)	Mean (Range)
GS	O	65	41.9$^{b\,§}$(25.6-77.0)	621.9^{b} (388.4-1098.8)	1211.1^{b} (763.5-2202.8)
	A	75	26.4^{c} (20.6-37.1)	397.5^{c} (311.7-552.5)	785.4^{c} (622.4-1077.6)
	B	51	38.1^{b} (24.3-66.6)	565.9^{b} (368.6-975.7)	1105.5^{b} (734.5-1883.9)
	C	45	70.6^{a} (39.0-95.5)	1035.4^{a} (579.0-1382.3)	2017.8^{a} (1125.4-2726.2)
	D	117	39.4^{b} (22.6-94.8)	584.9^{b} (336.8-1372.7)	1143.7^{b} (661.3-2708.3)
MG	E	31	23.0^{c} (20.6-27.7)	346.0^{c} (311.7-418.9)	691.3^{c} (622.6-819.1)
	P	292	39.1^{b} (22.6-88.6)	580.5^{b} (336.8-1285.2)	1132.8^{b} (638.2-2539.0)
	L	30	77.5^{a} (51.0-95.5)	1129.6^{a} (751.7-1382.3)	2213.7^{a} (1458.5-2726.2)
Total		353	41.0 (20.6-95.5)	606.6 (311.7-1382.3)	1185.9 (622.6-2726.2)
GS			DFM (d) (h^2=80.7%)	ADL_{DFM}(d•h) (h^2=78.5%)	AAT_{DFM} (d•°C) (h^2=70.9%)
	O	65	88.1^{a} (60.1-114.1)	1191.9^{a} (834.0-1479.4)	2289.7^{a} (1661.1-2694.1)
	A	75	66.5^{b} (47.8-93.4)	936.2^{b} (685.0-1274.8)	1814.7^{c} (1339.8-2477.6)
	B	51	85.1^{a} (45.8-112.8)	1159.1^{a} (652.0-1476.4)	2228.1^{a} (1251.5-2732.9)
	C	45	87.8^{a} (70.3-111.1)	1120.8^{a} (859.5-1458.0)	2016.9^{b} (1437.5-2715.7)
	D	117	88.7^{a} (52.1-114.6)	1200.6^{a} (746.8-1487.4)	2289.8^{a} (1449.1-2755.2)
MG	E	31	55.6 ^{b} (47.8-71.5)	791.5^{b} (685.0-1006.8)	1514.9^{c} (1340.0-1931.0)
	P	292	85.7 ^{a} (45.8-112.8)	1165.0^{a} (652.0-1476.4)	2235.1^{a} (1252.0-2755.0)
	L	30	87.6 ^{a} (70.3-114.6)	1099.7^{a} (859.5-1487.4)	1920.6^{b} (1437.0-2700.0)
Total		353	83.3 (45.8-114.6)	1126.7 (652.0-1487.4)	2145.1 (1251.5-2755.2)

Note: GS: geographic subpopulations; MG: maturity group; DSF: days from sowing-to-flowering; ADL_{DSF}: DSF required accumulative day-length; AAT_{DSF}: DSF required accumulative active temperature; DFM: days from flowering-to-maturity; ADL_{DFM} : DFM required accumulative day-length; AAT_{DFM} : DFM required accumulative active temperature. No.: the number of accessions; h^2: heritability value calculated from ANOVA; "O" represents the center of origin; "A" represents Northeast China (NCHN), far-east of Russia (RUFE), and southern Sweden (SSWE); "B" represents the Korea Peninsular (KORP) and Japan Islands (JPAN); "C" represents Southeast Asia (SEAS), South Asia (SASI), and Africa (AFRI); "D" represents northern North America (NNAM), southern North America (SNAM), and Central and South America (CSAM); "E" represents the early MG-set (MG 000-0); "P" represents the primary MG-set (MG I-VII); "L" represents the late MG-set (MG VIII-X); §: the superscripts of a, b, c, and d indicate differences at $p \leq 0.05$ significance level in multiple comparisons.

Table S2 The gene–allele system conferring DSF identified from GASM-RTM-GWAS in the WSGP.

Gene Code	Genes	Allele no.	Model P -lg(P)	Main-effect		Gene×Env.		Reported Genes/QTLs	Reported in Liu et al. 2021	Gene group
				R^2	-lg(P)	R^2	-lg(P)			
g-DSF-01-1	*Glyma01g03040*	2	31.5	0.85	226.7					⑧
g-DSF-01-2	*Glyma01g22830*	2	61.2	3.05	307.7			*Fflr 8-1*	√	⑤
g-DSF-01-3	*Glyma01g34630*	2	12.1	0.06	20.3					⑧
g-DSF-01-4	*Glyma01g35820*	2	5.5	0.01	4					⑥
g-DSF-01-5	*Glyma01g41520*	2	16	0.2	62.4					⑤/⑦/⑥
g-DSF-01-6	*Glyma01g43400*	4	22	0.55	152.6					⑩
g-DSF-02-1	*Glyma02g03920*	2	30.8	1.25	309.6					⑩
g-DSF-02-2	*Glyma02g04010*	2	5.8	0.01	5.6					⑧
g-DSF-02-3	*Glyma02g04190*	3	41.5	2.98	307.7	0.19	53.6			⑤/⑧
g-DSF-02-4	*Glyma02g06420*	3	8.9	0.03	8.6					⑤/⑧
g-DSF-02-5	*Glyma02g07010*	4	27	0.87	228.6					⑦
g-DSF-02-6	*Glyma02g08790*	2	2.7	4.2E-03	1.9			*GmFT2*c*(421.7Kb)*		②/①
g-DSF-02-7	*Glyma02g26620*	2	6.2	0.03	9.5			*Fflr 13-4*		①
g-DSF-02-8	*Glyma02g37350*	3	27.5	0.63	174.8	0.31	88.7	*Fflr 24-1*		⑦
g-DSF-02-9	*Glyma02g38790*	2	8.4	0.05	15.5			*Fflr 15-1*		⑩
g-DSF-02-10	*Glyma02g45630*	2	6	0.01	4.5					⑥/⑧
g-DSF-02-11	*Glyma02g48010*	2	39.8	2.08	307.7	0.33	95.9			⑩
g-DSF-03-1	*Glyma03g02940*	6	12	0.08	23.4					⑩
g-DSF-03-2	*Glyma03g03120*	5	15.8	0.14	42.2					①
g-DSF-03-3	*Glyma03g04720*	6	15.5	0.22	64.4					⑩
g-DSF-03-4	*Glyma03g27970*	4	15	0.16	48.7				√	③/⑦
g-DSF-03-5	*Glyma03g40780*	3	10	0.07	20.8					③/⑧
g-DSF-03-6	*Glyma03g41290*	2	5.2	0.04	12.3			*LHY2b(693.3Kb)*		⑧
g-DSF-04-1	*Glyma04g07430*	2	6.5	0.02	8.2			*J(ELF3,1738.3Kb)*		⑧
g-DSF-04-2	*Glyma04g09910*	2	11.6	0.11	36.2			*CRY1a(1061.6Kb)*		⑦
g-DSF-04-3	*Glyma04g36240*	3	29.6	1.06	269.5					⑩
g-DSF-04-4	*Glyma04g39610*	2	1.7	0.01	3.2					⑥
g-DSF-05-1	*Glyma05g02140*	3	28.6	0.8	214.2				√	⑩
g-DSF-05-2	*Glyma05g02790*	2	11.3	0.04	12.9				√	⑥
g-DSF-05-3	*Glyma05g31250*	3	17.4	0.19	59.3					③
g-DSF-05-4	*Glyma05g32030*	2	3.2	4.8E-03	2.1					⑧
g-DSF-06-1	*Glyma06g03470*	2	6.8	0.04	12.7				√	⑤
g-DSF-06-2	*Glyma06g13320*	2	10.5	0.05	17.6					⑧
g-DSF-06-3	*Glyma06g15020*	2	2.7	3.8E-03	1.7			*WRKY76(268.4Kb)*		⑥
g-DSF-06-4	*Glyma06g16330*	4	6.8	0.02	4.2					⑩
g-DSF-06-5	*Glyma06g23580*	4	68.5	4.25	307.7			*E1(696.3Kb), Fflr 8-1,9-1,10-1,20-1, 22-2,23-1,26-10,26-11,26-13,26-15,26-16,26-17*	√	⑩
g-DSF-06-6	*Glyma06g36380*	2	38	1.93	307.7			*Fflr 3-1,7-1,8-1,9-1,10-1,18-1, 20-1,22-2,23-1,26-10,26-13,26-16,26-18*	√	④
g-DSF-06-7	*Glyma06g40370*	4	23.8	0.4	115.2			*Fflr 2-1,9-1,10-1,20-1*	√	⑧
g-DSF-06-8	*Glyma06g46781*	3	12.6	0.06	18.9					⑨
g-DSF-06-9	*Glyma06g47590*	4	13.6	0.11	32.6					⑩
g-DSF-07-1	*Glyma07g08214*	2	13.3	0.11	36.7			*Fflr 2-2*		⑧
g-DSF-07-2	*Glyma07g09170*	8	14.5	0.14	37.7			*Fflr 2-2*		⑩
g-DSF-07-3	*Glyma07g09860*	2	2	2.5E-03	1.3			*Fflr 2-2*		⑧
g-DSF-07-4	*Glyma07g11670*	3	30.2	0.79	211.6			*Fflr 2-2*		⑧
g-DSF-07-5	*Glyma07g13710*	2	6.4	0.02	8.6					⑩
g-DSF-07-6	*Glyma07g14242*	2	2.9	0.01	2.4					⑩

g-DSF-07-7	*Glyma07g15930*	4	18.3	0.18	54.1					⑥
g-DSF-07-8	*Glyma07g20421*	2	4.3	0.02	6.1					⑩
g-DSF-07-9	*Glyma07g29650*	2	23.7	0.36	106.8				√	⑨
g-DSF-07-10	*Glyma07g33190*	2	12.3	0.1	32.6				√	⑩
g-DSF-07-11	*Glyma07g37580*	2	22.2	0.36	106.3					⑩
g-DSF-08-1	*Glyma08g03210*	4	66.7	6.07	307.7	0.19	51.3		√	⑨
g-DSF-08-2	*Glyma08g15181*	3	14.1	0.04	12.6					⑩
g-DSF-08-3	*Glyma08g43390*	4	28.4	0.81	214.5				√	⑨
g-DSF-09-1	*Glyma09g02470*	3	40.2	2.81	307.7	0.43	119.7			⑧
g-DSF-09-2	*Glyma09g03270*	3	8.9	0.04	12.9				√	⑥
g-DSF-09-3	*Glyma09g07803*	3	6.4	0.02	6.6			*Fflr 24-2*	√	⑩
g-DSF-09-4	*Glyma09g08940*	2	13.6	0.13	41.1			*Fflr 21-3,24-2*	√	⑩
g-DSF-09-5	*Glyma09g25215*	2	21.1	0.33	98.9			*Fflr 24-2*		④/⑥
g-DSF-09-6	*Glyma09g31087*	2	14.7	0.1	33					⑧
g-DSF-09-7	*Glyma09g34850*	4	32.6	1.79	307.7					⑨
g-DSF-09-8	*Glyma09g34940*	2	6.4	0.03	10					⑧/⑨
g-DSF-09-9	*Glyma09g40181*	2	2.4	4.6E-03	2.1				√	⑦
g-DSF-09-10	*Glyma09g40230*	4	10.2	0.06	19.5				√	⑤/③/⑦
g-DSF-10-1	*Glyma10g11700*	3	4.7	0.02	6.2			*Fflr 13-10*		⑩
g-DSF-10-2	*Glyma10g19040*	2	1.6	3.9E-03	1.8			*Fflr 13-10*		⑩
g-DSF-10-3	*Glyma10g26450*	5	31.7	1.69	307.7			*E4(PhyA,2112.8Kb), Fflr 13-10*		④/⑤/③
g-DSF-10-4	*Glyma10g29380*	2	8.6	0.04	12.1			*E4(PhyA1,1269.7Kb)*		②
g-DSF-10-5	*Glyma10g30100*	3	5.4	0.04	13					⑥
g-DSF-10-6	*Glyma10g32840*	2	17.4	0.1	33.3					⑤
g-DSF-10-7	*Glyma10g35960*	2	31	0.97	253.1			*E2(GmGI,545.6Kb),Fflr 24-4*		⑦
g-DSF-10-8	*Glyma10g38360*	2	3.8	2.7E-03	1.3					⑨
g-DSF-10-9	*Glyma10g41540*	3	13.3	0.1	30.7					⑤
g-DSF-11-1	*Glyma11g09060*	2	15	0.13	40.3					⑦/⑧
g-DSF-11-2	*Glyma11g10100*	3	6.7	0.02	7.9					①/⑧
g-DSF-11-3	*Glyma11g14500*	3	34.2	1.42	307.7	0.62	168.3			⑩
g-DSF-11-4	*Glyma11g19400*	2	1.6	4.7E-03	2.1			*Fflr 8-4*	√	⑩
g-DSF-11-5	*Glyma11g19670*	3	31.1	1.04	266.5	0.19	55.3	*Fflr 8-4*	√	⑧
g-DSF-11-6	*Glyma11g37110*	2	21.3	0.46	133.9					①
g-DSF-12-1	*Glyma12g05780*	4	10	0.05	16.2					⑨
g-DSF-12-2	*Glyma12g08000*	4	29.8	1.59	307.7			*PRR3b(152.2Kb),Fflr 25-2*		⑩
g-DSF-12-3	*Glyma12g30870*	3	10.8	0.05	16.6					⑩
g-DSF-12-4	*Glyma12g34830*	3	20.6	0.36	105.2					⑩
g-DSF-12-5	*Glyma12g36990*	4	21	0.44	124.9					⑩
g-DSF-13-1	*Glyma13g07110*	3	112.1	5.6	307.7					⑦
g-DSF-13-2	*Glyma13g08490*	2	1.9	0.01	3.5				√	⑤
g-DSF-13-3	*Glyma13g09970*	6	21.2	0.43	121.1			*Fflr 25-1*	√	⑩
g-DSF-13-4	*Glyma13g21340*	3	10.6	0.01	2.4					⑧
g-DSF-13-5	*Glyma13g25480*	6	2.7	0.01	2.3				√	④
g-DSF-13-6	*Glyma13g30420*	3	4.7	0.02	6.4					⑩
g-DSF-13-7	*Glyma13g33260*	2	2.6	0.01	4.7					⑧
g-DSF-13-8	*Glyma13g43050*	2	24.6	0.57	162.5					⑤/⑦
g-DSF-14-1	*Glyma14g04550*	2	31.7	0.95	247.8				√	⑤/②/⑥/⑦
g-DSF-14-2	*Glyma14g08220*	3	8	0.03	10.7				√	⑦
g-DSF-14-3	*Glyma14g11910*	2	23.1	0.43	127.1					⑩
g-DSF-14-4	*Glyma14g24140*	3	6.6	0.01	3.5					⑦
g-DSF-14-5	*Glyma14g34340*	3	8.5	0.02	5.7			*Fflr 21-1*		⑩
g-DSF-14-6	*Glyma14g37100*	2	13	0.12	38.9					⑩

g-DSF-14-7	*Glyma14g37330*	3	9.8	0.08	25					⑤/⑥
g-DSF-14-8	*Glyma14g37400*	5	15.6	0.18	53.3					②
g-DSF-14-9	*Glyma14g38570*	5	18.9	0.49	138.5				√	⑩
g-DSF-15-1	*Glyma15g03770*	2	4.4	0.01	3.2					⑩
g-DSF-15-2	*Glyma15g08420*	2	10.1	0.08	26.1				√	③/⑧
g-DSF-15-3	*Glyma15g17910*	2	2.7	0.01	3.4			*Fflr 12-3*	√	⑩
g-DSF-15-4	*Glyma15g18280*	3	15.4	0.2	61.8					⑩
g-DSF-15-5	*Glyma15g34840*	3	45.3	3.05	307.7	0.19	54			②/⑧
g-DSF-15-6	*Glyma15g40860*	2	23	0.33	99.2					③
g-DSF-16-1	*Glyma16g01590*	3	21.3	0.45	130			*LHY1a(335.6Kb)*		⑥/⑧
g-DSF-16-2	*Glyma16g03320*	2	38	2.24	307.7			*GmFT5a(1280.3Kb), LHY1a(1306.3Kb),GmFT 3a(1307.0Kb)*		①/③/⑧
g-DSF-16-3	*Glyma16g06061*	2	2.1	0.01	2.8					⑩
g-DSF-16-4	*Glyma16g26260*	2	4.1	0.02	8			*E9(FT2A,343.7Kb), FT2B(382.5Kb),Fflr 9-3*		⑤
g-DSF-16-5	*Glyma16g28910*	4	5.3	0.01	1.6					⑥
g-DSF-16-6	*Glyma16g33881*	2	20.4	0.36	106.2			*Fflr 13-8*		⑧
g-DSF-17-1	*Glyma17g02580*	2	4.1	0.01	2.2				√	①/⑧
g-DSF-17-2	*Glyma17g03020*	3	15.7	0.17	53.3				√	①/⑧
g-DSF-17-3	*Glyma17g03700*	3	15.2	0.21	63.8				√	①/⑦/⑧
g-DSF-17-4	*Glyma17g08460*	2	78.2	6.95	307.7	1.01	258.3		√	⑩
g-DSF-17-5	*Glyma17g08761*	3	2.8	0.01	1.9				√	⑤/①
g-DSF-17-6	*Glyma17g09500*	3	42.2	2.26	307.7	0.34	96.1		√	⑩
g-DSF-17-7	*Glyma17g13030*	2	2.9	0.01	2.6					⑩
g-DSF-17-8	*Glyma17g13151*	2	13.2	0.15	47.2					⑧
g-DSF-17-9	*Glyma17g13260*	2	4.8	0.02	8.1					⑤
g-DSF-17-10	*Glyma17g18472*	2	2.5	0.02	5.7					⑩
g-DSF-18-1	*Glyma18g07900*	4	5.1	0.02	4.8			*Fflr 9-2*		⑥
g-DSF-18-2	*Glyma18g08410*	5	39.4	1.74	307.7			*Fflr 9-2*		⑥
g-DSF-18-3	*Glyma18g10975*	5	13.8	0.13	38.2				√	⑩
g-DSF-18-4	*Glyma18g17395*	4	35.7	1.55	307.7	0.25	69.7			⑧
g-DSF-18-5	*Glyma18g17515*	3	3.3	0.02	5.1					⑩
g-DSF-18-6	*Glyma18g46220*	4	10.6	0.05	13.9			*SOC1(477.1kb)*	√	⑤/③
g-DSF-18-7	*Glyma18g50725*	3	10.9	0.05	17.4					⑩
g-DSF-19-1	*Glyma19g32600*	3	13.6	0.23	70.7			*Fflr 2-3,4-3,15-2*		⑤/②/①
g-DSF-19-2	*Glyma19g33000*	3	18.8	0.28	84			*Fflr 2-3,4-3,15-2*		⑧
g-DSF-19-3	*Glyma19g35130*	2	1.7	0.01	2.5			*Fflr 2-3,4-3,24-3,25-4*		⑥
g-DSF-19-4	*Glyma19g42710*	2	7.1	0.04	13.8			*E3(GmphyA3,1084.2Kb), LHY2a(1667.9Kb),Fflr 5-3,8-3,20-2*		⑥
g-DSF-20-1	*Glyma20g08091*	4	7.2	0.04	11.8			*Fflr 16-3,20-3*	√	⑥
g-DSF-20-2	*Glyma20g18724*	4	29.8	0.81	214.6			*Fflr 16-3,20-3,25-3*		⑩
g-DSF-20-3	*Glyma20g25790*	4	19.7	0.25	72.9			*Fflr 25-3*	√	⑨
g-DSF-20-4	*Glyma20g33235*	2	28.8	0.68	188					⑩
g-DSF-20-5	*Glyma20g33361*	2	2.4	0.01	3.1					⑦
g-DSF-20-6	*Glyma20g37960*	2	5.5	0.04	15.3				√	⑧
g-DSF-20-7	*Glyma20g38570*	3	7.4	0.02	5.6				√	②/⑦
Total	141	[a] 406(2.88)		76.85		4.05		[b] 63(44)	[c] 39	176

Note: Gene code: for example, *g-DSF-01-1*, where *DSF* means days from sowing-to-flowering, -01 represents chromosome 1, and -1 represents its order on the chromosome according to its physical position. The position corresponds to the Williams 82 reference genome version 1 (Wm82.a1). DSF: days from sowing-to-flowering; Main-effect: main-effect gene; Gene×Env.: gene interaction with environment.

R^2: genetic contribution of a GASM/gene.

Model *P*: the model-based joint probability value in RTM-GWAS (using a threshold of $p \leq 0.05$ or $-$ Log10P ≥ 1.3);
Reported genes/QTLs: the mapped QTLs in this study are consistent with previously reported genes/QTLs in SoyBase (http://www.soybase.org); the QTL name is simplified; for example, *Fflr 8-1* represents first flower *8-1*.
Gene group: Gene Ontology groups (please see the notes of Table S2 for details).
[a]: The number before the parentheses represents the number of total alleles, and the number in the parentheses represents the average number of alleles per gene. The same applies below.
[b]: The number before the parentheses represents the number of QTLs/genes previously reported, and the number in the parentheses represents the number of loci with reported genes or QTLs. The same applies below.
[c]: "√ " indicates that the gene overlaps or is near the QTL discovered in Liu et al. (2021). The same applies below.

Table S3 The gene–allele system conferring DFM identified from GASM-RTM-GWAS in the WSGP.

Gene Code	Genes	Allele no.	Model P -lg(P)	Main-effect		Gene×Env.		Known Genes/QTLs	Reported in Liu's Literature	Gene group
				R2	-lg(P)	R2	-lg(P)			
g-DFM-01-1	*Glyma01g02780*	2	14.1	0.39	53.5				√	⑧
g-DFM-02-1	*Glyma02g00371*	4	77.5	2.66	287.0	7.36	307.7			⑧
g-DFM-02-2	*Glyma02g03670*	2	4.9	0.01	1.8					①/ ⑧/⑨
g-DFM-02-3	*Glyma02g07270*	3	14.2	0.35	46.9					④/②
g-DFM-02-4	*Glyma02g40650*	2	4.2	0.01	2.5					⑤/①/⑦
g-DFM-02-5	*Glyma02g41040*	3	10.0	0.11	14.8					⑦
g-DFM-03-1	*Glyma03g02940*	6	8.6	0.08	9.7					⑩
g-DFM-03-2	*Glyma03g06483*	3	25.0	1.09	135.4					④/⑦/⑥
g-DFM-03-3	*Glyma03g24890*	2	23.5	1.19	148.1					⑩
g-DFM-03-4	*Glyma03g25145*	4	12.8	0.19	25.5					①/⑥
g-DFM-03-5	*Glyma03g39870*	3	8.1	0.06	8.3					⑩
g-DFM-03-6	*Glyma03g41200*	3	13.5	0.11	14.7					⑧
g-DFM-04-1	*Glyma04g02330*	4	10.0	0.14	18.0				√	①
g-DFM-04-2	*Glyma04g04810*	3	5.8	0.04	5.9			*J*(448.9Kb)		⑥
g-DFM-04-3	*Glyma04g06940*	2	10.6	0.18	26.1					⑩
g-DFM-04-4	*Glyma04g10451*	3	13.6	0.12	16.9			*CRY1a*(559.9Kb)		⑥
g-DFM-04-5	*Glyma04g13142*	3	16.7	0.78	100.3					⑩
g-DFM-04-6	*Glyma04g37000*	2	16.1	0.33	45.6					⑦
g-DFM-05-1	*Glyma05g02130*	2	7.3	0.05	7.9					⑧/⑨
g-DFM-05-2	*Glyma05g04330*	3	2.8	0.02	2.4					⑩
g-DFM-05-3	*Glyma05g08670*	3	23.6	0.81	103.6				√	⑩
g-DFM-05-4	*Glyma05g09170*	2	8.1	0.04	6.7				√	⑩
g-DFM-05-5	*Glyma05g23000*	3	10.0	0.11	15.3					⑩
g-DFM-05-6	*Glyma05g26620*	2	6.0	0.05	7.4					④/⑧
g-DFM-05-7	*Glyma05g29200*	2	8.0	0.08	12.1			*FLC*(848.7Kb)		③/⑦/⑧
g-DFM-05-8	*Glyma05g38075*	3	18.7	0.94	118.4					⑩
g-DFM-06-1	*Glyma0006s50*	2	1.7	0.01	2.2					④/⑦
g-DFM-06-2	*Glyma06g05870*	3	29.9	1.05	130.9	1.34	158.2			⑩
g-DFM-06-3	*Glyma06g18216*	4	14.8	0.55	70.8					④/①
g-DFM-06-4	*Glyma06g40670*	4	14.1	0.49	63.6					⑧

g-DFM-06-5	*Glyma06g45450*	4	20.1	0.72	92.1				√	⑦
g-DFM-06-6	*Glyma06g46150*	3	16.5	0.65	84.6				√	⑥
g-DFM-06-7	*Glyma06g48150*	3	23.8	0.61	79.0				√	⑤
g-DFM-07-1	*Glyma07g03005*	2	7.9	0.1	15.4			*Rsl 2-1*		⑩
g-DFM-07-2	*Glyma07g07600*	2	4.3	0.01	1.3			*Rsl 5-4*	√	⑧
g-DFM-07-3	*Glyma07g09420*	5	22.1	1.44	170.6					⑧
g-DFM-07-4	*Glyma07g09860*	2	3.5	0.01	1.8					⑧
g-DFM-07-5	*Glyma07g18060*	2	5.1	0.05	7.4					⑤/⑧
g-DFM-07-6	*Glyma07g33880*	2	3.5	0.03	5.5					⑦
g-DFM-07-7	*Glyma07g37580*	2	23.4	0.98	124.7				√	⑩
g-DFM-07-8	*Glyma07g40260*	2	22.5	0.69	90.4				√	⑩
g-DFM-08-1	*Glyma08g04620*	2	3.9	0.05	7.7				√	⑤/⑧
g-DFM-08-2	*Glyma08g10960*	2	2.6	0.02	3.4					⑩
g-DFM-08-3	*Glyma08g15250*	3	17.1	0.66	85.3					⑥
g-DFM-08-4	*Glyma08g15870*	2	28.1	1.21	150.0					⑩
g-DFM-08-5	*Glyma08g19780*	2	5.0	0.01	1.6					⑩
g-DFM-08-6	*Glyma08g20625*	2	13.2	0.28	39.2			*Rp 1-5*		⑧
g-DFM-08-7	*Glyma08g23480*	2	18.1	0.73	94.9					⑨
g-DFM-08-8	*Glyma08g28765*	2	2.7	0.01	2.4			*COL2a(495.7Kb)*		⑩
g-DFM-08-9	*Glyma08g40530*	2	16.0	0.29	40.9					⑥
g-DFM-08-10	*Glyma08g47790*	3	7.8	0.09	12.0			*E10(11.4Kb),FT6(871.7K*		⑨
g-DFM-09-1	*Glyma09g14100*	2	5.7	0.03	4.7					④/⑤/①
g-DFM-09-2	*Glyma09g16566*	2	20.1	0.34	47.4				√	①
g-DFM-09-3	*Glyma09g37840*	3	4.1	0.04	4.9				√	⑦/⑧
g-DFM-09-4	*Glyma09g40420*	2	30.7	2.02	232.3				√	⑩
g-DFM-10-1	*Glyma10g05240*	2	3.7	0.04	6.0					⑩
g-DFM-10-2	*Glyma10g26450*	5	7.0	0.09	10.5					④/⑤/②/③
g-DFM-10-3	*Glyma10g29970*	4	17.9	0.76	95.8			*PhyA1(1707.7Kb)*	√	①/⑥
g-DFM-10-4	*Glyma10g31630*	2	43.6	1.91	222.0	2.2	246.0		√	⑧
g-DFM-10-5	*Glyma10g41880*	2	1.3	0.01	1.3					⑧
g-DFM-11-1	*Glyma11g07620*	4	24.5	0.66	84.7					⑦
g-DFM-11-2	*Glyma11g10800*	4	8.9	0.15	19.7					⑩
g-DFM-11-3	*Glyma11g13111*	2	3.5	0.01	1.4				√	⑩
g-DFM-11-4	*Glyma11g14300*	2	3.5	0.02	2.8				√	⑩
g-DFM-11-5	*Glyma11g19735*	2	17.0	0.54	72.6			*Rsl 8-1*	√	⑩
g-DFM-11-6	*Glyma11g37040*	2	1.8	0.01	2.3			*ELF4A(1308.9Kb)*		⑩
g-DFM-12-1	*Glyma12g03580*	3	12.2	0.24	32.5					⑥
g-DFM-12-2	*Glyma12g06580*	3	18.1	0.77	98.4					⑧
g-DFM-12-3	*Glyma12g06620*	2	14.0	0.21	29.8					⑧
g-DFM-12-4	*Glyma12g06950*	2	12.2	0.06	8.9					①
g-DFM-12-5	*Glyma12g08860*	2	2.1	0.02	2.9			*Rsl 7-3*		①
g-DFM-12-6	*Glyma12g14508*	3	1.9	0.02	2.7				√	⑧
g-DFM-12-7	*Glyma12g14530*	2	3.3	0.01	2.2				√	⑥
g-DFM-12-8	*Glyma12g33700*	2	13.2	0.27	38.1					⑩
g-DFM-12-9	*Glyma12g33940*	2	15.4	0.43	58.3					⑩
g-DFM-13-1	*Glyma13g06400*	4	5.3	0.04	4.6					⑩
g-DFM-13-2	*Glyma13g08035*	3	16.9	0.3	40.4					⑩
g-DFM-13-3	*Glyma13g09470*	5	54.3	1.25	150.7	5.09	307.7			⑩
g-DFM-13-4	*Glyma13g09970*	6	15.1	0.52	65.1					⑩
g-DFM-13-5	*Glyma13g16940*	3	13.6	0.25	33.3					⑦
g-DFM-13-6	*Glyma13g21340*	3	8.0	0.06	8.6					⑧

g-DFM-13-7	*Glyma13g22870*	3	10.1	0.02	2.6					⑩
g-DFM-13-8	*Glyma13g25480*	6	42.0	2.59	278.7	2.12	226.2	*Rsl 7-2*	√	④
g-DFM-13-9	*Glyma13g26270*	4	10.3	0.14	17.7				√	⑧
g-DFM-13-10	*Glyma13g28280*	4	16.3	0.76	95.9				√	⑧
g-DFM-13-11	*Glyma13g28570*	2	8.9	0.01	1.9					⑧
g-DFM-13-12	*Glyma13g28880*	4	10.2	0.17	22.4					⑤
g-DFM-13-13	*Glyma13g38860*	2	8.6	0.14	20.4					⑧
g-DFM-13-14	*Glyma13g43710*	2	12.7	0.12	17.0					⑩
g-DFM-13-15	*Glyma13g43740*	3	26.5	1.59	188.7					④/⑤
g-DFM-14-1	*Glyma14g02790*	4	18.5	0.46	60.1					⑥
g-DFM-14-2	*Glyma14g05780*	3	13.2	0.27	36.2				√	⑩
g-DFM-14-3	*Glyma14g07960*	2	9.1	0.06	8.6					⑧
g-DFM-14-4	*Glyma14g10790*	2	8.6	0.11	15.8					⑧
g-DFM-14-5	*Glyma14g16655*	2	11.6	0.12	17.2					⑩
g-DFM-14-6	*Glyma14g38670*	3	4.3	0.02	2.4				√	⑧
g-DFM-15-1	*Glyma15g17710*	2	10.4	0.09	13.8					⑧
g-DFM-15-2	*Glyma15g21101*	5	7.2	9.0E-05	5.2					⑦
g-DFM-15-3	*Glyma15g21400*	2	16.9	0.43	58.4					⑩
g-DFM-15-4	*Glyma15g40740*	4	11.5	0.07	8.6					⑧
g-DFM-15-5	*Glyma15g42762*	4	8.4	0.09	12.0					⑩
g-DFM-16-1	*Glyma16g01570*	2	6.1	0.02	4.2				√	⑩
g-DFM-16-2	*Glyma16g01640*	2	7.7	0.06	8.8			*FT5A*(2903.4Kb),*FT3A*(2	√	①
g-DFM-16-3	*Glyma16g25500*	2	32.8	2.12	241.7			*E9*(1225.6Kb),*FT2B*(126		⑩
g-DFM-16-4	*Glyma16g28010*	6	4.0	0.06	5.9				√	⑩
g-DFM-16-5	*Glyma16g33320*	4	11.4	0.19	25.2			*TFL1.3*(998.3Kb)		①
g-DFM-17-1	*Glyma17g04661*	2	13.5	0.17	24.5				√	⑩
g-DFM-17-2	*Glyma17g14370*	2	2.5	0.01	2.4			*GmYABBY16*(318.8Kb)		⑤
g-DFM-17-3	*Glyma17g19660*	3	3.2	0.03	4.2					⑧
g-DFM-17-4	*Glyma17g20310*	3	12.8	0.1	14.2			*Rp1-7*		⑩
g-DFM-17-5	*Glyma17g34321*	4	7.1	1.6E-04	10.0				√	⑧
g-DFM-18-1	*Glyma18g01330*	2	3.1	0.01	2.5			*ELF4B*(1368.1Kb)	√	③/⑨
g-DFM-18-2	*Glyma18g05730*	2	6.0	0.05	8.0					⑧
g-DFM-18-3	*Glyma18g06250*	2	8.2	0.01	1.4					⑩
g-DFM-18-4	*Glyma18g13175*	2	2.6	0.02	2.7					⑩
g-DFM-18-5	*Glyma18g16780*	2	5.0	0.02	3.6					⑥
g-DFM-18-6	*Glyma18g18220*	3	13.5	0.2	27.0					⑩
g-DFM-18-7	*Glyma18g20146*	5	18.3	0.88	109.7				√	⑩
g-DFM-18-8	*Glyma18g53823*	5	12.1	0.1	12.3			*FT1B*(116Kb),*FT1A*(129.		④/⑥
g-DFM-19-1	*Glyma19g01536*	2	8.0	1.1E-03	1.5				√	⑩
g-DFM-19-2	*Glyma19g03563*	3	4.3	0.03	3.7					①
g-DFM-19-3	*Glyma19g23740*	2	21.9	1.1	138.5					⑩
g-DFM-19-4	*Glyma19g25980*	3	16.1	0.3	39.9					⑩
g-DFM-19-5	*Glyma19g26950*	4	10.9	0.07	8.5			*FT3B*(1722.5Kb),*FT5B*(1 741.0Kb)		⑤/⑧
g-DFM-19-6	*Glyma19g30690*	3	70.5	7.2	307.7					⑩
g-DFM-19-7	*Glyma19g33210*	3	11.0	0.05	6.7					⑥
g-DFM-19-8	*Glyma19g33760*	3	9.2	0.07	9.4					⑩
g-DFM-19-9	*Glyma19g34740*	2	35.9	2.31	259.5			*DT1*(*TFL1b*,2633.3Kb),*R*		⑤/⑧
g-DFM-19-10	*Glyma19g45170*	2	6.2	0.02	3.5			*Rsl 3-3*		⑩
g-DFM-19-11	*Glyma19g45260*	3	15.9	0.39	52.6					⑥

g-DFM-20-1	*Glyma20g03100*	5	15.0	0.24	30.8				⑩
g-DFM-20-2	*Glyma20g26640*	2	8.0	0.07	10.9				⑩
g-DFM-20-3	*Glyma20g27700*	2	17.5	0.07	10.1		*GIGANTEA*(2838.9Kb)		⑥/⑧
g-DFM-20-4	*Glyma20g34910*	2	2.9	0.01	2.2				①
g-DFM-20-5	*Glyma20g35670*	4	4.4	0.05	5.9				⑤
Total	135	384(2.84)		55.05		18.11	28(23)	34	99

Note: Gene code: for example, *g-DFM-01-1*, where *DFM* means days from flowering-to-maturity, -01 represents chromosome 1, and -1 represents its order on the chromosome according to its physical position. The position corresponds to the Williams 82 reference genome version 1 (Wm82.a1). Reported QTL, the QTL name is simplified; for example, *Rp1-3* represents reproductive period *1-3* and *Rsl7-1* represents reproductive stage length *7-1*. The same applies below. Gene group: Gene Ontology groups (please see the notes of Table S2 for details).

Table S4 The gene–allele system conferring ADL$_{DSF}$ identified from GASM-RTM-GWAS in the WSGP.

Gene Code	Genes	Allele no.	Model P -lg(P)	Main-effect		Gene×Env.		Known Genes/QTLs	Gene group
				R^2	-	R^2	-lg(P)		
g-ADL$_{DSF}$-01-1	*Glyma01g01950*	2	18.6	0.24	87.3				⑩
g-ADL$_{DSF}$-01-2	*Glyma01g04100*	2	15.1	0.18	67.1				⑨
g-ADL$_{DSF}$-01-3	*Glyma01g22830*	2	61.0	2.9	307.7	1.86	307.7	*Fflr 16-1*	⑤
g-ADL$_{DSF}$-01-4	*Glyma01g40930*	2	3.8	3.8E-03	2				⑥
g-ADL$_{DSF}$-02-1	*Glyma02g00371*	4	31.4	1.27	307.7				⑧
g-ADL$_{DSF}$-02-2	*Glyma02g04190*	3	33.1	1.39	307.7				⑤/⑧
g-ADL$_{DSF}$-02-3	*Glyma02g06730*	4	29.1	1.00	290.7			*GmFT2c(659.9Kb)*	⑤/①
g-ADL$_{DSF}$-02-4	*Glyma02g08620*	3	32.0	0.75	232				⑩
g-ADL$_{DSF}$-02-5	*Glyma02g13165*	5	9.9	0.06	21.3			*Fflr 16-2*	⑩
g-ADL$_{DSF}$-02-6	*Glyma02g13655*	2	19.7	0.28	97.6			*Fflr 16-2*	⑧
g-ADL$_{DSF}$-02-7	*Glyma02g16560*	2	7.3	0.01	6.2			*Fflr 16-2*	⑩
g-ADL$_{DSF}$-02-8	*Glyma02g39495*	3	8.7	0.01	2.4			*Fflr 15-1*	④/⑧
g-ADL$_{DSF}$-02-9	*Glyma02g45960*	2	23.8	0.65	207.7				⑤
g-ADL$_{DSF}$-03-1	*Glyma03g02390*	2	8.4	0.02	9.2				⑥
g-ADL$_{DSF}$-03-2	*Glyma03g15870*	2	13.3	0.06	24.5				⑤
g-ADL$_{DSF}$-03-3	*Glyma03g27150*	3	14.8	0.08	31.2				⑩
g-ADL$_{DSF}$-03-4	*Glyma03g27970*	4	24.5	0.4	134.1				③/⑦
g-ADL$_{DSF}$-03-5	*Glyma03g29070*	3	20.9	0.38	129.8				⑤
g-ADL$_{DSF}$-03-6	*Glyma03g33470*	2	3.2	0.02	6.5			*Dt1(TFL1A,1503.6Kb)*	⑤/①
g-ADL$_{DSF}$-03-7	*Glyma03g39050*	2	29.6	1.28	307.7				⑩
g-ADL$_{DSF}$-03-8	*Glyma03g41320*	2	3.7	0.02	6.8			*LHY2b(669.4Kb)*	⑧
g-ADL$_{DSF}$-04-1	*Glyma04g06600*	4	36.4	2.22	307.7			*J(GmELF3,1017.8Kb)*	⑩
g-ADL$_{DSF}$-04-2	*Glyma04g07160*	2	12.5	0.06	21.9				⑤/⑧
g-ADL$_{DSF}$-04-3	*Glyma04g16180*	4	10.3	0.06	19.5				⑤/②
g-ADL$_{DSF}$-04-4	*Glyma04g41390*	2	48.1	2.82	307.7			*Fflr 22-1*	④/⑤/⑥
g-ADL$_{DSF}$-04-5	*Glyma04g43550*	2	2.3	3.0E-03	1.7				⑥
g-ADL$_{DSF}$-05-1	*Glyma05g23731*	3	26.7	1.23	307.7				⑧
g-ADL$_{DSF}$-05-2	*Glyma05g26050*	2	30.1	0.9	269.9				⑩
g-ADL$_{DSF}$-05-3	*Glyma05g27870*	2	32.1	1.32	307.7			*FLC(279.9Kb)*	⑥/⑧
g-ADL$_{DSF}$-05-4	*Glyma05g29190*	5	4.4	0.01	4				⑥/⑧
g-ADL$_{DSF}$-05-5	*Glyma05g37380*	2	2.0	0.01	3.8				⑧
g-ADL$_{DSF}$-06-1	*Glyma06g10320*	5	14.1	0.06	19.9				⑦
g-ADL$_{DSF}$-06-2	*Glyma06g18580*	2	25.1	0.69	216.8				①

g-ADL$_{DSF}$*-06-3*	*Glyma06g23580*	4	72.5	4.24	307.7			*(696.3Kb),Fflr 8-1,9-1,10-1,20-1, 22-*	⑩
g-ADL$_{DSF}$*-06-4*	*Glyma06g32870*	4	13.3	0.08	28.7			*Fflr 3-1,7-1,8-1,9-1,10-1,18-1,20-1, 22-*	⑩
g-ADL$_{DSF}$*-06-5*	*Glyma06g43951*	2	33.1	1.61	307.7			*Fflr 1-1,1-2,9-1,10-1*	①/⑧
g-ADL$_{DSF}$*-06-6*	*Glyma06g44630*	2	19.2	0.37	126.6			*Fflr 1-1,1-2,9-1,10-1*	⑤
g-ADL$_{DSF}$*-07-1*	*Glyma07g05290*	2	32.0	1.65	307.7			*LHY1b(127.8Kb)*	⑩
g-ADL$_{DSF}$*-08-1*	*Glyma08g03210*	4	67.3	6.04	307.7				⑨
g-ADL$_{DSF}$*-08-2*	*Glyma08g09191*	2	20.7	0.44	149.4				⑩
g-ADL$_{DSF}$*-08-3*	*Glyma08g12350*	3	6.2	0.01	3.8			*Fflr 13-1*	⑥/⑧
g-ADL$_{DSF}$*-08-4*	*Glyma08g24630*	3	16.2	0.24	82.9				⑩
g-ADL$_{DSF}$*-08-5*	*Glyma08g27633*	4	33.1	1.78	307.7				⑦
g-ADL$_{DSF}$*-08-6*	*Glyma08g28920*	3	11.6	0.08	28.5				⑤/⑦
g-ADL$_{DSF}$*-09-1*	*Glyma09g21070*	3	7.2	0.02	6			*Fflr 24-2,3-4*	⑥
g-ADL$_{DSF}$*-09-2*	*Glyma09g28620*	4	13.7	0.07	23.5				⑥
g-ADL$_{DSF}$*-09-3*	*Glyma09g31087*	2	23.0	0.37	128.1				⑧
g-ADL$_{DSF}$*-09-4*	*Glyma09g31880*	2	29.5	0.8	246.6				⑤/⑦
g-ADL$_{DSF}$*-09-5*	*Glyma09g34750*	3	14.4	0.08	28.4				⑦
g-ADL$_{DSF}$*-09-6*	*Glyma09g35651*	2	3.9	0.01	5.6				⑩
g-ADL$_{DSF}$*-09-7*	*Glyma09g36030*	3	19.8	0.3	103				⑩
g-ADL$_{DSF}$*-09-8*	*Glyma09g37580*	2	3.1	0.01	2.9				⑧
g-ADL$_{DSF}$*-10-1*	*Glyma10g01610*	4	14.0	0.17	61				②
g-ADL$_{DSF}$*-10-2*	*Glyma10g04440*	2	2.9	0.01	3.1				⑩
g-ADL$_{DSF}$*-10-3*	*Glyma10g14916*	3	14.6	0.12	43.8			*Fflr 13-10*	①
g-ADL$_{DSF}$*-10-4*	*Glyma10g26450*	5	17.0	0.17	59.8			*E4(PhyA,2112.8Kb),Fflr 13-10*	I/⑤/②/③
g-ADL$_{DSF}$*-10-5*	*Glyma10g29970*	4	16.1	0.38	128.6			*E4(PHYA,1707.7Kb)*	⑩
g-ADL$_{DSF}$*-10-6*	*Glyma10g30930*	3	34.1	2.01	307.7				⑩
g-ADL$_{DSF}$*-10-7*	*Glyma10g31570*	3	2.9	0.01	3.6				⑤
g-ADL$_{DSF}$*-10-8*	*Glyma10g35640*	3	15.4	0.16	57.3			*E2(GmGI,810.9Kb)*	⑩
g-ADL$_{DSF}$*-10-9*	*Glyma10g41573*	2	17.8	0.24	85.7				⑥
g-ADL$_{DSF}$*-10-10*	*Glyma10g41710*	3	30.3	0.89	266.1				⑩
g-ADL$_{DSF}$*-10-11*	*Glyma10g44540*	2	31.1	0.99	292				⑧/⑨
g-ADL$_{DSF}$*-11-1*	*Glyma11g00600*	3	4.0	3.5E-03	1.3				⑤
g-ADL$_{DSF}$*-11-2*	*Glyma11g03130*	2	16.3	0.14	53.2				⑩
g-ADL$_{DSF}$*-11-3*	*Glyma11g27510*	3	16.2	0.11	41.7				⑤
g-ADL$_{DSF}$*-11-4*	*Glyma11g37010*	4	11.0	0.02	7.9			*ELF4A(1295.5Kb)*	⑧
g-ADL$_{DSF}$*-11-5*	*Glyma11g37040*	2	2.2	2.9E-03	1.6				⑩
g-ADL$_{DSF}$*-12-1*	*Glyma12g06580*	3	13.2	0.08	28.7				⑧
g-ADL$_{DSF}$*-12-2*	*Glyma12g06620*	2	11.1	0.09	33.4			*PRR3b(993.8Kb)*	⑧
g-ADL$_{DSF}$*-12-3*	*Glyma12g10890*	3	6.4	0.03	10.3				⑤
g-ADL$_{DSF}$*-13-1*	*Glyma13g07110*	3	119.7	5.67	307.7				⑦
g-ADL$_{DSF}$*-13-2*	*Glyma13g09341*	2	48.7	2.19	307.7	0.45	148.8		⑧
g-ADL$_{DSF}$*-13-3*	*Glyma13g09380*	7	22.2	0.49	156.1				⑩
g-ADL$_{DSF}$*-13-4*	*Glyma13g22420*	2	12.6	0.13	47.3				④/⑤/①
g-ADL$_{DSF}$*-13-5*	*Glyma13g23700*	4	18.9	0.48	157.2				⑤/①
g-ADL$_{DSF}$*-13-6*	*Glyma13g28260*	3	5.5	0.02	6.3				⑩
g-ADL$_{DSF}$*-13-7*	*Glyma13g37360*	3	2.8	0.01	2.6				⑩
g-ADL$_{DSF}$*-13-8*	*Glyma13g37980*	5	18.6	0.3	100.4				⑧
g-ADL$_{DSF}$*-13-9*	*Glyma13g41660*	3	8.9	0.02	8.7				②
g-ADL$_{DSF}$*-14-1*	*Glyma14g02780*	3	19.9	0.33	111.6				⑤
g-ADL$_{DSF}$*-14-2*	*Glyma14g02970*	3	12.3	0.06	21.6				③
g-ADL$_{DSF}$*-14-3*	*Glyma14g14000*	3	13.4	0.07	26.2				⑤/⑧
g-ADL$_{DSF}$*-14-4*	*Glyma14g24480*	5	27.5	0.71	218.6				④/⑤/①

g-ADL$_{DSF}$-14-5	*Glyma14g33601*	2	14.6	0.03	13.4			*Fflr 21-1*	⑥
g-ADL$_{DSF}$-14-6	*Glyma14g37330*	3	18.0	0.25	87.9				⑤/⑥
g-ADL$_{DSF}$-14-7	*Glyma14g38910*	4	13.7	0.06	23.1				⑩
g-ADL$_{DSF}$-15-1	*Glyma15g16000*	2	2.0	2.8E-03	1.6			*Fflr 12-3*	⑩
g-ADL$_{DSF}$-15-2	*Glyma15g20200*	2	12.0	0.1	37.4				①
g-ADL$_{DSF}$-15-3	*Glyma15g20935*	2	24.5	0.67	212.4				④/⑤/①
g-ADL$_{DSF}$-15-4	*Glyma15g25690*	6	19.8	0.52	166.3				②/⑦
g-ADL$_{DSF}$-15-5	*Glyma15g38060*	3	13.4	0.13	46.6				③/⑦
g-ADL$_{DSF}$-15-6	*Glyma15g40635*	3	3.4	0.01	2.4				⑩
g-ADL$_{DSF}$-15-7	*Glyma15g43289*	3	32.4	0.99	290.8				⑧
g-ADL$_{DSF}$-16-1	*Glyma16g03320*	2	41.4	2.7	307.7			*GmFT5a（1280.3Kb), LHY1a(1306.3Kb),GmFT3a(1307.0Kb)*	①/③/⑧
g-ADL$_{DSF}$-16-2	*Glyma16g25185*	5	14.3	0.05	18.4			*E9(1621.3Kb),FT2B(1660.1Kb),Fflr 9-3*	⑩
g-ADL$_{DSF}$-16-3	*Glyma16g33100*	4	5.6	0.03	10.8			*Fflr 13-8*	⑧
g-ADL$_{DSF}$-16-4	*Glyma16g33480*	3	13.4	0.08	28.9			*Fflr 13-8*	⑩
g-ADL$_{DSF}$-17-1	*Glyma17g01430*	2	13.8	0.03	13.2				⑤
g-ADL$_{DSF}$-17-2	*Glyma17g04580*	3	27.0	1.3	307.7				⑩
g-ADL$_{DSF}$-17-3	*Glyma17g08460*	2	77.5	6.77	307.7	1.1	312.2		⑩
g-ADL$_{DSF}$-17-4	*Glyma17g09500*	3	40.6	2.72	307.7	0.52	164.7		⑩
g-ADL$_{DSF}$-17-5	*Glyma17g14700*	2	5.1	3.1E-03	1.7				⑤
g-ADL$_{DSF}$-17-6	*Glyma17g15350*	2	17.1	0.2	73.7				⑩
g-ADL$_{DSF}$-17-7	*Glyma17g35130*	2	15.2	0.02	7				⑩
g-ADL$_{DSF}$-18-1	*Glyma18g04820*	3	10.9	0.01	4.2				①
g-ADL$_{DSF}$-18-2	*Glyma18g08180*	3	19.9	0.28	98.6			*Fflr 9-2*	⑩
g-ADL$_{DSF}$-18-3	*Glyma18g16761*	4	13.6	0.06	22.7				⑧
g-ADL$_{DSF}$-18-4	*Glyma18g26120*	2	3.4	3.7E-03	1.9			*Fflr 10-2*	⑧
g-ADL$_{DSF}$-18-5	*Glyma18g38570*	3	4.9	0.01	3			*Fflr 10-2*	⑧
g-ADL$_{DSF}$-18-6	*Glyma18g47720*	2	4.1	0.01	2.5			*SOC1(1800.6Kb)*	⑧
g-ADL$_{DSF}$-18-7	*Glyma18g52250*	6	9.4	0.05	16.5			*FT1A(1033.0Kb),FT1B(1049.8Kb)*	⑩
g-ADL$_{DSF}$-19-1	*Glyma19g03440*	4	16.3	0.2	68.3				③/⑧
g-ADL$_{DSF}$-19-2	*Glyma19g03590*	4	19.1	0.34	116.4				⑤
g-ADL$_{DSF}$-19-3	*Glyma19g23640*	4	13.5	0.07	25.7				⑤/②
g-ADL$_{DSF}$-19-4	*Glyma19g37230*	4	19.0	0.19	65.6			*Fflr 2-3,22-3,24-3*	⑩
g-ADL$_{DSF}$-19-5	*Glyma19g39460*	2	1.7	4.6E-03	2.3			*E3(GmphyA3,1383.5Kb),Fflr 3-3,13-9*	⑤
g-ADL$_{DSF}$-19-6	*Glyma19g43320*	2	17.7	0.15	55.7			*Fflr 5-3,8-3,20-2*	⑧
g-ADL$_{DSF}$-19-7	*Glyma19g44310*	3	14.2	0.06	22.2			*LHY2a(495.4Kb),Fflr 5-3,8-3*	②/⑦
g-ADL$_{DSF}$-20-1	*Glyma20g03330*	3	8.5	0.02	6				⑩
g-ADL$_{DSF}$-20-2	*Glyma20g04761*	2	11.4	0.05	21.3			*Fflr 16-3,20-3*	⑤/⑦
g-ADL$_{DSF}$-20-3	*Glyma20g08091*	4	10.5	0.07	24.4			*Fflr 16-3,20-3*	⑥
g-ADL$_{DSF}$-20-4	*Glyma20g21151*	3	11.8	0.02	6.3			*E4(GmphyA2,1894.1Kb),Fflr 16-3,20-*	⑥
g-ADL$_{DSF}$-20-5	*Glyma20g25800*	4	20.8	0.27	93.7			*Fflr 25-3*	⑩
g-ADL$_{DSF}$-20-6	*Glyma20g26220*	5	12.7	0.1	35.5			*Fflr 25-3*	②/①/⑧
g-ADL$_{DSF}$-20-7	*Glyma20g31551*	2	14.4	0.1	37.7				⑤/⑦
g-ADL$_{DSF}$-20-8	*Glyma20g32390*	3	19.0	0.39	131.9				⑨
g-ADL$_{DSF}$-20-9	*Glyma20g32980*	4	16.9	0.24	83.9				①/⑧
g-ADL$_{DSF}$-20-10	*Glyma20g33430*	4	28.6	0.83	250.3				⑤
g-ADL$_{DSF}$-20-11	*Glyma20g38090*	3	20.0	0.46	151.7				⑩
Total	130	390(3.00)		75.35		3.93		67(44)	170

Note: Gene code: for example, *g-ADL$_{DSF}$-01-1*, where ADL$_{DSF}$ means DSF required accumulative day-length, -01

represents chromosome 1, and -1 represents its order on the chromosome according to its physical position. The position corresponds to the Williams 82 reference genome version 1 (Wm82.a1). Gene group: Gene Ontology groups (please see the notes of Table S2 for details).

Table S5 The gene–allele system conferring AAT$_{DSF}$ identified from GASM-RTM-GWAS in the WSGP.

Gene Code	Genes	Allele no.	Model P -lg(P)	Main-effect		Gene×Env.		Known Genes/QTLs	Gene group
				R2	-lg(P)	R2	-lg(P)		
g-AAT$_{DSF}$*-01-1*	*Glyma01g02580*	2	5.4	0.02	7.9				⑥
g-AAT$_{DSF}$*-01-2*	*Glyma01g06270*	3	19.3	3.2E-03	116.2				⑩
g-AAT$_{DSF}$*-01-3*	*Glyma01g22830*	2	69	4.26	307.7			*Fflr 16-1*	⑤
g-AAT$_{DSF}$*-02-1*	*Glyma02g03920*	2	40.3	1.6	307.7				⑩
g-AAT$_{DSF}$*-02-2*	*Glyma02g04190*	3	43.7	3.12	307.7	0.11	28.8		⑤/⑧
g-AAT$_{DSF}$*-02-3*	*Glyma02g06530*	2	12.8	0.09	27.2				⑤
g-AAT$_{DSF}$*-02-4*	*Glyma02g07640*	2	23.4	0.7	182			*GmFT2c*(11.4Kb)	⑧
g-AAT$_{DSF}$*-02-5*	*Glyma02g15561*	4	9.3	0.01	2.3			*Fflr 16-2*	⑩
g-AAT$_{DSF}$*-02-6*	*Glyma02g37350*	3	21.8	0.65	169.4			*Fflr 24-1*	⑦
g-AAT$_{DSF}$*-02-7*	*Glyma02g45790*	3	4.1	0.01	2				⑧
g-AAT$_{DSF}$*-02-8*	*Glyma02g46800*	2	23.6	0.78	200				⑥
g-AAT$_{DSF}$*-02-9*	*Glyma02g48010*	2	36.4	2.1	307.7				⑩
g-AAT$_{DSF}$*-03-1*	*Glyma03g06440*	5	17	0.24	65.4				⑤
g-AAT$_{DSF}$*-03-2*	*Glyma03g29230*	4	5.9	0.01	1.5				⑧
g-AAT$_{DSF}$*-03-3*	*Glyma03g33000*	4	13.7	0.12	34.1			*Dt1*(*TFL1A*,1840.5Kb)	⑩
g-AAT$_{DSF}$*-03-4*	*Glyma03g41520*	2	5.7	0.02	7.4				⑩
g-AAT$_{DSF}$*-03-5*	*Glyma03g42060*	3	7.9	0.02	4.6			*LHY2b*(130.6Kb)	⑩
g-AAT$_{DSF}$*-04-1*	*Glyma04g02740*	3	19.7	1.0E-03	105.2				⑦
g-AAT$_{DSF}$*-04-2*	*Glyma04g02850*	4	16.6	0.22	62.5				⑤/⑧
g-AAT$_{DSF}$*-04-3*	*Glyma04g05600*	2	12.3	0.1	32			*J(GmELF3*,232.7Kb)	①/⑧
g-AAT$_{DSF}$*-04-4*	*Glyma04g35730*	4	5.9	0.01	3.8				⑥
g-AAAT$_{DSF}$*-04-5*	*Glyma04g36240*	3	31.4	1.16	275.3				⑩
g-AAT$_{DSF}$*-04-6*	*Glyma04g36630*	2	22.2	3.2E-03	167.1				④/①/⑦
g-AAT$_{DSF}$*-04-7*	*Glyma04g42981*	2	9.3	0.05	16.9				⑩
g-AAT$_{DSF}$*-05-1*	*Glyma05g02470*	2	21.7	1.5E-03	158.2				⑥/⑧
g-AAT$_{DSF}$*-05-2*	*Glyma05g31250*	3	14	0.09	25.7				③
g-AAT$_{DSF}$*-06-1*	*Glyma06g04140*	2	22.6	0.66	174.4				⑩
g-AAT$_{DSF}$*-06-2*	*Glyma06g11370*	4	10.2	0.05	15.2				⑩
g-AAT$_{DSF}$*-06-3*	*Glyma06g16930*	3	15.5	0.23	66.1				⑧
g-AAT$_{DSF}$*-06-4*	*Glyma06g19651*	2	3.2	0.01	4.5			*Fflr 26-3,26-4*	⑧
g-AAT$_{DSF}$*-06-5*	*Glyma06g20950*	2	7.1	0.01	4.9			*Fflr 26-2,26-5*	⑤
g-AAT$_{DSF}$*-06-6*	*Glyma06g23580*	4	56.3	4.41	307.7			*E1*(696.3Kb),*Fflr 8-1,9-1,10-1,20-1, 22-2,23-1,26-10,26-11,26-13,26-15,26-16,26-17*	⑩
g-AAT$_{DSF}$*-06-7*	*Glyma06g32870*	4	18.1	0.27	74.3			*Fflr 3-1,7-1,8-1,9-1,10-1,18-1,20-1, 22-2,23-1,26-10,26-13,26-16,26-18*	⑩
g-AAT$_{DSF}$*-06-8*	*Glyma06g36380*	2	35.6	1.91	307.7			*Fflr 3-1,7-1,8-1,9-1,10-1,18-1,20-1, 22-2,23-1,26-10,26-13,26-16,26-18*	④
g-AAT$_{DSF}$*-06-9*	*Glyma06g40670*	4	17.4	4.4E-04	72			*Fflr 2-1,9-1,10-1,20-1*	⑧
g-AAT$_{DSF}$*-06-10*	*Glyma06g42730*	2	5.5	0.01	4.5			*Fflr 1-1,2-1,9-1,10-1,20-1*	⑥
g-AAT$_{DSF}$*-06-11*	*Glyma06g45220*	2	4.3	0.02	6.4				⑩
g-AAT$_{DSF}$*-06-12*	*Glyma06g47590*	4	12.3	0.04	10.1				⑩
g-AAT$_{DSF}$*-07-1*	*Glyma07g00520*	4	8.4	0.05	14.3				⑤/③/⑦/⑧
g-AAT$_{DSF}$*-07-2*	*Glyma07g09170*	8	19	3.6E-03	140.1			*Fflr 2-2*	⑩

g-AAT$_{DSF}$*-07-3*	*Glyma07g10060*	5	12.5	0.11	29.4			*Fflr 2-2*	⑩
g-AAT$_{DSF}$*-07-4*	*Glyma07g14870*	3	11.7	6.1E-04	12.4				⑤
g-AAT$_{DSF}$*-07-5*	*Glyma07g30140*	2	2.5	0.01	2.5				⑤
g-AAT$_{DSF}$*-07-6*	*Glyma07g36065*	3	16.6	0.27	75.9				⑥/⑧
g-AAT$_{DSF}$*-08-1*	*Glyma08g03210*	4	64.5	6.14	307.7				⑨
g-AAT$_{DSF}$*-08-2*	*Glyma08g04320*	3	18.4	0.32	91				⑩
g-AAT$_{DSF}$*-08-3*	*Glyma08g13330*	3	9.7	0.03	9.4				⑤/⑧
g-AAT$_{DSF}$*-08-4*	*Glyma08g14150*	3	3.2	0.01	2.8				⑩
g-AAT$_{DSF}$*-08-5*	*Glyma08g20140*	4	19.4	0.31	85.1				④/⑤
g-AAT$_{DSF}$*-08-6*	*Glyma08g28890*	2	1.8	3.3E-03	1.5			*COL2a*(578.7Kb)	①/⑦
g-AAT$_{DSF}$*-08-7*	*Glyma08g43140*	2	21.4	0.63	166.7				⑩
g-AAT$_{DSF}$*-08-8*	*Glyma08g43930*	3	15.9	0.26	72.7				⑩
g-AAT$_{DSF}$*-09-1*	*Glyma09g02470*	3	38.6	2.95	307.7	0.27	71.3		⑧
g-AAT$_{DSF}$*-09-2*	*Glyma09g07661*	2	20	3.2E-03	118.9			*Fflr 24-2*	③
g-AAT$_{DSF}$*-09-3*	*Glyma09g07760*	5	15	0.14	38.2			*Fflr 24-2*	⑩
g-AAT$_{DSF}$*-09-4*	*Glyma09g25215*	2	19.4	1.6E-03	94			*Fflr 24-2*	④/⑤/⑥
g-AAT$_{DSF}$*-09-5*	*Glyma09g27940*	4	9.2	0.05	14.7			*Fflr 24-2*	⑥
g-AAT$_{DSF}$*-09-6*	*Glyma09g28620*	4	21.5	3.5E-03	160.6				⑥
g-AAT$_{DSF}$*-09-7*	*Glyma09g32350*	3	18	3.1E-03	113.8				⑩
g-AAT$_{DSF}$*-09-8*	*Glyma09g34850*	4	31.1	1.85	307.7				⑨
g-AAT$_{DSF}$*-10-1*	*Glyma10g24391*	4	20.9	3.3E-03	115.8			*Fflr 13-10*	⑤
g-AAT$_{DSF}$*-10-2*	*Glyma10g26450*	5	30.9	1.79	307.7			*E4(PhyA,2112.8Kb),Fflr 13-10*	④/⑤/②/③
g-AAT$_{DSF}$*-10-3*	*Glyma10g34490*	2	1.9	0.01	2.8				⑨
g-AAT$_{DSF}$*-10-4*	*Glyma10g35960*	2	28.3	0.99	245.1			*E2*(*GmGI*,545.6Kb),*Fflr 24-4*	⑦
g-AAT$_{DSF}$*-11-1*	*Glyma11g05990*	3	11.6	1.6E-03	19.6				⑩
g-AAT$_{DSF}$*-11-2*	*Glyma11g10490*	3	9.3	0.03	10.4				⑩
g-AAT$_{DSF}$*-11-3*	*Glyma11g14500*	3	39	1.79	307.7				⑩
g-AAT$_{DSF}$*-11-4*	*Glyma11g19670*	3	31.8	1.11	267.2			*Fflr 8-14*	⑧
g-AAT$_{DSF}$*-11-5*	*Glyma11g25900*	2	14	0.12	36				③
g-AAT$_{DSF}$*-12-1*	*Glyma12g08000*	4	29.9	1.73	307.7			*PRR3b(152.2Kb),Fflr 25-2*	⑩
g-AAT$_{DSF}$*-12-2*	*Glyma12g10880*	3	18.3	6.9E-04	48.3				⑩
g-AAT$_{DSF}$*-12-3*	*Glyma12g34830*	3	11.4	0.08	24.2				⑩
g-AAT$_{DSF}$*-13-1*	*Glyma13g02620*	3	8.4	0.01	2.8				④/⑤
g-AAT$_{DSF}$*-13-2*	*Glyma13g07110*	3	99.3	6.25	307.7				⑦
g-AAT$_{DSF}$*-13-3*	*Glyma13g21340*	3	12.3	0.07	19.6				⑧
g-AAT$_{DSF}$*-13-4*	*Glyma13g23850*	2	18.1	5.0E-03	53.4				⑤/③/⑧
g-AAT$_{DSF}$*-13-5*	*Glyma13g25480*	6	18.7	1.3E-03	133.3				④
g-AAT$_{DSF}$*-13-6*	*Glyma13g29020*	2	18.1	6.1E-04	121.3				⑩
g-AAT$_{DSF}$*-13-7*	*Glyma13g41650*	3	9.6	0.01	4.3				⑥
g-AAT$_{DSF}$*-13-8*	*Glyma13g43320*	4	14.8	0.15	43.9				④/①
g-AAT$_{DSF}$*-14-1*	*Glyma14g00240*	4	13.6	0.15	41.7				②
g-AAT$_{DSF}$*-14-2*	*Glyma14g04690*	2	30.8	0.96	238.2				⑥
g-AAT$_{DSF}$*-14-3*	*Glyma14g05140*	2	4.1	0.02	5.9				⑩
g-AAT$_{DSF}$*-14-4*	*Glyma14g09720*	2	6.7	0.03	10.4				⑩
g-AAT$_{DSF}$*-14-5*	*Glyma14g36980*	2	20.1	2.7E-03	115.9				⑥
g-AAT$_{DSF}$*-14-6*	*Glyma14g39375*	3	18.9	3.9E-03	134.4				⑩
g-AAT$_{DSF}$*-15-1*	*Glyma15g00460*	2	11.9	3.9E-03	22.5				⑧
g-AAT$_{DSF}$*-15-2*	*Glyma15g04891*	2	11.5	0.09	26.7				⑩
g-AAT$_{DSF}$*-15-3*	*Glyma15g08420*	2	8.9	0.04	14.1				③/⑧
g-AAT$_{DSF}$*-15-4*	*Glyma15g12930*	2	14.1	0.15	44.1			*Fflr 12-3*	⑤/①
g-AAT$_{DSF}$*-15-5*	*Glyma15g13410*	2	4.4	0.02	7.3			*Fflr 12-3*	⑩

g-AAT_{DSF}*-15-6*	*Glyma15g18840*	2	4.8	0.01	4.5				⑩
g-AAT_{DSF}*-15-7*	*Glyma15g34840*	3	42.2	3.22	307.7				②/⑧
g-AAT_{DSF}*-15-8*	*Glyma15g35941*	5	17.4	0.32	88.7				⑩
g-AAT_{DSF}*-15-9*	*Glyma15g42762*	4	8.7	0.07	21.3				⑩
g-AAT_{DSF}*-15-10*	*Glyma15g43010*	2	14.4	0.1	31				⑩
g-AAT_{DSF}*-16-1*	*Glyma16g00480*	2	11.9	0.08	23.8			*LHY1a*(1336.7Kb)	⑤
g-AAT_{DSF}*-16-2*	*Glyma16g03320*	2	37.6	2.3	307.7	0.17	48.4		①/③/⑧
g-AAT_{DSF}*-16-3*	*Glyma16g03860*	2	15.4	0.18	52.1			*GmFT5a(1280.3Kb), LHY1a(1306.3Kb),GmFT3a(1307.0*	⑩
g-AAT_{DSF}*-16-4*	*Glyma16g08430*	4	10.1	0.05	13.7			*Fflr 13-7*	⑤
g-AAT_{DSF}*-16-5*	*Glyma16g28950*	2	16.5	0.25	73.6				⑩
g-AAT_{DSF}*-16-6*	*Glyma16g32650*	3	17.6	0.25	70.6			*Fflr 13-8*	①
g-AAT_{DSF}*-16-7*	*Glyma16g34500*	2	1.4	0.01	2.5			*Fflr 13-8*	⑥
g-AAT_{DSF}*-17-1*	*Glyma17g05595*	3	22.4	0.79	201.7				⑩
g-AAT_{DSF}*-17-2*	*Glyma17g07475*	2	2.9	0.01	3.1				⑥
g-AAT_{DSF}*-17-3*	*Glyma17g08460*	2	76.8	7.32	307.7				⑩
g-AAT_{DSF}*-17-4*	*Glyma17g09500*	3	38.2	2.3	307.7				⑩
g-AAT_{DSF}*-18-1*	*Glyma18g01200*	2	11.9	0.04	13.9				⑩
g-AAT_{DSF}*-18-2*	*Glyma18g03180*	3	13.5	0.07	21				⑩
g-AAT_{DSF}*-18-3*	*Glyma18g08410*	5	31.3	1.61	307.7			*Fflr 9-2*	⑥
g-AAT_{DSF}*-18-4*	*Glyma18g08440*	3	16.4	0.11	31.3			*Fflr 9-2*	⑧
g-AAT_{DSF}*-18-5*	*Glyma18g17395*	4	31.3	1.59	307.7				⑧
g-AAT_{DSF}*-18-6*	*Glyma18g17515*	3	4.7	0.02	4.9				⑩
g-AAT_{DSF}*-18-7*	*Glyma18g18931*	2	23.9	1.6E-03	169.1				⑨
g-AAT_{DSF}*-18-8*	*Glyma18g20820*	3	9.5	0.05	13.8			*Fflr10-2*	⑥
g-AAT_{DSF}*-18-9*	*Glyma18g26120*	2	2.5	3.8E-03	1.7			*Fflr10-2*	⑥
g-AAT_{DSF}*-18-10*	*Glyma18g48980*	3	8.9	0.05	16.2				⑧
g-AAT_{DSF}*-18-11*	*Glyma18g50910*	5	8.3	0.03	7.6				⑤/①
g-AAT_{DSF}*-18-12*	*Glyma18g52381*	4	5.3	0.02	4.2			*GmFT1a*(939.7Kb),*GmFT1b*(956.5 K*b)*	⑩
g-AAT_{DSF}*-19-1*	*Glyma19g23640*	4	9.9	0.02	4				⑤/②
g-AAT_{DSF}*-19-2*	*Glyma19g36710*	2	6	0.02	6.4			*Fflr 2-3,22-3,24-3*	⑤/②
g-AAT_{DSF}*-19-3*	*Glyma19g37840*	2	9.1	0.02	8.2			*Fflr 2-3,3-3,22-3,24-3*	⑩
g-AAT_{DSF}*-19-4*	*Glyma19g38331*	2	13.8	0.11	34.1			*Fflr 3-3,22-3*	⑧
g-AAT_{DSF}*-19-5*	*Glyma19g40710*	2	16.2	0.24	69.7			*E3*(*GmphyA3*,463.8Kb),*Fflr 13-9*	⑩
g-AAT_{DSF}*-19-6*	*Glyma19g42340*	2	4	3.2E-03	1.5			*LHY2a(1912.4Kb),Fflr 5-3,8-3,20-2*	①/⑧
g-AAT_{DSF}*-20-1*	*Glyma20g00565*	2	11.2	0.05	15.6				⑩
g-AAT_{DSF}*-20-2*	*Glyma20g20053*	3	20.7	0.31	86.9			*Fflr 16-3,20-3,25-3*	⑤/①
g-AAT_{DSF}*-20-3*	*Glyma20g24830*	6	18.5	0.34	92.5			*Fflr 25-3*	⑦
g-AAT_{DSF}*-20-4*	*Glyma20g24860*	3	18.2	8.3E-04	119.3			*Fflr 25-3*	⑩
g-AAT_{DSF}*-20-5*	*Glyma20g26360*	2	3.7	0.01	2.7			*Fflr 25-3*	⑩
Total	130	384(2.95)		73.76		0.55		65(47)	**162**

Note: Gene code: for example, *g-*AAT_{DSF}*-01-1*, where AAT_{DSF} means DSF required accumulative active temperature, -01 represents chromosome 1, and -1 represents its order on the chromosome according to its physical position. The position corresponds to the Williams 82 reference genome version 1 (Wm82.a1). Gene group: Gene Ontology groups (please see the notes of Table S2 for details).

Table S6 The gene–allele system conferring ADL$_{DFM}$ identified from GASM-RTM-GWAS in the WSGP.

Gene Code	Genes	Allele no.	Model P -lg(P)	Main-effect		Gene×Env.		Known Genes/QTLs	Gene group
				R^2	-lg(P)	R^2	type		
g-ADL$_{DFM}$*-01-1*	*Glyma01g02525*	4	2.2	0.01	1.3				⑤/⑦/⑨
g-ADL$_{DFM}$*-01-2*	*Glyma01g33986*	2	7.6	0.04	5.5				②/⑧
g-ADL$_{DFM}$*-01-3*	*Glyma01g36545*	3	11.6	0.20	25.8				⑩
g-ADL$_{DFM}$*-02-1*	*Glyma02g00371*	4	86.7	2.72	281.1	8.32	307.7		⑧
g-ADL$_{DFM}$*-02-2*	*Glyma02g08091*	2	14.0	0.21	28.2				⑩
g-ADL$_{DFM}$*-02-3*	*Glyma02g08620*	3	13.2	0.30	38.3				⑧
g-ADL$_{DFM}$*-02-4*	*Glyma02g31295*	3	2.6	0.02	2.7			*Rsl 7-1*	⑤
g-ADL$_{DFM}$*-02-5*	*Glyma02g36665*	2	13.6	0.01	2.6				⑩
g-ADL$_{DFM}$*-02-6*	*Glyma02g43025*	2	4.9	0.06	8.3				⑧/⑨
g-ADL$_{DFM}$*-02-7*	*Glyma02g46810*	2	12.5	0.20	26.6				⑥
g-ADL$_{DFM}$*-03-1*	*Glyma03g02500*	3	15.7	0.44	55.8				⑤
g-ADL$_{DFM}$*-03-2*	*Glyma03g02940*	6	9.6	0.10	10.9				⑩
g-ADL$_{DFM}$*-03-3*	*Glyma03g04590*	5	2.8	0.02	2.2				⑦
g-ADL$_{DFM}$*-03-4*	*Glyma03g06483*	3	32.6	1.31	152.8	1.51	167.9	*Dt1*(*TFL1a*,2033.9Kb)	⑤/⑦/⑥
g-ADL$_{DFM}$*-03-5*	*Glyma03g27290*	2	3.9	0.03	4.6				⑦
g-ADL$_{DFM}$*-03-6*	*Glyma03g29230*	4	15.7	0.36	45.1				⑧
g-ADL$_{DFM}$*-03-7*	*Glyma03g38171*	2	2.2	0.01	2.4				⑩
g-ADL$_{DFM}$*-03-8*	*Glyma03g39850*	3	14.4	0.40	50.3				⑥
g-ADL$_{DFM}$*-03-9*	*Glyma03g41280*	3	12.5	0.21	27.2				⑩
g-ADL$_{DFM}$*-03-10*	*Glyma03g41360*	2	6.9	0.05	8.0				⑦/⑧
g-ADL$_{DFM}$*-04-1*	*Glyma04g01990*	2	6.4	0.01	2.6				⑤
g-ADL$_{DFM}$*-04-2*	*Glyma04g05471*	5	14.6	0.17	19.9				⑧
g-ADL$_{DFM}$*-04-3*	*Glyma04g09950*	2	14.3	0.22	29.6				⑨
g-ADL$_{DFM}$*-04-4*	*Glyma04g10050*	4	21.8	0.81	97.3			*CRY1a*(953.6Kb)	⑦/⑥
g-ADL$_{DFM}$*-04-5*	*Glyma04g12320*	2	22.8	1.07	128.9				⑤
g-ADL$_{DFM}$*-04-6*	*Glyma04g33110*	3	6.2	0.05	6.2			*Rsl 2-2*,*Rsl 3-4*,*Rsl 5-1*	⑤/②
g-ADL$_{DFM}$*-04-7*	*Glyma04g36260*	3	4.3	0.01	1.4				⑧
g-ADL$_{DFM}$*-04-8*	*Glyma04g42900*	3	13.2	0.12	15.6				⑥
g-ADL$_{DFM}$*-05-1*	*Glyma05g04561*	2	30.0	1.22	145.4				⑩
g-ADL$_{DFM}$*-05-2*	*Glyma05g22680*	2	29.9	0.96	117.4				⑩
g-ADL$_{DFM}$*-05-3*	*Glyma05g24580*	2	18.1	0.47	60.8			*J*(*ELF3A*, 103.8Kb)	⑩
g-ADL$_{DFM}$*-05-4*	*Glyma05g29880*	2	18.4	0.22	29.8			*FLC*(1324.5Kb)	⑩
g-ADL$_{DFM}$*-05-5*	*Glyma05g35310*	2	8.0	0.03	5.1				⑥
g-ADL$_{DFM}$*-06-1*	*Glyma06g03470*	2	8.9	0.14	19.6				⑤
g-ADL$_{DFM}$*-06-2*	*Glyma06g05890*	2	22.1	0.60	76.3				⑥
g-ADL$_{DFM}$*-06-3*	*Glyma06g13985*	3	6.5	0.03	4.4			*WRKY76*(478.9Kb)	⑩
g-ADL$_{DFM}$*-06-4*	*Glyma06g15890*	2	3.4	0.03	4.1				②/⑧
g-ADL$_{DFM}$*-06-5*	*Glyma06g19480*	2	19.1	0.52	66.4				②/⑦/⑨
g-ADL$_{DFM}$*-06-6*	*Glyma06g23580*	4	9.5	0.08	10.4			*E1*(696.3Kb)	⑩
g-ADL$_{DFM}$*-06-7*	*Glyma06g40670*	4	18.9	0.60	73.7				⑧
g-ADL$_{DFM}$*-07-1*	*Glyma07g05290*	2	4.2	0.01	2.0				⑩
g-ADL$_{DFM}$*-07-2*	*Glyma07g09420*	5	26.1	1.56	175.2				⑧
g-ADL$_{DFM}$*-07-3*	*Glyma07g10541*	3	11.8	0.18	24.1				⑧
g-ADL$_{DFM}$*-07-4*	*Glyma07g10621*	6	17.1	0.44	52.2				⑧
g-ADL$_{DFM}$*-07-5*	*Glyma07g11091*	3	13.0	0.10	12.6				⑩
g-ADL$_{DFM}$*-07-6*	*Glyma07g29650*	2	20.3	0.62	77.9				⑨

g-ADL$_{DFM}$*-07-7*	*Glyma07g39110*	3	13.0	0.18	23.4				⑩
g-ADL$_{DFM}$*-07-8*	*Glyma07g40260*	2	26.4	1.00	121.2				⑩
g-ADL$_{DFM}$*-08-1*	*Glyma08g02210*	3	20.9	0.68	84.4				⑦/⑥
g-ADL$_{DFM}$*-08-2*	*Glyma08g15400*	2	8.1	0.03	4.8				⑩
g-ADL$_{DFM}$*-08-3*	*Glyma08g15870*	2	31.1	1.45	168.7				⑩
g-ADL$_{DFM}$*-08-4*	*Glyma08g23740*	2	3.1	0.01	1.9				⑥
g-ADL$_{DFM}$*-08-5*	*Glyma08g46480*	2	15.1	0.22	29.1			*E10*(1040.3Kb),*FT6*(1900.6Kb)	⑩
g-ADL$_{DFM}$*-09-1*	*Glyma09g05600*	3	7.5	0.08	11.0				⑧
g-ADL$_{DFM}$*-09-2*	*Glyma09g05830*	3	4.4	0.02	2.8				⑥
g-ADL$_{DFM}$*-09-3*	*Glyma09g07760*	5	9.0	0.11	12.4				⑩
g-ADL$_{DFM}$*-09-4*	*Glyma09g26100*	2	3.3	0.01	2.4			*TFL1.4*(675.2Kb)	⑦/⑥
g-ADL$_{DFM}$*-09-5*	*Glyma09g31850*	2	3.9	0.01	1.8				②
g-ADL$_{DFM}$*-09-6*	*Glyma09g33010*	2	18.9	0.44	57.6				②
g-ADL$_{DFM}$*-09-7*	*Glyma09g33060*	3	5.6	0.10	12.5				①
g-ADL$_{DFM}$*-09-8*	*Glyma09g41010*	2	8.0	0.09	12.5				⑦/⑥
g-ADL$_{DFM}$*-10-1*	*Glyma10g05100*	2	7.5	0.04	6.2				⑩
g-ADL$_{DFM}$*-10-2*	*Glyma10g05451*	2	9.6	0.12	16.3				⑩
g-ADL$_{DFM}$*-10-3*	*Glyma10g07601*	2	2.8	0.02	2.7				④/⑤
g-ADL$_{DFM}$*-10-4*	*Glyma10g11480*	2	8.9	0.12	16.4				⑨
g-ADL$_{DFM}$*-10-5*	*Glyma10g17510*	3	10.6	0.13	16.9				⑩
g-ADL$_{DFM}$*-10-6*	*Glyma10g29970*	4	20.8	0.97	115.0			*PhyA1*(1707.7Kb)	①/⑥
g-ADL$_{DFM}$*-10-7*	*Glyma10g36600*	2	20.2	0.55	69.7			*RAV*(1780.3Kb),*Rsl 8-2*,*Rsl 9-2*	⑤/②/①
g-ADL$_{DFM}$*-10-8*	*Glyma10g42161*	2	14.8	0.34	44.7				④
g-ADL$_{DFM}$*-11-1*	*Glyma11g10600*	4	7.7	0.10	12.9				⑧
g-ADL$_{DFM}$*-11-2*	*Glyma11g10800*	4	13.5	0.35	43.4				⑩
g-ADL$_{DFM}$*-12-1*	*Glyma12g02340*	3	14.3	0.14	18.5				⑤
g-ADL$_{DFM}$*-12-2*	*Glyma12g06580*	3	11.6	0.18	23.6				⑧
g-ADL$_{DFM}$*-12-3*	*Glyma12g06950*	2	2.0	0.01	1.7				⑩
g-ADL$_{DFM}$*-12-4*	*Glyma12g08390*	2	2.0	0.01	2.2				⑨
g-ADL$_{DFM}$*-12-5*	*Glyma12g35580*	2	17.8	0.62	78.1				④
g-ADL$_{DFM}$*-13-1*	*Glyma13g09470*	5	61.0	1.14	132.4	5.75	307.7		⑩
g-ADL$_{DFM}$*-13-2*	*Glyma13g25020*	5	7.0	0.05	5.4				⑩
g-ADL$_{DFM}$*-13-3*	*Glyma13g25440*	3	5.5	0.03	4.5			*Rsl 9-2*	⑦
g-ADL$_{DFM}$*-13-4*	*Glyma13g25480*	6	48.8	2.16	230.5	3.32	316.4		④
g-ADL$_{DFM}$*-13-5*	*Glyma13g28880*	4	13.2	0.34	42.0				⑤/⑥
g-ADL$_{DFM}$*-13-6*	*Glyma13g34460*	3	7.8	0.04	5.6				⑤
g-ADL$_{DFM}$*-13-7*	*Glyma13g36310*	2	1.8	0.02	3.0				④
g-ADL$_{DFM}$*-14-1*	*Glyma14g13884*	3	9.7	0.13	16.8				⑧
g-ADL$_{DFM}$*-14-2*	*Glyma14g39230*	2	7.7	0.09	12.8				⑨
g-ADL$_{DFM}$*-15-1*	*Glyma15g08420*	2	11.6	0.11	15.5				⑧
g-ADL$_{DFM}$*-15-2*	*Glyma15g34653*	4	20.4	0.36	44.7				⑥
g-ADL$_{DFM}$*-16-1*	*Glyma16g03050*	3	1.6	0.01	1.9			*FT5A*(1469.9KB),*FT3A*(1496.7Kb)	②/⑦
g-ADL$_{DFM}$*-16-2*	*Glyma16g07920*	5	9.5	0.05	5.8				④⑧
g-ADL$_{DFM}$*-16-3*	*Glyma16g21340*	2	3.3	0.03	4.9				①
g-ADL$_{DFM}$*-16-4*	*Glyma16g26070*	3	13.7	0.23	29.2			*FT2A*(500.9Kb),*FT2B*(539.8Kb)	⑧
g-ADL$_{DFM}$*-16-5*	*Glyma16g27521*	5	24.0	1.38	157.6				⑥
g-ADL$_{DFM}$*-16-6*	*Glyma16g29380*	5	12.6	0.20	24.3				⑩
g-ADL$_{DFM}$*-16-7*	*Glyma16g31862*	6	6.1	0.05	4.9			*BFT*(137.7Kb)	⑤
g-ADL$_{DFM}$*-16-8*	*Glyma16g33100*	4	30.3	1.39	159.8				⑧

g-ADL$_{DFM}$*-17-1*	*Glyma17g01160*	3	13.9	0.20	26.6				⑥
g-ADL$_{DFM}$*-17-2*	*Glyma17g02610*	2	13.8	0.30	40.3				⑩
g-ADL$_{DFM}$*-17-3*	*Glyma17g04580*	3	15.9	0.36	46.2				⑩
g-ADL$_{DFM}$*-17-4*	*Glyma17g07520*	2	7.0	0.01	1.5				⑩
g-ADL$_{DFM}$*-17-5*	*Glyma17g19084*	4	12.5	0.10	12.2			*Rsl 5-2*	②
g-ADL$_{DFM}$*-17-6*	*Glyma17g20091*	2	3.6	0.02	2.7				⑩
g-ADL$_{DFM}$*-17-7*	*Glyma17g34420*	2	5.9	0.02	3.9				⑩
g-ADL$_{DFM}$*-18-1*	*Glyma18g18230*	3	11.6	0.10	13.0				⑩
g-ADL$_{DFM}$*-18-2*	*Glyma18g42375*	5	18.7	0.43	52.7			*Rp 1-3*	⑤
g-ADL$_{DFM}$*-18-3*	*Glyma18g44761*	4	19.9	0.86	103.0			*Rsl 5-2*	②/①/⑧
g-ADL$_{DFM}$*-18-4*	*Glyma18g45930*	2	9.5	0.11	15.9				⑦
g-ADL$_{DFM}$*-18-5*	*Glyma18g51380*	2	20.9	0.63	79.4				⑥
g-ADL$_{DFM}$*-18-6*	*Glyma18g53545*	3	13.1	0.24	30.5			*FT1A*(85.1Kb),*FT1B*(101.9Kb)	⑤/①
g-ADL$_{DFM}$*-19-1*	*Glyma19g03590*	4	6.3	0.10	12.1				⑤/⑥
g-ADL$_{DFM}$*-19-2*	*Glyma19g23740*	2	21.2	0.93	113.8				⑩
g-ADL$_{DFM}$*-19-3*	*Glyma19g25360*	2	27.0	0.91	111.2				⑤/ ①/⑨
g-ADL$_{DFM}$*-19-4*	*Glyma19g30690*	3	71.8	7.18	307.7			*FT5B*(2470.9Kb),*FT3B*(2489.9Kb)	⑩
g-ADL$_{DFM}$*-19-5*	*Glyma19g33210*	3	6.3	0.02	2.9				⑥
g-ADL$_{DFM}$*-19-6*	*Glyma19g34740*	2	44.6	2.67	280.3			,2033.9Kb),*Rsl 5-6*	⑤/⑦
g-ADL$_{DFM}$*-19-7*	*Glyma19g40710*	2	11.5	0.22	29.3				⑩
g-ADL$_{DFM}$*-20-1*	*Glyma20g02500*	3	3.7	0.05	6.7				⑩
g-ADL$_{DFM}$*-20-2*	*Glyma20g03100*	5	22.5	0.65	78.9				⑩
g-ADL$_{DFM}$*-20-3*	*Glyma20g23440*	2	1.4	0.01	1.9				⑩
g-ADL$_{DFM}$*-20-4*	*Glyma20g25100*	3	11.9	0.15	19.4				⑥
g-ADL$_{DFM}$*-20-5*	*Glyma20g26460*	3	8.3	0.02	2.8				⑩
g-ADL$_{DFM}$*-20-6*	*Glyma20g27950*	2	27.8	0.53	67.4	1.46	166.8		⑧
g-ADL$_{DFM}$*-20-7*	*Glyma20g32390*	3	15.3	0.42	53.2			*GIGANTEA*(1339.9Kb)	⑧/⑨
g-ADL$_{DFM}$*-20-8*	*Glyma20g32540*	2	3.3	0.02	2.9				⑤
g-ADL$_{DFM}$*-20-9*	*Glyma20g32720*	3	5.5	0.07	9.3				⑩
Total	124	364(2.94)		50.24		20.36		33(23)	154

Note: Gene code: for example, *g-ADL*$_{DFM}$*-01-1,* where *ADL*$_{DFM}$ means DFM required accumulative day-length, -01 represents chromosome 1, and -1 represents its order on the chromosome according to its physical position. The position corresponds to the Williams 82 reference genome version 1 (Wm82.a1).

Reported QTL, the QTL name is simplified; for example, *Rp1-3* represents reproductive period *1-3* and *Rsl7-1* represents reproductive stage length *7-1*. The same applies below. Gene group: Gene Ontology groups (please see the notes of Table S2 for details).

Table S7 The gene–allele system conferring AAT$_{DFM}$ identified from GASM-RTM-GWAS in the WSGP.

Gene Code	Genes	Allele no.	Model P -lg(P)	Main-effect		Gene×Env.		Known Genes/QTLs	Gene group
				R2	-lg(P)	R2	-lg(P)		
g-AAT$_{DFM}$*-01-1*	*Glyma01g04515*	2	2.2	0.02	3.9				⑩
g-AAT$_{DFM}$*-01-2*	*Glyma01g28850*	2	12.1	0.05	8.9				⑩
g-AAT$_{DFM}$*-01-3*	*Glyma01g43900*	3	9.1	0.09	13.6				⑥/⑧
g-AAT$_{DFM}$*-02-1*	*Glyma02g00371*	4	50.9	1.33	174.7	3.80	307.7	*Rsl 7-1*	⑧
g-AAT$_{DFM}$*-02-2*	*Glyma02g01540*	2	18.1	0.67	95.7				④/⑤/⑥
g-AAT$_{DFM}$*-02-3*	*Glyma02g05550*	5	6.6	0.06	8.4				⑧
g-AAT$_{DFM}$*-02-4*	*Glyma02g06730*	4	14.6	0.22	31.8				⑤/①
g-AAT$_{DFM}$*-02-5*	*Glyma02g06841*	2	36.8	1.56	203.2	1.08	145.5		⑩
g-AAT$_{DFM}$*-02-6*	*Glyma02g08430*	3	19.1	0.28	42.1				⑥
g-AAT$_{DFM}$*-02-7*	*Glyma02g15470*	3	7.7	0.05	8.3				⑩
g-AAT$_{DFM}$*-02-8*	*Glyma02g19000*	2	1.7	0.01	2.3			*FLD*(443.3Kb)	⑤/⑦
g-AAT$_{DFM}$*-02-9*	*Glyma02g42290*	2	16.0	0.33	50.5				⑦
g-AAT$_{DFM}$*-02-10*	*Glyma02g46400*	2	3.5	0.01	1.9				⑩
g-AAT$_{DFM}$*-03-1*	*Glyma03g05120*	2	39.9	0.80	112.7	3.15	307.7		⑩
g-AAT$_{DFM}$*-03-2*	*Glyma03g18725*	5	3.6	0.05	6.9				⑩
g-AAT$_{DFM}$*-03-3*	*Glyma03g20420*	2	1.6	0.01	2.3				⑥
g-AAT$_{DFM}$*-03-4*	*Glyma03g29010*	4	23.2	1.20	158.9	0.41	53.5		⑥
g-AAT$_{DFM}$*-03-5*	*Glyma03g29060*	3	5.5	0.03	4.6				⑩
g-AAT$_{DFM}$*-03-6*	*Glyma03g34900*	2	19.0	0.70	100.6			*Dt1*(*TFL1A*, 326.8Kb)	⑤/③
g-AAT$_{DFM}$*-04-1*	*Glyma04g02980*	3	4.3	0.02	3.6				⑤/①
g-AAT$_{DFM}$*-04-2*	*Glyma04g06240*	4	12.2	0.20	29.5			*J*(*ELF3A*, 742.0Kb)	⑤/⑦
g-AAT$_{DFM}$*-04-3*	*Glyma04g16100*	6	7.9	0.02	2.2				①
g-AAT$_{DFM}$*-04-4*	*Glyma04g20400*	4	9.4	0.04	6.2				⑩
g-AAT$_{DFM}$*-04-5*	*Glyma04g36790*	2	1.4	0.01	2.8				⑦
g-AAT$_{DFM}$*-04-6*	*Glyma04g38955*	3	11.4	0.13	18.9				⑤/⑥
g-AAT$_{DFM}$*-04-7*	*Glyma04g40310*	2	17.5	0.50	73.9				⑤/①
g-AAT$_{DFM}$*-04-8*	*Glyma04g42010*	3	13.2	0.19	29.1				⑨
g-AAT$_{DFM}$*-05-1*	*Glyma05g01943*	3	7.9	0.08	11.4				⑩
g-AAT$_{DFM}$*-05-2*	*Glyma05g04561*	2	18.5	0.58	84.5				⑩
g-AAT$_{DFM}$*-05-3*	*Glyma05g25485*	2	4.5	0.01	1.8				⑩
g-AAT$_{DFM}$*-05-4*	*Glyma05g26620*	2	5.2	0.01	2.5			*FLC*(1454.9Kb)	④/⑧
g-AAT$_{DFM}$*-06-1*	*Glyma06g11120*	2	5.7	0.04	7.3				⑦/⑧
g-AAT$_{DFM}$*-06-2*	*Glyma06g15390*	2	8.4	0.16	25.3			*WRKY76*(598.7Kb)	⑩
g-AAT$_{DFM}$*-06-3*	*Glyma06g17390*	3	27.7	1.34	176.3	1.43	181.9		⑩
g-AAT$_{DFM}$*-06-4*	*Glyma06g17451*	2	1.6	0.01	2.4				⑩
g-AAT$_{DFM}$*-06-5*	*Glyma06g19380*	2	17.5	0.18	27.8				⑩
g-AAT$_{DFM}$*-06-6*	*Glyma06g19480*	2	17.8	0.36	54.5				②/⑦/⑨
g-AAT$_{DFM}$*-06-7*	*Glyma06g40560*	3	2.9	0.02	2.6				⑧
g-AAT$_{DFM}$*-06-8*	*Glyma06g48393*	3	35.2	0.70	98.5	2.52	292.8		⑩
g-AAT$_{DFM}$*-07-1*	*Glyma07g04800*	2	16.6	0.34	51.7				⑤/⑧
g-AAT$_{DFM}$*-07-2*	*Glyma07g06420*	4	19.1	0.60	84.3	0.49	64.1	*Rsl 1-2*, *Rsl 5-4*	②
g-AAT$_{DFM}$*-07-3*	*Glyma07g10541*	3	5.9	0.03	5.0				⑧
g-AAT$_{DFM}$*-07-4*	*Glyma07g17620*	2	4.0	0.02	3.5				⑩
g-AAT$_{DFM}$*-07-5*	*Glyma07g29650*	2	15.3	0.17	26.0				⑨
g-AAT$_{DFM}$*-07-6*	*Glyma07g34820*	2	10.0	0.14	21.5				⑩
g-AAT$_{DFM}$*-07-7*	*Glyma07g40260*	2	22.2	0.57	82.4	0.51	72.9		⑩
g-AAT$_{DFM}$*-08-1*	*Glyma08g07050*	2	8.2	0.07	11.3				⑧

g-AAT$_{DFM}$*-08-2*	*Glyma08g11480*	5	22.5	0.94	126.8	0.95	120.3		④/⑧
g-AAT$_{DFM}$*-08-3*	*Glyma08g15050*	2	2.4	0.03	5.5				⑤/⑦
g-AAT$_{DFM}$*-08-4*	*Glyma08g15400*	2	7.5	0.10	15.5				⑩
g-AAT$_{DFM}$*-08-5*	*Glyma08g15870*	2	30.0	1.27	170.4	1.00	135.6		⑩
g-AAT$_{DFM}$*-08-6*	*Glyma08g24100*	4	10.2	0.11	16.2				⑩
g-AAT$_{DFM}$*-08-7*	*Glyma08g44170*	2	16.9	0.22	34.7			*E10*(2700.7Kb),	⑩
g-AAT$_{DFM}$*-09-1*	*Glyma09g26970*	2	3.4	0.01	1.3			*TFL1.4*(589.8Kb)	①
g-AAT$_{DFM}$*-09-2*	*Glyma09g33737*	2	2.2	0.01	2.7				⑧
g-AAT$_{DFM}$*-09-3*	*Glyma09g41740*	4	7.6	0.09	13.0				⑥
g-AAT$_{DFM}$*-10-1*	*Glyma10g05051*	5	11.9	0.19	26.2				①
g-AAT$_{DFM}$*-10-2*	*Glyma10g05600*	2	8.9	0.12	19.1				⑧
g-AAT$_{DFM}$*-10-3*	*Glyma10g28380*	3	6.6	0.06	8.9			*PhyA1*(315Kb)	⑤
g-AAT$_{DFM}$*-10-4*	*Glyma10g30930*	3	11.9	0.16	24.3				⑩
g-AAT$_{DFM}$*-10-5*	*Glyma10g31570*	3	9.8	0.06	8.5				⑤/⑨
g-AAT$_{DFM}$*-11-1*	*Glyma11g06565*	3	4.6	0.05	7.5				⑧
g-AAT$_{DFM}$*-11-2*	*Glyma11g09940*	2	4.7	0.02	4.1				④/①
g-AAT$_{DFM}$*-11-3*	*Glyma11g10430*	2	3.3	0.02	3.9				⑩
g-AAT$_{DFM}$*-11-4*	*Glyma11g13111*	2	9.0	0.12	19.0				⑩
g-AAT$_{DFM}$*-11-5*	*Glyma11g14500*	3	9.5	0.14	20.7				⑩
g-AAT$_{DFM}$*-11-6*	*Glyma11g17930*	2	22.2	0.69	98.9	0.51	72.5	*Rsl 8-1*	③/⑧
g-AAT$_{DFM}$*-11-7*	*Glyma11g20520*	3	15.9	0.24	35.8				⑤
g-AAT$_{DFM}$*-12-1*	*Glyma12g02800*	2	19.2	0.46	67.5				⑩
g-AAT$_{DFM}$*-12-2*	*Glyma12g06580*	3	15.0	0.28	41.3				⑧
g-AAT$_{DFM}$*-12-3*	*Glyma12g06620*	2	12.8	0.15	23.6				⑧
g-AAT$_{DFM}$*-12-4*	*Glyma12g06950*	2	14.9	0.15	23.1				①
g-AAT$_{DFM}$*-12-5*	*Glyma12g08100*	5	20.7	0.81	111.0	0.45	55.9		⑤/⑧
g-AAT$_{DFM}$*-12-6*	*Glyma12g08170*	4	12.0	0.14	20.5				④/⑤/①
g-AAT$_{DFM}$*-13-1*	*Glyma13g00960*	2	16.5	0.19	30.2				⑤
g-AAT$_{DFM}$*-13-2*	*Glyma13g09470*	5	114.2	1.27	166.0				⑩
g-AAT$_{DFM}$*-13-3*	*Glyma13g22100*	2	11.4	0.14	22.2			*Rsl 4-4, Rsl 7-2*	⑥
g-AAT$_{DFM}$*-13-4*	*Glyma13g22420*	2	16.7	0.26	40.2				④/⑤
g-AAT$_{DFM}$*-13-5*	*Glyma13g22855*	3	6.3	0.05	7.2				①
g-AAT$_{DFM}$*-13-6*	*Glyma13g24820*	4	19.3	0.46	65.7	0.41	53.1		⑩
g-AAT$_{DFM}$*-13-7*	*Glyma13g25480*	6	84.0	3.07	307.7	6.71	307.7		④
g-AAT$_{DFM}$*-13-8*	*Glyma13g28260*	3	10.8	0.18	27.1				⑩
g-AAT$_{DFM}$*-13-9*	*Glyma13g29160*	4	18.4	0.50	70.6	0.44	58.0		⑤
g-AAT$_{DFM}$*-13-10*	*Glyma13g43980*	3	8.3	0.08	12.0				⑩
g-AAT$_{DFM}$*-14-1*	*Glyma14g01300*	2	18.9	0.45	66.8				②
g-AAT$_{DFM}$*-14-2*	*Glyma14g02780*	3	11.1	0.08	12.2				⑤
g-AAT$_{DFM}$*-14-3*	*Glyma14g04930*	3	14.1	0.23	34.8				⑤
g-AAT$_{DFM}$*-14-4*	*Glyma14g05140*	2	7.8	0.07	11.2				⑩
g-AAT$_{DFM}$*-14-5*	*Glyma14g06485*	2	11.7	0.04	6.7				②
g-AAT$_{DFM}$*-14-6*	*Glyma14g08990*	2	13.9	0.20	31.4				⑩
g-AAT$_{DFM}$*-14-7*	*Glyma14g09160*	3	28.3	0.13	19.9	2.37	279.0		⑤
g-AAT$_{DFM}$*-14-8*	*Glyma14g13178*	3	5.0	0.03	5.2				①
g-AAT$_{DFM}$*-14-9*	*Glyma14g37230*	2	18.7	0.36	53.4				②
g-AAT$_{DFM}$*-14-10*	*Glyma14g38570*	5	8.4	0.05	5.8				⑩
g-AAT$_{DFM}$*-15-1*	*Glyma15g02740*	3	18.6	0.42	61.0				⑤
g-AAT$_{DFM}$*-15-2*	*Glyma15g09930*	2	1.4	0.01	1.6				⑩
g-AAT$_{DFM}$*-15-3*	*Glyma15g14040*	2	6.0	0.01	1.7				⑩

g-AAT$_{DFM}$*-15-4*	*Glyma15g15105*	3	24.2	0.98	134.3	1.14	148.9		⑦
g-AAT$_{DFM}$*-15-5*	*Glyma15g16183*	2	11.4	0.19	29.0				⑩
g-AAT$_{DFM}$*-15-6*	*Glyma15g18280*	3	2.3	0.01	1.9				⑩
g-AAT$_{DFM}$*-16-1*	*Glyma16g07081*	2	7.3	0.04	6.2				⑩
g-AAT$_{DFM}$*-16-2*	*Glyma16g25500*	2	23.9	0.99	136.9			*E9*(1225.6Kb),*FT2B*(1264.5Kb	⑩
g-AAT$_{DFM}$*-16-3*	*Glyma16g28900*	6	11.7	0.17	22.9				⑥
g-AAT$_{DFM}$*-16-4*	*Glyma16g32900*	2	8.0	0.06	10.2			*TFL1.3*(728.9Kb)	⑧
g-AAT$_{DFM}$*-16-5*	*Glyma16g33100*	4	18.2	0.48	68.4	0.60	79.3		⑧
g-AAT$_{DFM}$*-16-6*	*Glyma16g33490*	2	9.3	0.03	4.7				⑥
g-AAT$_{DFM}$*-17-1*	*Glyma17g01160*	3	15.1	0.05	7.7				⑥
g-AAT$_{DFM}$*-17-2*	*Glyma17g03310*	2	9.8	0.07	11.5				⑤
g-AAT$_{DFM}$*-17-3*	*Glyma17g03700*	3	7.2	0.02	2.9				①/⑦
g-AAT$_{DFM}$*-17-4*	*Glyma17g13260*	2	6.0	0.02	3.3			*GmYABBY16*(1308.5Kb)	⑤
g-AAT$_{DFM}$*-17-5*	*Glyma17g19084*	4	4.7	0.04	6.2			*Rp 1-7*	②/①
g-AAT$_{DFM}$*-17-6*	*Glyma17g33290*	2	1.3	0.01	2.0				⑩
g-AAT$_{DFM}$*-17-7*	*Glyma17g35000*	3	21.1	0.65	91.8	0.46	62.7		⑥
g-AAT$_{DFM}$*-18-1*	*Glyma18g32696*	2	1.8	0.01	2.7			*Rp 1-3*	⑤
g-AAT$_{DFM}$*-18-2*	*Glyma18g51230*	3	9.7	0.01	1.3				⑥
g-AAT$_{DFM}$*-18-3*	*Glyma18g51970*	5	18.3	0.56	77.9				⑨
g-AAT$_{DFM}$*-18-4*	*Glyma18g53770*	3	19.6	0.31	45.5			*FT1B*(84.4Kb),*FT1A*(98.2Kb)	⑩
g-AAT$_{DFM}$*-19-1*	*Glyma19g09700*	2	21.9	0.65	93.3				⑩
g-AAT$_{DFM}$*-19-2*	*Glyma19g18581*	3	6.6	0.01	1.5				⑦/⑧
g-AAT$_{DFM}$*-19-3*	*Glyma19g30750*	2	59.2	5.71	307.7			*FT5B(*2509.1Kb),*FT3B*(2528.1	⑩
g-AAT$_{DFM}$*-19-4*	*Glyma19g32540*	3	8.8	0.09	13.0				⑩
g-AAT$_{DFM}$*-19-5*	*Glyma19g33000*	3	12.4	0.23	33.8				⑧
g-AAT$_{DFM}$*-19-6*	*Glyma19g34740*	2	17.9	0.69	98.2	0.43	61.9	*Dt1*(*TFL1b*,326.8Kb), *Rsl 5-6*	⑤/⑦
g-AAT$_{DFM}$*-20-1*	*Glyma20g01960*	3	10.8	0.06	9.4				⑩
g-AAT$_{DFM}$*-20-2*	*Glyma20g23960*	3	7.4	0.08	11.4				⑩
g-AAT$_{DFM}$*-20-3*	*Glyma20g30150*	2	2.4	0.01	2.9				⑤
g-AAT$_{DFM}$*-20-4*	*Glyma20g31060*	3	3.3	0.03	4.6			*GIGANTEA*(78.9Kb)	⑩
g-AAT$_{DFM}$*-20-5*	*Glyma20g34860*	4	8.2	0.13	18.4				⑥
g-AAT$_{DFM}$*-20-6*	*Glyma20g36400*	2	14.2	0.22	34.1				⑩
Total	129	362(2.81)		41.54		28.86	28(22)		157

Note: Gene code: for example, *g-AAT*$_{DFM}$*-01-1*, where *AAT*$_{DFM}$ means DFM required accumulative active temperature, -01 represents chromosome 1, and -1 represents its order on the chromosome according to its physical position. The position corresponds to the Williams 82 reference genome version 1 (Wm82.a1).

Reported QTL, the QTL name is simplified; for example, *Rp1-3* represents reproductive period *1-3* and *Rsl7-1* represents reproductive stage length *7-1*. The same applies below. Gene group: Gene Ontology groups (please see the notes of Table S2 for details).

Table S8 The summary of gene–allele systems of six DSF- and DFM-related traits identified from GASM-RTM-GWAS in the WSGP.

Gene	GASM	Start position	End position	Allele no.	DSF gene code	ADL_{DSF} gene code	AAT_{DSF} gene code	DFM gene code	ADL_{DFM} gene code	AAT_{DFM} gene code	Gene group
Glyma01g01950	1_1521070_Block_1_1520440_1523995	1520440	1523995	2	/	g-ADL_{DSF}-01-1	/	/	/	/	⑩
Glyma01g02525	Block_1_2038299_2054214	2038299	2054214	4	/	/	/	/	g-ADL_{DFM}-01-1	/	⑤/⑦/⑨
Glyma01g02580	1_2122200_Block_1_2119857_2122838	2119857	2122838	2	/	/	g-AAT_{DSF}-01-1	/	/	/	⑥
Glyma01g02780	1_2278486_Block_1_2274286_2279870	2274286	2279870	2	/	/	/	g-DFM-01-1	/	/	⑧
Glyma01g03040	1_2531683_Block_1_2530563_2537849	2530563	2537849	2	g-DSF-01-1	/	/	/	/	/	⑧
Glyma01g04100	1_3567141_Block_1_3565110_3567816	3565110	3567816	2	/	g-ADL_{DSF}-01-2	/	/	/	/	⑨
Glyma01g04515	1_4094173_Block_1_4094046_4096371	4094046	4096371	2	/	/	/	/	/	g-AAT_{DFM}-01-1	⑩
Glyma01g06270	Block_1_6353279_6357024	6353279	6357024	3	/	/	g-AAT_{DSF}-01-2	/	/	/	⑩
Glyma01g22830	1_28986451_Block_1_28986403_28988037	28986403	28988037	2	g-DSF-01-2	g-ADL_{DSF}-01-3	g-AAT_{DSF}-01-3	/	/	/	⑤
Glyma01g28850	Block_1_38758938_38772979	38758938	38772979	2	/	/	/	/	/	g-AAT_{DFM}-01-2	⑩
Glyma01g33986	1_46305785_Block_1_46305208_46307924	46305208	46307924	2	/	/	/	/	g-ADL_{DFM}-01-2	/	②/⑧
Glyma01g34630	Block_1_47059947_47069275	47059947	47069275	2	g-DSF-01-3	/	/	/	/	/	⑧
Glyma01g35820	1_48322127_Block_1_48321787_48329293	48321787	48329293	2	g-DSF-01-4	/	/	/	/	/	⑥
Glyma01g36545	Block_1_49022249_49027494	49022249	49027494	3	/	/	/	/	g-ADL_{DFM}-01-3	/	⑩
Glyma01g40930	1_52587720_Block_1_52587536_52590299	52587536	52590299	2	/	g-ADL_{DSF}-01-4	/	/	/	/	⑥
Glyma01g41520	1_52993005_Block_1_52991945_52993448	52991945	52993448	2	g-DSF-01-5	/	/	/	/	/	⑤/⑥/⑦
Glyma01g43400	Block_1_54425016_54426043	54425016	54426043	4	g-DSF-01-6	/	/	/	/	/	⑩
Glyma01g43900	Block_1_54755183_54767332	54755183	54767332	3	/	/	/	/	/	g-AAT_{DFM}-01-3	⑥/⑧
Glyma02g00371	Block_2_156925_162109	156925	162109	4	/	g-ADL_{DSF}-02-1	/	g-DFM-02-1	g-ADL_{DFM}-02-1	g-AAT_{DFM}-02-1	⑧
Glyma02g01540	2_1101114_Block_2_1100819_1110176	1100819	1110176	2	/	/	/	/	/	g-AAT_{DFM}-02-2	④/⑤/⑥
Glyma02g03670	2_2892277_Block_2_2890415_2895159	2890415	2895159	2	/	/	/	g-DFM-02-2	/	/	①/⑧/⑨
Glyma02g03920	2_3133080_Block_2_3130868_3137826	3130868	3137826	2	g-DSF-02-1	/	g-AAT_{DSF}-02-1	/	/	/	⑩
Glyma02g04010	2_3224287_Block_2_3220822_3226910	3220822	3226910	2	g-DSF-02-2	/	/	/	/	/	⑧
Glyma02g04190	Block_2_3380678_3390117	3380678	3390117	3	g-DSF-02-3	g-ADL_{DSF}-02-2	g-AAT_{DSF}-02-2	/	/	/	⑤/⑧
Glyma02g05550	Block_2_4438431_4443610	4438431	4443610	5	/	/	/	/	/	g-AAT_{DFM}-02-3	⑧
Glyma02g06420	Block_2_5115581_5120689	5115581	5120689	3	g-DSF-02-4	/	/	/	/	/	⑤/⑧
Glyma02g06530	2_5207131_Block_2_5206527_5208734	5206527	5208734	2	/	/	g-AAT_{DSF}-02-3	/	/	/	⑤
Glyma02g06730	Block_2_5373078_5381786	5373078	5381786	4	/	g-ADL_{DSF}-02-3	/	/	/	g-AAT_{DFM}-02-4	①/⑤
Glyma02g06841	2_5498988_Block_2_5497451_5499522	5497451	5499522	2	/	/	/	/	/	g-AAT_{DFM}-02-5	⑩
Glyma02g07010	Block_2_5618681_5620028	5618681	5620028	4	g-DSF-02-5	/	/	/	/	/	⑦
Glyma02g07270	Block_2_5772064_5783545	5772064	5783545	3	/	/	/	g-DFM-02-3	/	/	②/④
Glyma02g07640	2_6025291_Block_2_6013205_6030334	6013205	6030334	2	/	/	g-AAT_{DSF}-02-4	/	/	/	⑧
Glyma02g08091	2_6338428_Block_2_6336129_6338496	6336129	6338496	2	/	/	/	/	g-ADL_{DFM}-02-2	/	⑩
Glyma02g08430	Block_2_6606530_6610284	6606530	6610284	3	/	/	/	/	/	g-AAT_{DFM}-02-6	⑥
Glyma02g08620	Block_2_6704552_6712419	6704552	6712419	3	/	g-ADL_{DSF}-02-4	/	/	g-ADL_{DFM}-02-3	/	⑩
Glyma02g08790	2_6814590_Block_2_6808506_6815124	6808506	6815124	2	g-DSF-02-6	/	/	/	/	/	①/②
Glyma02g13165	Block_2_11380636_11383227	11380636	11383227	5	/	g-ADL_{DSF}-02-5	/	/	/	/	⑩
Glyma02g13655	Block_2_11962235_11969162	11962235	11969162	2	/	g-ADL_{DSF}-02-6	/	/	/	/	⑧
Glyma02g15470	Block_2_13966704_13982279	13966704	13982279	3	/	/	/	/	/	g-AAT_{DFM}-02-7	⑩
Glyma02g15561	Block_2_14038090_14039926	14038090	14039926	4	/		/g-AAT_{DSF}-02-5	/	/	/	⑩
Glyma02g16560	2_14939413_Block_2_14938939_14940735	14938939	14940735	2	/	g-ADL_{DSF}-02-7	/	/	/	/	⑩
Glyma02g19000	Block_2_17800639_17804109	17800639	17804109	2	/	/	/	/		0g-AAT_{DFM}-02-8	⑤/⑦
Glyma02g26620	2_27546010_Block_2_27538568_27547029	27538568	27547029	2	g-DSF-02-7	/	/	/	/	/	①
Glyma02g31295	Block_2_34032288_34046647	34032288	34046647	3	/	/	/	/	g-ADL_{DFM}-02-4	/	⑤
Glyma02g36665	Block_2_42109438_42110485	42109438	42110485	2	/	/	/	/	g-ADL_{DFM}-02-5	/	⑩
Glyma02g37350	Block_2_42688765_42695750	42688765	42695750	3	g-DSF-02-8	/	g-AAT_{DSF}-02-6	/	/	/	⑦
Glyma02g38790	2_44148112_Block_2_44138604_44149762	44138604	44149762	2	g-DSF-02-9	/	/	/	/	/	⑩
Glyma02g39495	Block_2_44802132_44811779	44802132	44811779	3	/	g-ADL_{DSF}-02-8	/	/	/	/	④/⑧
Glyma02g40650	2_45897419_Block_2_45888235_45899367	45888235	45899367	2	/	/	/	g-DFM-02-4	/	/	①/⑤/⑦
Glyma02g41040	Block_2_46213331_46222911	46213331	46222911	3	/	/	/	g-DFM-02-5	/	/	⑦
Glyma02g42290	2_47355073_Block_2_47353533_47359138	47353533	47359138	2	/	/	/	/	/	g-AAT_{DFM}-02-9	⑦
Glyma02g43025	2_47916058_Block_2_47910442_47919144	47910442	47919144	2	/	/	/	/	g-ADL_{DFM}-02-6	/	⑧/⑨
Glyma02g45630	2_49820623_Block_2_49816299_49823607	49816299	49823607	2	g-DSF-02-10	/	/	/	/	/	⑥/⑧
Glyma02g45790	Block_2_49937008_49942753	49937008	49942753	3	/	/	g-AAT_{DSF}-02-7	/	/	/	⑧
Glyma02g45960	Block_2_50058602_50081372	50058602	50081372	2	/	g-ADL_{DSF}-02-9	/	/	/	/	⑤
Glyma02g46400	2_50368934_Block_2_50368276_50370502	50368276	50370502	2	/	/	/	/	/	g-AAT_{DFM}-02-10	⑩
Glyma02g46800	2_50645254_Block_2_50644219_50650679	50644219	50650679	2	/	/	g-AAT_{DSF}-02-8	/	/	/	⑥
Glyma02g46810	2_50660676_Block_2_50659738_50666155	50659738	50666155	2	/	/	/	/	g-ADL_{DFM}-02-7	/	⑥
Glyma02g48010	2_51400285_Block_2_51394513_51401344	51394513	51401344	2	g-DSF-02-11	/	g-AAT_{DSF}-02-9	/	/	/	⑩

Glyma03g02390	3_2153744_Block_3_2139071_2153795	2139071	2153795	2	/	g-ADL$_{DSF}$-03-1	/	/	/	/	⑥
Glyma03g02500	Block_3_2276240_2279952	2276240	2279952	3	/	/	/	/	g-ADL$_{DFM}$-03-1	/	⑤
Glyma03g02940	Block_3_2757705_2762734	2757705	2762734	6	g-DSF-03-1	/	/	g-DFM-03-1	g-ADL$_{DFM}$-03-2	/	⑩
Glyma03g03120	Block_3_2912959_2933829	2912959	2933829	5	g-DSF-03-2	/	/	/	/	/	①
Glyma03g04590	Block_3_4757597_4764753	4757597	4764753	5	/	/	/	/	g-ADL$_{DFM}$-03-3	/	⑦
Glyma03g04720	Block_3_4917301_4924329	4917301	4924329	6	g-DSF-03-3	/	/	/	/	/	⑩
Glyma03g05120	3_5408085_Block_3_5405047_5409407	5405047	5409407	2	/	/	/	/	/	g-AAT$_{DFM}$-03-1	⑩
Glyma03g06440	Block_3_6712344_6715034	6712344	6715034	5	/	/	g-AAT$_{DSF}$-03-1	/	/	/	⑤
Glyma03g06483	Block_3_6755858_6757490	6755858	6757490	3	/	/	/	g-DFM-03-2	g-ADL$_{DFM}$-03-4	/	④/⑦/⑥
Glyma03g15870	3_20197046_Block_3_20197005_20200237	20197005	20200237	2	/	g-ADL$_{DSF}$-03-2	/	/	/	/	⑤
Glyma03g18725	Block_3_23895580_23919054	23895580	23919054	5	/	/	/	/	/	g-AAT$_{DFM}$-03-2	⑩
Glyma03g20420	3_25948733_Block_3_25943786_25950785	25943786	25950785	2	/	/	/	/	/	g-AAT$_{DFM}$-03-3	⑥
Glyma03g24890	3_31811597_Block_3_31806821_31812814	31806821	31812814	2	/	/	/	g-DFM-03-3	/	/	⑩
Glyma03g25145	Block_3_32117666_32139732	32117666	32139732	4	/	/	/	g-DFM-03-4	/	/	①/⑥
Glyma03g27150	Block_3_34672940_34684987	34672940	34684987	3	/	g-ADL$_{DSF}$-03-3	/	/	/	/	⑩
Glyma03g27290	3_34979145_Block_3_34977900_34980034	34977900	34980034	2	/	/	/	/	g-ADL$_{DFM}$-03-5	/	⑦
Glyma03g27970	Block_3_35773797_35777130	35773797	35777130	4	g-DSF-03-4	g-ADL$_{DSF}$-03-4	/	/	/	/	③/⑦
Glyma03g29010	Block_3_36959429_36972106	36959429	36972106	4	/	/	/	/	/	g-AAT$_{DFM}$-03-4	⑥
Glyma03g29060	Block_3_37006191_37009001	37006191	37009001	3	/	/	/	/	/	g-AAT$_{DFM}$-03-5	⑩
Glyma03g29070	Block_3_37017711_37021248	37017711	37021248	3	/	g-ADL$_{DSF}$-03-5	/	/	/	/	⑤
Glyma03g29230	Block_3_37184174_37222229	37184174	37222229	4	/	/	g-AAT$_{DSF}$-03-2	/	g-ADL$_{DFM}$-03-6	/	⑧
Glyma03g33000	Block_3_40689640_40693587	40689640	40693587	4	/	/	g-AAT$_{DSF}$-03-3	/	/	/	⑩
Glyma03g33470	3_41029045_Block_3_41027184_41030475	41027184	41030475	2	/	g-ADL$_{DSF}$-03-6	/	/	/	/	①/⑤
Glyma03g34900	3_42205000_Block_3_42204661_42207243	42204661	42207243	2	/	/	/	/	/	g-AAT$_{DFM}$-03-6	③/⑤
Glyma03g38171	3_44572005_Block_3_44569717_44573490	44569717	44573490	2	/	/	/	/	g-ADL$_{DFM}$-03-7	/	⑩
Glyma03g39050	3_45279841_Block_3_45279743_45281178	45279743	45281178	2	/	g-ADL$_{DSF}$-03-7	/	/	/	/	⑩
Glyma03g39850	Block_3_45790397_45794969	45790397	45794969	3	/	/	/	/	g-ADL$_{DFM}$-03-8	/	⑥
Glyma03g39870	Block_3_45803748_45806283	45803748	45806283	3	/	/	/	g-DFM-03-5	/	/	⑩
Glyma03g40780	Block_3_46431667_46441697	46431667	46441697	3	g-DSF-03-5	/	/	/	/	/	③/⑧
Glyma03g41200	Block_3_46724556_46730266	46724556	46730266	3	/	/	/	g-DFM-03-6	/	/	⑧
Glyma03g41280	Block_3_46774543_46779426	46774543	46779426	3	/	/	/	/	g-ADL$_{DFM}$-03-9	/	⑩
Glyma03g41290	Block_3_46782386_46810161	46782386	46810161	2	g-DSF-03-6	/	/	/	/	/	⑧
Glyma03g41320	3_46833583_Block_3_46831055_46834070	46831055	46834070	2	/	g-ADL$_{DSF}$-03-8	/	/	/	/	⑧
Glyma03g41360	3_46881880_Block_3_46880973_46883067	46880973	46883067	2	/	/	/	/	g-ADL$_{DFM}$-03-10	/	⑦/⑧
Glyma03g41520	3_46951037_Block_3_46950548_46951293	46950548	46951293	2	/	/	g-AAT$_{DSF}$-03-4	/	/	/	⑩
Glyma03g42060	Block_3_47371310_47372914	47371310	47372914	3	/	/	g-AAT$_{DSF}$-03-5	/	/	/	⑩
Glyma04g01990	4_1360177_Block_4_1360026_1372881	1360026	1372881	2	/	/	/	/	g-ADL$_{DFM}$-04-1	/	⑤
Glyma04g02330	Block_4_1605016_1611651	1605016	1611651	4	/	/	/	g-DFM-04-1	/	/	①
Glyma04g02740	Block_4_1934014_1941547	1934014	1941547	3	/	/	g-AAT$_{DSF}$-04-1	/	/	/	⑦
Glyma04g02850	Block_4_2059088_2063081	2059088	2063081	4	/	/	g-AAT$_{DSF}$-04-2	/	/	/	⑤/⑧
Glyma04g02980	Block_4_2155604_2159914	2155604	2159914	3	/	/	/	/	/	g-AAT$_{DFM}$-04-1	①/⑤
Glyma04g04810	Block_4_3567302_3577686	3567302	3577686	3	/	/	/	g-DFM-04-2	/	/	⑥
Glyma04g05471	Block_4_4135841_4138280	4135841	4138280	5	/	/	/	/	g-ADL$_{DFM}$-04-2	/	⑧
Glyma04g05600	4_4269464_Block_4_4264746_4269923	4264746	4269923	2	/	/	g-AAT$_{DSF}$-04-3	/	/	/	①/⑧
Glyma04g06240	Block_4_4774049_4775966	4774049	4775966	4	/	/	/	/	/	g-AAT$_{DFM}$-04-2	⑤/⑦
Glyma04g06600	Block_4_5049829_5055377	5049829	5055377	4	/	g-ADL$_{DSF}$-04-1	/	/	/	/	⑩
Glyma04g06940	4_5366405_Block_4_5365795_5367560	5365795	5367560	2	/	/	/	g-DFM-04-3	/	/	⑩
Glyma04g07160	4_5568883_Block_4_5568471_5571119	5568471	5571119	2	/	g-ADL$_{DSF}$-04-2	/	/	/	/	⑤/⑧
Glyma04g07430	4_5773213_Block_4_5770334_5773812	5770334	5773812	2	g-DSF-04-1	/	/	/	/	/	⑧
Glyma04g09910	4_8198712_Block_4_8197404_8199603	8197404	8199603	2	g-DSF-04-2	/	/	/	/	/	⑦
Glyma04g09950	4_8241411_Block_4_8238777_8246273	8238777	8246273	2	/	/	/	/	g-ADL$_{DFM}$-04-3	/	⑨
Glyma04g10050	Block_4_8302112_8307589	8302112	8307589	4	/	/	/	/	g-ADL$_{DFM}$-04-4	/	⑥/⑦
Glyma04g10451	Block_4_8670881_8701308	8670881	8701308	3	/	/	/	g-DFM-04-4	/	/	⑥
Glyma04g12320	4_11341353_Block_4_11340795_11343215	11340795	11343215	2	/	/	/	/	g-ADL$_{DFM}$-04-5	/	⑤
Glyma04g13142	Block_4_12726785_12728920	12726785	12728920	3	/	/	/	g-DFM-04-5	/	/	⑩
Glyma04g16100	Block_4_17071886_17084681	17071886	17084681	6	/	/	/	/	/	g-AAT$_{DFM}$-04-3	①
Glyma04g16180	Block_4_17191541_17204756	17191541	17204756	4	/	g-ADL$_{DSF}$-04-3	/	/	/	/	②/⑤
Glyma04g20400	Block_4_22435029_22443669	22435029	22443669	4	/	/	/	/	/	g-AAT$_{DFM}$-04-4	⑩
Glyma04g33110	Block_4_38589698_38595505	38589698	38595505	3	/	/	/	/	g-ADL$_{DFM}$-04-6	/	②/⑤
Glyma04g35730	Block_4_42209590_42213796	42209590	42213796	4	/	/	g-AAT$_{DSF}$-04-4	/	/	/	⑥
Glyma04g36240	Block_4_42776832_42784326	42776832	42784326	3	g-DSF-04-3	/	g-AAT$_{DSF}$-04-5	/	/	/	⑩
Glyma04g36260	Block_4_42819644_42824277	42819644	42824277	3	/	/	/	/	g-ADL$_{DFM}$-04-7	/	⑧
Glyma04g36630	Block_4_43141886_43152420	43141886	43152420	2	/	/	g-AAT$_{DSF}$-04-6	/	/	/	①/④/⑦
Glyma04g36790	4_43304222_Block_4_43304149_43309436	43304149	43309436	2	/	/	/	/	/	g-AAT$_{DFM}$-04-5	⑦

Glyma04g37000	4_43472097_Block_4_43470306_43475797	43470306	43475797	2	/	/	/	*g-DFM-04-6*	/	/	⑦
Glyma04g38955	Block_4_45238405_45241750	45238405	45241750	3	/	/	/	/	/	*g-AAT$_{DFM}$-04-6*	⑤/⑥
Glyma04g39610	4_45798473_Block_4_45797339_45801559	45797339	45801559	2	*g-DSF-04-4*	/	/	/	/	/	⑥
Glyma04g40310	4_46417298_Block_4_46406328_46417822	46406328	46417822	2	/	/	/	/	/	*g-AAT$_{DFM}$-04-7*	①/⑤
Glyma04g41390	4_47233848_Block_4_47231017_47236260	47231017	47236260	2	/	*g-ADL$_{DSF}$-04-4*	/	/	/	/	④/⑤/⑥
Glyma04g42010	Block_4_47794226_47796648	47794226	47796648	3	/	/	/	/	/	*g-AAT$_{DFM}$-04-8*	⑨
Glyma04g42900	Block_4_48528145_48532416	48528145	48532416	3	/	/	/	/	*g-ADL$_{DFM}$-04-8*	/	⑥
Glyma04g42981	Block_4_48598361_48599194	48598361	48599194	2	/	/	*g-AAT$_{DSF}$-04-7*	/	/	/	⑩
Glyma04g43550	4_49093154_Block_4_49092402_49095573	49092402	49095573	2	/	*g-ADL$_{DSF}$-04-5*	/	/	/	/	⑥
Glyma05g01943	Block_5_1401783_1404122	1401783	1404122	3	/	/	/	/	/	*g-AAT$_{DFM}$-05-1*	⑩
Glyma05g02130	5_1554612_Block_5_1549339_1555419	1549339	1555419	2	/	/	/	*g-DFM-05-1*	/	/	⑧/⑨
Glyma05g02140	Block_5_1562541_1564269	1562541	1564269	3	*g-DSF-05-1*	/	/	/	/	/	⑩
Glyma05g02470	5_1819575_Block_5_1817624_1822457	1817624	1822457	2	/	/	*g-AAT$_{DSF}$-05-1*	/	/	/	⑥/⑧
Glyma05g02790	5_2133489_Block_5_2130631_2134436	2130631	2134436	2	*g-DSF-05-2*	/	/	/	/	/	⑥
Glyma05g04330	Block_5_3498691_3503345	3498691	3503345	3	/	/	/	*g-DFM-05-2*	/	/	⑩
Glyma05g04561	5_3704626_Block_5_3704617_3711041	3704617	3711041	2	/	/	/	/	*g-ADL$_{DFM}$-05-1*	*g-AAT$_{DFM}$-05-2*	⑩
Glyma05g08670	Block_5_8547050_8549538	8547050	8549538	3	/	/	/	*g-DFM-05-3*	/	/	⑩
Glyma05g09170	Block_5_8950179_8957086	8950179	8957086	2	/	/	/	*g-DFM-05-4*	/	/	⑩
Glyma05g22680	5_28029661_Block_5_28028771_28030131	28028771	28030131	2	/	/	/	/	*g-ADL$_{DFM}$-05-2*	/	⑩
Glyma05g23000	Block_5_28432571_28443362	28432571	28443362	3	/	/	/	*g-DFM-05-5*	/	/	⑩
Glyma05g23731	Block_5_29497446_29511105	29497446	29511105	3	/	*g-ADL$_{DSF}$-05-1*	/	/	/	/	⑧
Glyma05g24580	5_30745874_Block_5_30741313_30746355	30741313	30746355	2	/	/	/	/	*g-ADL$_{DFM}$-05-3*	/	⑩
Glyma05g25485	5_31581611_Block_5_31577344_31582079	31577344	31582079	2	/	/	/	/	/	*g-AAT$_{DFM}$-05-3*	⑩
Glyma05g26050	5_32061707_Block_5_32060512_32062911	32060512	32062911	2	/	*g-ADL$_{DSF}$-05-2*	/	/	/	/	⑩
Glyma05g26620	5_32545029_Block_5_32544734_32549138	32544734	32549138	2	/	/	/	*g-DFM-05-6*	/	*g-AAT$_{DFM}$-05-4*	④/⑧
Glyma05g27870	Block_5_33722834_33724150	33722834	33724150	2	/	*g-ADL$_{DSF}$-05-3*	/	/	/	/	⑥/⑧
Glyma05g29190	Block_5_34857613_34860916	34857613	34860916	5	/	*g-ADL$_{DSF}$-05-4*	/	/	/	/	⑥/⑧
Glyma05g29200	5_34871857_Block_5_34870284_34874742	34870284	34874742	2	/	/	/	*g-DFM-05-7*	/	/	③/⑦/⑧
Glyma05g29880	Block_5_35346130_35350415	35346130	35350415	2	/	/	/	/	*g-ADL$_{DFM}$-05-4*	/	⑩
Glyma05g31250	Block_5_36389171_36392674	36389171	36392674	3	*g-DSF-05-3*	/	*g-AAT$_{DSF}$-05-2*	/	/	/	③
Glyma05g32030	5_37027716_Block_5_37026301_37031440	37026301	37031440	2	*g-DSF-05-4*	/	/	/	/	/	⑧
Glyma05g35310	5_39378064_Block_5_39377703_39385245	39377703	39385245	2	/	/	/	/	*g-ADL$_{DFM}$-05-5*	/	⑥
Glyma05g37380	5_40984736_Block_5_40984367_40994598	40984367	40994598	2	/	*g-ADL$_{DSF}$-05-5*	/	/	/	/	⑧
Glyma05g38075	Block_5_41501804_41509361	41501804	41509361	3	/	/	/	*g-DFM-05-8*	/	/	⑩
Glyma0006s50	Block_6_1758_15096	1758	15096	2	/	/	/	*g-DFM-06-1*	/	/	④/⑦
Glyma06g03470	6_2424859_Block_6_2423386_2428029	2423386	2428029	2	*g-DSF-06-1*	/	/	/	*g-ADL$_{DFM}$-06-1*	/	⑤
Glyma06g04140	6_2882783_Block_6_2881673_2883094	2881673	2883094	2	/	/	*g-AAT$_{DSF}$-06-1*	/	/	/	⑩
Glyma06g05870	Block_6_4199931_4201104	4199931	4201104	3	/	/	/	*g-DFM-06-2*	/	/	⑩
Glyma06g05890	Block_6_4207295_4216123	4207295	4216123	2	/	/	/	/	*g-ADL$_{DFM}$-06-2*	/	⑥
Glyma06g10320	Block_6_7790944_7826996	7790944	7826996	5	/	*g-ADL$_{DSF}$-06-1*	/	/	/	/	⑦
Glyma06g11120	6_8479122_Block_6_8467947_8483520	8467947	8483520	2	/	/	/	/	/	*g-AAT$_{DFM}$-06-1*	⑦/⑧
Glyma06g11370	Block_6_8719520_8730703	8719520	8730703	4	/	/	*g-AAT$_{DSF}$-06-2*	/	/	/	⑩
Glyma06g13320	Block_6_10466896_10472898	10466896	10472898	2	*g-DSF-06-2*	/	/	/	/	/	⑧
Glyma06g13985	Block_6_11053511_11054273	11053511	11054273	3	/	/	/	/	*g-ADL$_{DFM}$-06-3*	/	⑩
Glyma06g15020	6_11809614_Block_6_11804605_11814857	11804605	11814857	2	*g-DSF-06-3*	/	/	/	/	/	⑥
Glyma06g15390	6_12135265_Block_6_12134893_12135784	12134893	12135784	2	/	/	/	/	/	*g-AAT$_{DFM}$-06-2*	⑩
Glyma06g15890	6_12501439_Block_6_12500229_12501933	12500229	12501933	2	/	/	/	/	*g-ADL$_{DFM}$-06-4*	/	②/⑧
Glyma06g16330	Block_6_12817416_12825189	12817416	12825189	4	*g-DSF-06-4*	/	/	/	/	/	⑩
Glyma06g16930	Block_6_13307622_13311321	13307622	13311321	3	/	/	*g-AAT$_{DSF}$-06-3*	/	/	/	⑧
Glyma06g17390	Block_6_13703318_13713477	13703318	13713477	3	/	/	/	/	/	*g-AAT$_{DFM}$-06-3*	⑩
Glyma06g17451	6_13804692_Block_6_13802452_13808691	13802452	13808691	2	/	/	/	/	/	*g-AAT$_{DFM}$-06-4*	⑩
Glyma06g18216	Block_6_14541262_14603971	14541262	14603971	4	/	/	/	*g-DFM-06-3*	/	/	①/④
Glyma06g18580	6_14868170_Block_6_14867697_14876182	14867697	14876182	2	/	*g-ADL$_{DSF}$-06-2*	/	/	/	/	①
Glyma06g19380	6_15600413_Block_6_15599391_15600917	15599391	15600917	2	/	/	/	/	/	*g-AAT$_{DFM}$-06-5*	⑩
Glyma06g19480	6_15738284_Block_6_15737043_15739732	15737043	15739732	2	/	/	/	/	*g-ADL$_{DFM}$-06-5*	*g-AAT$_{DFM}$-06-6*	②/⑦/⑨
Glyma06g19651	6_15963037_Block_6_15962494_15964022	15962494	15964022	2	/	/	*g-AAT$_{DSF}$-06-4*	/	/	/	⑧
Glyma06g20950	6_17335692_Block_6_17335571_17338829	17335571	17338829	2	/	/	*g-AAT$_{DSF}$-06-5*	/	/	/	⑤
Glyma06g23580	Block_6_20904283_20914087	20904283	20914087	4	*g-DSF-06-5*	*g-ADL$_{DSF}$-06-3*	*g-AAT$_{DSF}$-06-6*	/	*g-ADL$_{DFM}$-06-6*	/	⑩
Glyma06g32870	Block_6_33837618_33843206	33837618	33843206	4	/	*g-ADL$_{DSF}$-06-4*	*g-AAT$_{DSF}$-06-7*	/	/	/	⑩
Glyma06g36380	6_38548244_Block_6_38545298_38554455	38545298	38554455	2	*g-DSF-06-6*	/	*g-AAT$_{DSF}$-06-8*	/	/	/	④
Glyma06g40370	Block_6_43518780_43522569	43518780	43522569	4	*g-DSF-06-7*	/	/	/	/	/	⑧
Glyma06g40560	Block_6_43723614_43727398	43723614	43727398	3	/	/	/	/	/	*g-AAT$_{DFM}$-06-7*	⑧
Glyma06g40670	Block_6_43829510_43834104	43829510	43834104	4	/	/	*g-AAT$_{DSF}$-06-9*	*g-DFM-06-4*	*g-ADL$_{DFM}$-06-7*	/	⑧
Glyma06g42730	6_46065201_Block_6_46063689_46069098	46063689	46069098	2	/	/	*g-AAT$_{DSF}$-06-10*	/	/	/	⑥

Glyma06g43951	6_46933905_Block_6_46932145_46936357	46932145	46936357	2	/	g-ADL$_{DSF}$-06-5	/	/	/	/	①/⑧
Glyma06g44630	6_47495235_Block_6_47493702_47498334	47493702	47498334	2	/	g-ADL$_{DSF}$-06-6	/	/	/	/	⑤
Glyma06g45220	6_48032833_Block_6_48032729_48035503	48032729	48035503	2	/	/	g-AAT$_{DSF}$-06-11	/	/	/	⑩
Glyma06g45450	Block_6_48159801_48166369	48159801	48166369	4	/	/	/	g-DFM-06-5	/	/	⑦
Glyma06g46150	Block_6_48819539_48826307	48819539	48826307	3	/	/	/	g-DFM-06-6	/	/	⑥
Glyma06g46781	Block_6_49348476_49354343	49348476	49354343	3	g-DSF-06-8	/	/	/	/	/	⑨
Glyma06g47590	Block_6_49984461_49991248	49984461	49991248	4	g-DSF-06-9		0g-AAT$_{DSF}$-06-12	/	/	/	⑩
Glyma06g48150	Block_6_50439510_50444604	50439510	50444604	3	/	/	/	g-DFM-06-7	/	/	⑤
Glyma06g48393	Block_6_50647868_50650673	50647868	50650673	3	/	/	/	/	/	g-AAT$_{DFM}$-06-8	⑩
Glyma07g00520	Block_7_243867_247741	243867	247741	4	/	/	g-AAT$_{DSF}$-07-1	/	/	/	③/⑤/⑦/⑧
Glyma07g03005	7_2054346_Block_7_2052773_2062003	2052773	2062003	2	/	/	/	g-DFM-07-1	/	/	⑩
Glyma07g04800	7_3540547_Block_7_3540227_3541661	3540227	3541661	2	/	/	/	/	/	g-AAT$_{DFM}$-07-1	⑤/⑧
Glyma07g05290	7_3946123_Block_7_3942165_3947296	3942165	3947296	2	/	g-ADL$_{DSF}$-07-1	/	/	g-ADL$_{DFM}$-07-1	/	⑩
Glyma07g06420	Block_7_5157818_5165072	5157818	5165072	4	/	/	/	/	/	g-AAT$_{DFM}$-07-2	②
Glyma07g07600	7_6293114_Block_7_6290183_6297333	6290183	6297333	2	/	/	/	g-DFM-07-2	/	/	⑧
Glyma07g08214	7_6826804_Block_7_6824048_6827468	6824048	6827468	2	g-DSF-07-1	/	/	/	/	/	⑧
Glyma07g09170	Block_7_7646374_7655460	7646374	7655460	8	g-DSF-07-2	/	g-AAT$_{DSF}$-07-2	/	/	/	⑩
Glyma07g09420	Block_7_7852546_7856950	7852546	7856950	5	/	/	/	g-DFM-07-3	g-ADL$_{DFM}$-07-2	/	⑧
Glyma07g09860	7_8318354_Block_7_8314303_8320645	8314303	8320645	2	g-DSF-07-3	/	/	g-DFM-07-4	/	/	⑧
Glyma07g10060	Block_7_8426231_8428377	8426231	8428377	5	/	/	g-AAT$_{DSF}$-07-3	/	/	/	⑩
Glyma07g10541	Block_7_8802805_8814519	8802805	8814519	3	/	/	/	/	g-ADL$_{DFM}$-07-3	g-AAT$_{DFM}$-07-3	⑧
Glyma07g10621	Block_7_8869326_8879181	8869326	8879181	6	/	/	/	/	g-ADL$_{DFM}$-07-4	/	⑧
Glyma07g11091	Block_7_9305397_9308161	9305397	9308161	3	/	/	/	/	g-ADL$_{DFM}$-07-5	/	⑩
Glyma07g11670	Block_7_9867874_9880578	9867874	9880578	3	g-DSF-07-4	/	/	/	/	/	⑧
Glyma07g13710	7_12707963_Block_7_12705981_12708326	12705981	12708326	2	g-DSF-07-5	/	/	/	/	/	⑩
Glyma07g14242	7_13599508_Block_7_13594198_13602239	13594198	13602239	2	g-DSF-07-6	/	/	/	/	/	⑩
Glyma07g14870	Block_7_14709171_14713495	14709171	14713495	3	/	/	g-AAT$_{DSF}$-07-4	/	/	/	⑤
Glyma07g15930	Block_7_15649032_15653633	15649032	15653633	4	g-DSF-07-7	/	/	/	/	/	⑥
Glyma07g17620	7_17373271_Block_7_17372764_17375445	17372764	17375445	2	/	/	/	/	/	g-AAT$_{DFM}$-07-4	⑩
Glyma07g18060	Block_7_17821300_17825005	17821300	17825005	2	/	/	/	g-DFM-07-5	/	/	⑤/⑧
Glyma07g20421	7_20623711_Block_7_20622640_20624725	20622640	20624725	2	g-DSF-07-8	/	/	/	/	/	⑩
Glyma07g29650	Block_7_34640179_34652411	34640179	34652411	2	g-DSF-07-9	/	/	/	g-ADL$_{DFM}$-07-6	g-AAT$_{DFM}$-07-5	⑨
Glyma07g30140	7_35187021_Block_7_35186721_35192803	35186721	35192803	2	/	/	g-AAT$_{DSF}$-07-5	/	/	/	⑤
Glyma07g33190	7_38111183_Block_7_38110940_38111341	38110940	38111341	2	g-DSF-07-10	/	/	/	/	/	⑩
Glyma07g33880	7_38814683_Block_7_38814437_38816833	38814437	38816833	2	/	/	/	g-DFM-07-6	/	/	⑦
Glyma07g34820	7_39871777_Block_7_39859215_39875837	39859215	39875837	2	/	/	/	/	/	g-AAT$_{DFM}$-07-6	⑩
Glyma07g36065	Block_7_41437437_41493796	41437437	41493796	3	/	/	g-AAT$_{DSF}$-07-6	/	/	/	⑥/⑧
Glyma07g37580	7_42582379_Block_7_42582011_42582424	42582011	42582424	2	g-DSF-07-11	/	/	g-DFM-07-7	/	/	⑩
Glyma07g39110	Block_7_43693801_43696239	43693801	43696239	3	/	/	/	/	g-ADL$_{DFM}$-07-7	/	⑩
Glyma07g40260	7_44541413_Block_7_44537248_44546138	44537248	44546138	2	/	/	/	g-DFM-07-8	g-ADL$_{DFM}$-07-8	g-AAT$_{DFM}$-07-7	⑩
Glyma08g02210	Block_8_1535119_1544710	1535119	1544710	3	/	/	/	/	g-ADL$_{DFM}$-08-1	/	⑥/⑦
Glyma08g03210	Block_8_2261024_2271853	2261024	2271853	4	g-DSF-08-1	g-ADL$_{DSF}$-08-1	g-AAT$_{DSF}$-08-1	/	/	/	⑨
Glyma08g04320	Block_8_3033938_3038711	3033938	3038711	3	/	/	g-AAT$_{DSF}$-08-2	/	/	/	⑩
Glyma08g04620	8_3293055_Block_8_3292737_3296203	3292737	3296203	2	/	/	/	g-DFM-08-1	/	/	⑤/⑧
Glyma08g07050	8_5068701_Block_8_5068280_5070727	5068280	5070727	2	/	/	/	/	/	g-AAT$_{DFM}$-08-1	⑧
Glyma08g09191	Block_8_6571150_6576254	6571150	6576254	2	/	g-ADL$_{DSF}$-08-2	/	/	/	/	⑩
Glyma08g10960	Block_8_8000613_8002682	8000613	8002682	2	/	/	/	g-DFM-08-2	/	/	⑩
Glyma08g11480	Block_8_8346503_8349314	8346503	8349314	5	/	/	/	/	/	g-AAT$_{DFM}$-08-2	④/⑧
Glyma08g12350	Block_8_9027058_9030678	9027058	9030678	3	/	g-ADL$_{DSF}$-08-3	/	/	/	/	⑥/⑧
Glyma08g13330	Block_8_9749492_9754179	9749492	9754179	3	/	/	g-AAT$_{DSF}$-08-3	/	/	/	⑤/⑧
Glyma08g14150	Block_8_10274335_10274917	10274335	10274917	3	/	/	g-AAT$_{DSF}$-08-4	/	/	/	⑩
Glyma08g15050	8_10932604_Block_8_10932560_10935883	10932560	10935883	2	/	/	/	/	/	g-AAT$_{DFM}$-08-3	⑤/⑦
Glyma08g15181	Block_8_10987261_11012066	10987261	11012066	3	g-DSF-08-2	/	/	/	/	/	⑩
Glyma08g15250	Block_8_11053202_11058521	11053202	11058521	3	/	/	/	g-DFM-08-3	/	/	⑥
Glyma08g15400	8_11174733_Block_8_11172365_11176099	11172365	11176099	2	/	/	/	/	g-ADL$_{DFM}$-08-2	g-AAT$_{DFM}$-08-4	⑩
Glyma08g15870	8_11557087_Block_8_11557045_11562288	11557045	11562288	2	/	/	/	g-DFM-08-4	g-ADL$_{DFM}$-08-3	g-AAT$_{DFM}$-08-5	⑩
Glyma08g19780	8_14934134_Block_8_14933155_14936631	14933155	14936631	2	/	/	/	g-DFM-08-5	/	/	⑩
Glyma08g20140	Block_8_15201767_15207473	15201767	15207473	4	/	/	g-AAT$_{DSF}$-08-5	/	/	/	④/⑤
Glyma08g20625	8_15627259_Block_8_15626690_15632810	15626690	15632810	2	/	/	/	g-DFM-08-6	/	/	⑧
Glyma08g23480	8_17912868_Block_8_17912330_17917847	17912330	17917847	2	/	/	/	g-DFM-08-7	/	/	⑨
Glyma08g23740	Block_8_18086610_18090421	18086610	18090421	2	/	/	/	/	g-ADL$_{DFM}$-08-4	/	⑥
Glyma08g24100	Block_8_18313700_18317765	18313700	18317765	4	/	/	/	/	/	g-AAT$_{DFM}$-08-6	⑩
Glyma08g24630	Block_8_18774407_18802218	18774407	18802218	3	/	g-ADL$_{DSF}$-08-4	/	/	/	/	⑩
Glyma08g27633	Block_8_21938713_21952487	21938713	21952487	4	/	g-ADL$_{DSF}$-08-5	/	/	/	/	⑦

Glyma08g28765	8_23143299_Block_8_23141865_23143429	23141865	23143429	2	/	/	/	*g-DFM-08-8*	/	/	⑩
Glyma08g28890	8_23224982_Block_8_23224932_23235003	23224932	23235003	2	/	/	*g-AAT*$_{DSF}$*-08-6*	/	/	/	①/⑦
Glyma08g28920	Block_8_23268446_23275603	23268446	23275603	3	/	*g-ADL*$_{DSF}$*-08-6*	/	/	/	/	⑤/⑦
Glyma08g40530	8_40251509_Block_8_40250975_40274353	40250975	40274353	2	/	/	/	*g-DFM-08-9*	/	/	⑥
Glyma08g43140	8_43004125_Block_8_42997269_43005846	42997269	43005846	2	/	/	*g-AAT*$_{DSF}$*-08-7*	/	/	/	⑩
Glyma08g43390	Block_8_43227801_43230035	43227801	43230035	4	*g-DSF-08-3*	/	/	/	/	/	⑨
Glyma08g43930	Block_8_43701221_43703343	43701221	43703343	3	/	/	*g-AAT*$_{DSF}$*-08-8*	/	/	/	⑩
Glyma08g44170	8_43905780_Block_8_43901065_43906255	43901065	43906255	2	/	/	/	/	/	*g-AAT*$_{DFM}$*-08-7*	⑩
Glyma08g46480	8_45566429_Block_8_45566371_45566590	45566371	45566590	2	/	/	/	/	*g-ADL*$_{DFM}$*-08-5*	/	⑩
Glyma08g47790	Block_8_46591084_46595539	46591084	46595539	3	/	/	/	*g-DFM-08-*	/	/	⑨
Glyma09g02470	Block_9_1660441_1673841	1660441	1673841	3	*g-DSF-09-1*	/	*g-AAT*$_{DSF}$*-09-1*	/	/	/	⑧
Glyma09g03270	Block_9_2299604_2301800	2299604	2301800	3	*g-DSF-09-2*	/	/	/	/	/	⑥
Glyma09g05600	Block_9_4370951_4375513	4370951	4375513	3	/	/	/	/	*g-ADL*$_{DFM}$*-09-1*	/	⑧
Glyma09g05830	Block_9_4575404_4584274	4575404	4584274	3	/	/	/	/	*g-ADL*$_{DFM}$*-09-2*	/	⑥
Glyma09g07661	9_6583848_Block_9_6578141_6584984	6578141	6584984	2	/	/	*g-AAT*$_{DSF}$*-09-2*	/	/	/	③
Glyma09g07760	Block_9_6694656_6699276	6694656	6699276	5	/	/	*g-AAT*$_{DSF}$*-09-3*	/	*g-ADL*$_{DFM}$*-09-3*	/	⑩
Glyma09g07803	Block_9_6807007_6807607	6807007	6807607	3	*g-DSF-09-3*	/	/	/	/	/	⑩
Glyma09g08940	Block_9_8400045_8401115	8400045	8401115	2	*g-DSF-09-4*	/	/	/	/	/	⑩
Glyma09g14100	Block_9_16051707_16054185	16051707	16054185	2	/	/	/	*g-DFM-09-1*	/	/	①/④/⑤
Glyma09g16566	9_19825694_Block_9_19825263_19825844	19825263	19825844	2	/	/	/	*g-DFM-09-2*	/	/	①
Glyma09g21070	Block_9_25932104_25938309	25932104	25938309	3	/	*g-ADL*$_{DSF}$*-09-1*	/	/	/	/	⑥
Glyma09g25215	9_31250163_Block_9_31248431_31268714	31248431	31268714	2	*g-DSF-09-5*	/	*g-AAT*$_{DSF}$*-09-4*	/	/	/	④/⑥
Glyma09g26100	9_32368815_Block_9_32368761_32373907	32368761	32373907	2	/	/	/	/	*g-ADL*$_{DFM}$*-09-4*	/	⑥/⑦
Glyma09g26970	9_33647491_Block_9_33640726_33647666	33640726	33647666	2	/	/	/	/	/	*g-AAT*$_{DFM}$*-09-1*	①
Glyma09g27940	Block_9_34892968_34904685	34892968	34904685	4	/	/	*g-AAT*$_{DSF}$*-09-5*	/	/	/	⑥
Glyma09g28620	Block_9_35533347_35543483	35533347	35543483	4	/	*g-ADL*$_{DSF}$*-09-2*	*g-AAT*$_{DSF}$*-09-6*	/	/	/	⑥
Glyma09g31087	9_37848613_Block_9_37848070_37850595	37848070	37850595	2	*g-DSF-09-6*	*g-ADL*$_{DSF}$*-09-3*	/	/	/	/	⑧
Glyma09g31850	9_38442924_Block_9_38441650_38446135	38441650	38446135	2	/	/	/	/	*g-ADL*$_{DFM}$*-09-5*	/	②
Glyma09g31880	9_38477162_Block_9_38476089_38478338	38476089	38478338	2	/	*g-ADL*$_{DSF}$*-09-4*	/	/	/	/	⑤/⑦
Glyma09g32350	Block_9_38895413_38899033	38895413	38899033	3	/	/	*g-AAT*$_{DSF}$*-09-7*	/	/	/	⑩
Glyma09g33010	9_39538681_Block_9_39534380_39539694	39534380	39539694	2	/	/	/	/	*g-ADL*$_{DFM}$*-09-6*	/	②
Glyma09g33060	Block_9_39589660_39601707	39589660	39601707	3	/	/	/	/	*g-ADL*$_{DFM}$*-09-7*	/	①
Glyma09g33737	9_40208037_Block_9_40204509_40208918	40204509	40208918	2	/	/	/	/	/	*g-AAT*$_{DFM}$*-09-2*	⑧
Glyma09g34750	Block_9_41049391_41052262	41049391	41052262	3	/	*g-ADL*$_{DSF}$*-09-5*	/	/	/	/	⑦
Glyma09g34850	Block_9_41134020_41148470	41134020	41148470	4	*g-DSF-09-7*	/	*g-AAT*$_{DSF}$*-09-8*	/	/	/	⑨
Glyma09g34940	Block_9_41208615_41217803	41208615	41217803	2	*g-DSF-09-8*	/	/	/	/	/	⑧/⑨
Glyma09g35651	9_41610410_Block_9_41608946_41610703	41608946	41610703	2	/	*g-ADL*$_{DSF}$*-09-6*	/	/	/	/	⑩
Glyma09g36030	Block_9_41902550_41905039	41902550	41905039	3	/	*g-ADL*$_{DSF}$*-09-7*	/	/	/	/	⑩
Glyma09g37580	9_43103646_Block_9_43102797_43106909	43102797	43106909	2	/	*g-ADL*$_{DSF}$*-09-8*	/	/	/	/	⑧
Glyma09g37840	Block_9_43365821_43367092	43365821	43367092	3	/	/	/	*g-DFM-09-3*	/	/	⑦/⑧
Glyma09g40181	9_45132400_Block_9_45131725_45136238	45131725	45136238	2	*g-DSF-09-9*	/	/	/	/	/	⑦
Glyma09g40230	Block_9_45183691_45200156	45183691	45200156	4	*g-DSF-09-10*	/	/	/	/	/	③/⑤/⑦
Glyma09g40420	9_45344464_Block_9_45343525_45345356	45343525	45345356	2	/	/	/	*g-DFM-09-4*	/	/	⑩
Glyma09g41010	9_45780183_Block_9_45777856_45781824	45777856	45781824	2	/	/	/	/	*g-ADL*$_{DFM}$*-09-8*	/	⑥/⑦
Glyma09g41740	Block_9_46386025_46392191	46386025	46392191	4	/	/	/	/	/	*g-AAT*$_{DFM}$*-09-3*	⑥
Glyma10g01610	Block_10_1173030_1174816	1173030	1174816	4	/	*g-ADL*$_{DSF}$*-10-1*	/	/	/	/	②
Glyma10g04440	10_3429868_Block_10_3425870_3432231	3425870	3432231	2	/	*g-ADL*$_{DSF}$*-10-2*	/	/	/	/	⑩
Glyma10g05051	Block_10_3928785_3931038	3928785	3931038	5	/	/	/	/	/	*g-AAT*$_{DFM}$*-10-1*	①
Glyma10g05100	10_3962179_Block_10_3958599_3962283	3958599	3962283	2	/	/	/	/	*g-ADL*$_{DFM}$*-10-1*	/	⑩
Glyma10g05240	10_4055634_Block_10_4052536_4058533	4052536	4058533	2	/	/	/	*g-DFM-10-1*	/	/	⑩
Glyma10g05451	10_4226921_Block_10_4226578_4229396	4226578	4229396	2	/	/	/	/	*g-ADL*$_{DFM}$*-10-2*	/	⑩
Glyma10g05600	10_4391852_Block_10_4390605_4397865	4390605	4397865	2	/	/	/	/	/	*g-AAT*$_{DFM}$*-10-2*	⑧
Glyma10g07601	Block_10_6336194_6353470	6336194	6353470	2	/	/	/	/	*g-ADL*$_{DFM}$*-10-3*	/	④/⑤
Glyma10g11480	10_11665125_Block_10_11665108_11670068	11665108	11670068	2	/	/	/	/	*g-ADL*$_{DFM}$*-10-4*	/	⑨
Glyma10g11700	Block_10_12029401_12038336	12029401	12038336	3	*g-DSF-10-1*	/	/	/	/	/	⑩
Glyma10g14916	Block_10_17311225_17312264	17311225	17312264	3	/	*g-ADL*$_{DSF}$*-10-3*	/	/	/	/	①
Glyma10g17510	Block_10_21068632_21074901	21068632	21074901	3	/	/	/	/	*g-ADL*$_{DFM}$*-10-5*	/	⑩
Glyma10g19040	10_23575540_Block_10_23562546_23580815	23562546	23580815	2	*g-DSF-10-2*	/	/	/	/	/	⑩
Glyma10g24391	Block_10_31735985_31788880	31735985	31788880	4	/	/	*g-AAT*$_{DSF}$*-10-1*	/	/	/	⑤
Glyma10g26450	Block_10_34811615_34849713	34811615	34849713	5	*g-DSF-10-3*	*g-ADL*$_{DSF}$*-10-4*	*g-AAT*$_{DSF}$*-10-2*	*g-DFM-10-2*	/	/	③/④/⑤
Glyma10g28380	Block_10_37284406_37285368	37284406	37285368	3	/	/	/	/	/	*g-AAT*$_{DFM}$*-10-3*	⑤
Glyma10g29380	10_38238991_Block_10_38238481_38240608	38238481	38240608	2	*g-DSF-10-4*	/	/	/	/	/	②
Glyma10g29970	Block_10_38676542_38684098	38676542	38684098	4	/	*g-ADL*$_{DSF}$*-10-5*	/	*g-DFM-10-3*	*g-ADL*$_{DFM}$*-10-6*	/	⑩
Glyma10g30100	Block_10_38803651_38835517	38803651	38835517	3	*g-DSF-10-5*	/	/	/	/	/	⑥

Glyma10g30930	Block_10_39522660_39535366	39522660	39535366	3	/	g-ADL$_{DSF}$-10-6	/	/	/	g-AAT$_{DFM}$-10-4	⑩
Glyma10g31570	Block_10_40054950_40064518	40054950	40064518	3	/	g-ADL$_{DSF}$-10-7	/	/	/	g-AAT$_{DFM}$-10-5	⑤
Glyma10g31630	10_40129301_Block_10_40126866_40136942	40126866	40136942	2	/	/	/	g-DFM-10-4	/	/	⑧
Glyma10g32840	10_41219935_Block_10_41219377_41224009	41219377	41224009	2	g-DSF-10-6	/	/	/	/	/	⑤
Glyma10g34490	Block_10_42652341_42660661	42652341	42660661	2	/	/	g-AAT$_{DSF}$-10-3	/	/	/	⑨
Glyma10g35640	Block_10_43900600_43905731	43900600	43905731	3	/	g-ADL$_{DSF}$-10-8	/	/	/	/	⑩
Glyma10g35960	10_44166758_Block_10_44166593_44171098	44166593	44171098	2	g-DSF-10-7	/	g-AAT$_{DSF}$-10-4	/	/	/	⑦
Glyma10g36600	Block_10_44716720_44738268	44716720	44738268	2	/	/	/	/	g-ADL$_{DFM}$-10-7	/	①/②/⑤
Glyma10g38360	10_46152334_Block_10_46151770_46155560	46151770	46155560	2	g-DSF-10-8	/	/	/	/	/	⑨
Glyma10g41540	Block_10_48581481_48592231	48581481	48592231	3	g-DSF-10-9	/	/	/	/	/	⑤
Glyma10g41573	10_48621798_Block_10_48621686_48622700	48621686	48622700	2	/	g-ADL$_{DSF}$-10-9	/	/	/	/	⑥
Glyma10g41710	Block_10_48727688_48732259	48727688	48732259	3	/	g-ADL$_{DSF}$-10-10	/	/	/	/	⑩
Glyma10g41880	10_48872958_Block_10_48872622_48878953	48872622	48878953	2	/	/	/	g-DFM-10-5	/	/	⑧
Glyma10g42161	10_49186092_Block_10_49185759_49186371	49185759	49186371	2	/	/	/	/	g-ADL$_{DFM}$-10-8	/	④
Glyma10g44540	10_50864800_Block_10_50858328_50873849	50858328	50873849	2	/	g-ADL$_{DSF}$-10-11	/	/	/	/	⑧/⑨
Glyma11g00600	Block_11_265093_267332	265093	267332	3	/	g-ADL$_{DSF}$-11-1	/	/	/	/	⑤
Glyma11g03130	11_2066444_Block_11_2065378_2068313	2065378	2068313	2	/	g-ADL$_{DSF}$-11-2	/	/	/	/	⑩
Glyma11g05990	Block_11_4224828_4230097	4224828	4230097	3	/	/	g-AAT$_{DSF}$-11-1	/	/	/	⑩
Glyma11g06565	Block_11_4592508_4615467	4592508	4615467	3	/	/	/	/	/	g-AAT$_{DFM}$-11-1	⑧
Glyma11g07620	Block_11_5330309_5334423	5330309	5334423	4	/	/	/	g-DFM-11-1	/	/	⑦
Glyma11g09060	11_6408681_Block_11_6405862_6409752	6405862	6409752	2	g-DSF-11-1	/	/	/	/	/	⑦/⑧
Glyma11g09940	11_7102434_Block_11_7101664_7105757	7101664	7105757	2	/	/	/	/	/	g-AAT$_{DFM}$-11-2	①/④
Glyma11g10100	Block_11_7212107_7225504	7212107	7225504	3	g-DSF-11-2	/	/	/	/	/	①/⑧
Glyma11g10430	Block_11_7462670_7466540	7462670	7466540	2	/	/	/	/	/	g-AAT$_{DFM}$-11-3	⑩
Glyma11g10490	Block_11_7497883_7501606	7497883	7501606	3	/	/	g-AAT$_{DSF}$-11-2	/	/	/	⑩
Glyma11g10600	Block_11_7541681_7556953	7541681	7556953	4	/	/	/	/	g-ADL$_{DFM}$-11-1	/	⑧
Glyma11g10800	Block_11_7670011_7688634	7670011	7688634	4	/	/	/	g-DFM-11-2	g-ADL$_{DFM}$-11-2	/	⑩
Glyma11g13111	11_9350699_Block_11_9349163_9350743	9349163	9350743	2	/	/	/	g-DFM-11-3	/	g-AAT$_{DFM}$-11-4	⑩
Glyma11g14300	11_10251477_Block_11_10251075_10254154	10251075	10254154	2	/	/	/	g-DFM-11-4	/	/	⑩
Glyma11g14500	Block_11_10343999_10351257	10343999	10351257	3	g-DSF-11-3	/	g-AAT$_{DSF}$-11-3	/	/	g-AAT$_{DFM}$-11-5	⑩
Glyma11g17930	11_14382076_Block_11_14380273_14383275	14380273	14383275	2	/	/	/	/	/	g-AAT$_{DFM}$-11-6	③/⑧
Glyma11g19400	11_16088699_Block_11_16083609_16089141	16083609	16089141	2	g-DSF-11-4	/	/	/	/	/	⑩
Glyma11g19670	Block_11_16445517_16464186	16445517	16464186	3	g-DSF-11-5	/	g-AAT$_{DSF}$-11-4	/	/	/	⑧
Glyma11g19735	11_16520623_Block_11_16520056_16521297	16520056	16521297	2	/	/	/	g-DFM-11-5	/	/	⑩
Glyma11g20520	Block_11_17333852_17340583	17333852	17340583	3	/	/	/	/	/	g-AAT$_{DFM}$-11-7	⑤
Glyma11g25900	11_24638350_Block_11_24631407_24640036	24631407	24640036	2	/	/	g-AAT$_{DSF}$-11-5	/	/	/	③
Glyma11g27510	Block_11_27324458_27333937	27324458	27333937	3	/	g-ADL$_{DSF}$-11-3	/	/	/	/	⑤
Glyma11g37010	Block_11_38265014_38268252	38265014	38268252	4	/	g-ADL$_{DSF}$-11-4	/	/	/	/	⑧
Glyma11g37040	11_38281268_Block_11_38278427_38281487	38278427	38281487	2	/	g-ADL$_{DSF}$-11-5	/	g-DFM-11-6	/	/	⑩
Glyma11g37110	11_38334922_Block_11_38334231_38337768	38334231	38337768	2	g-DSF-11-6	/	/	/	/	/	①
Glyma12g02340	Block_12_1487830_1490922	1487830	1490922	3	/	/	/	/	g-ADL$_{DFM}$-12-1	/	⑤
Glyma12g02800	12_1806889_Block_12_1806279_1809621	1806279	1809621	2	/	/	/	/	/	g-AAT$_{DFM}$-12-1	⑩
Glyma12g03580	Block_12_2387698_2391706	2387698	2391706	3	/	/	/	g-DFM-12-1	/	/	⑥
Glyma12g05780	Block_12_3878517_3882389	3878517	3882389	4	g-DSF-12-1	/	/	/	/	/	⑨
Glyma12g06580	Block_12_4468579_4479376	4468579	4479376	3	/	g-ADL$_{DSF}$-12-1	/	g-DFM-12-2	g-ADL$_{DFM}$-12-2	g-AAT$_{DFM}$-12-2	⑧
Glyma12g06620	12_4503169_Block_12_4501755_4504431	4501755	4504431	2	/	g-ADL$_{DSF}$-12-2	/	g-DFM-12-3	/	g-AAT$_{DFM}$-12-3	⑧
Glyma12g06950	12_4739271_Block_12_4738726_4739974	4738726	4739974	2	/	/	/	g-DFM-12-4	g-ADL$_{DFM}$-12-3	g-AAT$_{DFM}$-12-4	①
Glyma12g08000	Block_12_5663311_5669083	5663311	5669083	4	g-DSF-12-2	/	g-AAT$_{DSF}$-12-1	/	/	/	⑩
Glyma12g08100	Block_12_5821873_5831329	5821873	5831329	5	/	/	/	/	/	g-AAT$_{DFM}$-12-5	⑤/⑧
Glyma12g08170	Block_12_5914480_5919778	5914480	5919778	4	/	/	/	/	/	g-AAT$_{DFM}$-12-6	①/④/⑤
Glyma12g08390	12_6150215_Block_12_6150009_6152455	6150009	6152455	2	/	/	/	/	g-ADL$_{DFM}$-12-4	/	⑨
Glyma12g08860	Block_12_6617948_6624348	6617948	6624348	2	/	/	/	g-DFM-12-5	/	/	①
Glyma12g10880	Block_12_8887455_8891720	8887455	8891720	3	/	/	g-AAT$_{DSF}$-12-2	/	/	/	⑩
Glyma12g10890	Block_12_8899651_8908334	8899651	8908334	3	/	g-ADL$_{DSF}$-12-3	/	/	/	/	⑤
Glyma12g14508	Block_12_13421239_13425372	13421239	13425372	3	/	/	/	g-DFM-12-6	/	/	⑧
Glyma12g14530	Block_12_13461156_13466569	13461156	13466569	2	/	/	/	g-DFM-12-7	/	/	⑥
Glyma12g30870	Block_12_34465987_34478819	34465987	34478819	3	g-DSF-12-3	/	/	/	/	/	⑩
Glyma12g33700	12_36911058_Block_12_36910852_36912867	36910852	36912867	2	/	/	/	g-DFM-12-8	/	/	⑩
Glyma12g33940	12_37129476_Block_12_37128808_37132255	37128808	37132255	2	/	/	/	g-DFM-12-9	/	/	⑩
Glyma12g34830	Block_12_37957118_37958990	37957118	37958990	3	g-DSF-12-4	/	g-AAT$_{DSF}$-12-3	/	/	/	⑩
Glyma12g35580	12_38720904_Block_12_38714312_38722934	38714312	38722934	2	/	/	/	/	g-ADL$_{DFM}$-12-5	/	④
Glyma12g36990	Block_12_39980155_39984908	39980155	39984908	4	g-DSF-12-5	/	/	/	/	/	⑩
Glyma13g00960	Block_13_684885_691843	684885	691843	2	/	/	/	/	/	g-AAT$_{DFM}$-13-1	⑤
Glyma13g02620	Block_13_2575157_2581713	2575157	2581713	3	/	/	g-AAT$_{DSF}$-13-1	/	/	/	④/⑤

Glyma13g06400	Block_13_6646239_6649541	6646239	6649541	*4*	/	/	/	*g-DFM-13-1*	/	/	⑩
Glyma13g07110	Block_13_7287388_7291220	7287388	7291220	*3*	*g-DSF-13-1*	*g-ADL$_{DSF}$-13-1*	*g-AAT$_{DSF}$-13-2*	/	/	/	⑦
Glyma13g08035	Block_13_8281823_8290126	8281823	8290126	*3*	/	/	/	*g-DFM-13-2*	/	/	⑩
Glyma13g08490	13_8984336_Block_13_8983497_8985302	8983497	8985302	*2*	*g-DSF-13-2*	/	/	/	/	/	⑤
Glyma13g09341	13_10483239_Block_13_10473465_10483425	10473465	10483425	*2*	/	*g-ADL$_{DSF}$-13-2*	/	/	/	/	⑧
Glyma13g09380	Block_13_10605520_10610298	10605520	10610298	*7*	/	*g-ADL$_{DSF}$-13-3*	/	/	/	/	⑩
Glyma13g09470	Block_13_10895574_10904406	10895574	10904406	*5*	/	/	/	*g-DFM-13-3*	*g-ADL$_{DFM}$-13-1*	*g-AAT$_{DFM}$-13-2*	⑩
Glyma13g09970	Block_13_11526888_11545834	11526888	11545834	*6*	*g-DSF-13-3*	/	/	*g-DFM-13-4*	/	/	⑩
Glyma13g16940	Block_13_20802267_20807803	20802267	20807803	*3*	/	/	/	*g-DFM-13-5*	/	/	⑦
Glyma13g21340	Block_13_24828124_24832582	24828124	24832582	*3*	*g-DSF-13-4*	/	*g-AAT$_{DSF}$-13-3*	*g-DFM-13-6*	/	/	⑧
Glyma13g22100	13_25685474_Block_13_25681768_25685789	25681768	25685789	*2*	/	/	/	/	/	*g-AAT$_{DFM}$-13-3*	⑥
Glyma13g22420	13_25945441_Block_13_25941052_25946858	25941052	25946858	*2*	/	*g-ADL$_{DSF}$-13-4*	/	/	/	*g-AAT$_{DFM}$-13-4*	①/④/⑤
Glyma13g22855	Block_13_26354979_26359754	26354979	26359754	*3*	/	/	/	/	/	*g-AAT$_{DFM}$-13-5*	①
Glyma13g22870	Block_13_26364606_26370673	26364606	26370673	*3*	/	/	/	*g-DFM-13-7*	/	/	⑩
Glyma13g23700	Block_13_27046462_27049969	27046462	27049969	*4*	/	*g-ADL$_{DSF}$-13-5*	/	/	/	/	①/⑤
Glyma13g23850	Block_13_27156006_27164469	27156006	27164469	*2*	/	/	*g-AAT$_{DSF}$-13-4*	/	/	/	③/⑤/⑧
Glyma13g24820	Block_13_28131494_28133242	28131494	28133242	*4*	/	/	/	/	/	*g-AAT$_{DFM}$-13-6*	⑩
Glyma13g25020	Block_13_28314232_28322630	28314232	28322630	*5*	/	/	/	/	*g-ADL$_{DFM}$-13-2*	/	⑩
Glyma13g25440	Block_13_28671432_28677013	28671432	28677013	*3*	/	/	/	/	*g-ADL$_{DFM}$-13-3*	/	⑦
Glyma13g25480	Block_13_28726715_28731381	28726715	28731381	*6*	*g-DSF-13-5*	/	*g-AAT$_{DSF}$-13-5*	*g-DFM-13-8*	*g-ADL$_{DFM}$-13-4*	*g-AAT$_{DFM}$-13-7*	④
Glyma13g26270	Block_13_29477026_29479751	29477026	29479751	*4*	/	/	/	*g-DFM-13-9*	/	/	⑧
Glyma13g28260	Block_13_31295155_31304666	31295155	31304666	*3*	/	*g-ADL$_{DSF}$-13-6*	/	/	/	*g-AAT$_{DFM}$-13-8*	⑩
Glyma13g28280	Block_13_31329469_31331182	31329469	31331182	*4*	/	/	/	*g-DFM-13-*	/	/	⑧
Glyma13g28570	13_31572160_Block_13_31566310_31573126	31566310	31573126	*2*	/	/	/	*g-DFM-13-*	/	/	⑧
Glyma13g28880	Block_13_31844610_31849725	31844610	31849725	*4*	/	/	/	*g-DFM-13-*	*g-ADL$_{DFM}$-13-5*	/	⑤
Glyma13g29020	13_31994298_Block_13_31993588_31994897	31993588	31994897	*2*	/	/	*g-AAT$_{DSF}$-13-6*	/	/	/	⑩
Glyma13g29160	Block_13_32078442_32088927	32078442	32088927	*4*	/	/	/	/	/	*g-AAT$_{DFM}$-13-9*	⑤
Glyma13g30420	Block_13_33060875_33064957	33060875	33064957	*3*	*g-DSF-13-6*	/	/	/	/	/	⑩
Glyma13g33260	13_35134648_Block_13_35134288_35142160	35134288	35142160	*2*	*g-DSF-13-7*	/	/	/	/	/	⑧
Glyma13g34460	Block_13_36025857_36027836	36025857	36027836	*3*	/	/	/	/	*g-ADL$_{DFM}$-13-6*	/	⑤
Glyma13g36310	13_37593284_Block_13_37588242_37593338	37588242	37593338	*2*	/	/	/	/	*g-ADL$_{DFM}$-13-7*	/	④
Glyma13g37360	Block_13_38473481_38475796	38473481	38475796	*3*	/	*g-ADL$_{DSF}$-13-7*	/	/	/	/	⑩
Glyma13g37980	Block_13_38910618_38916143	38910618	38916143	*5*	/	*g-ADL$_{DSF}$-13-8*	/	/	/	/	⑧
Glyma13g38860	13_39578208_Block_13_39578094_39583512	39578094	39583512	*2*	/	/	/	*g-DFM-13-*	/	/	⑧
Glyma13g41650	Block_13_41885202_41887421	41885202	41887421	*3*	/	/	*g-AAT$_{DSF}$-13-7*	/	/	/	⑥
Glyma13g41660	Block_13_41891593_41896067	41891593	41896067	*3*	/	*g-ADL$_{DSF}$-13-9*	/	/	/	/	②
Glyma13g43050	13_42824070_Block_13_42821728_42825940	42821728	42825940	*2*	*g-DSF-13-8*	/	/	/	/	/	⑤/⑦
Glyma13g43320	Block_13_43059510_43062868	43059510	43062868	*4*	/	/	*g-AAT$_{DSF}$-13-8*	/	/	/	①/④
Glyma13g43710	13_43321675_Block_13_43320620_43324933	43320620	43324933	*2*	/	/	/	*g-DFM-13-*	/	/	⑩
Glyma13g43740	Block_13_43347258_43352207	43347258	43352207	*3*	/	/	/	*g-DFM-13-*	/	/	④/⑤
Glyma13g43980	Block_13_43555734_43558530	43555734	43558530	*3*	/	/	/	/	/	*g-AAT$_{DFM}$-13-10*	⑩
Glyma14g00240	Block_14_38566_55214	38566	55214	*4*	/	/	*g-AAT$_{DSF}$-14-1*	/	/	/	②
Glyma14g01300	14_731787_Block_14_729911_732076	729911	732076	*2*	/	/	/	/	/	*g-AAT$_{DFM}$-14-1*	②
Glyma14g02780	Block_14_1754725_1777694	1754725	1777694	*3*	/	*g-ADL$_{DSF}$-14-1*	/	/	/	*g-AAT$_{DFM}$-14-2*	⑤
Glyma14g02790	Block_14_1779811_1782749	1779811	1782749	*4*	/	/	/	*g-DFM-14-1*	/	/	⑥
Glyma14g02970	Block_14_1874611_1878417	1874611	1878417	*3*	/	*g-ADL$_{DSF}$-14-2*	/	/	/	/	③
Glyma14g04550	14_3127412_Block_14_3125618_3129187	3125618	3129187	*2*	*g-DSF-14-1*	/	/	/	/	/	②/⑤/⑥/⑦
Glyma14g04690	14_3229763_Block_14_3228212_3230566	3228212	3230566	*2*	/	/	*g-AAT$_{DSF}$-14-2*	/	/	/	⑥
Glyma14g04930	Block_14_3397622_3417976	3397622	3417976	*3*	/	/	/	/	/	*g-AAT$_{DFM}$-14-3*	⑤
Glyma14g05140	14_3557369_Block_14_3556870_3559677	3556870	3559677	*2*	/	/	*g-AAT$_{DSF}$-14-3*	/	/	*g-AAT$_{DFM}$-14-4*	⑩
Glyma14g05780	Block_14_4175805_4176959	4175805	4176959	*3*	/	/	/	*g-DFM-14-2*	/	/	⑩
Glyma14g06485	Block_14_4732326_4736259	4732326	4736259	*2*	/	/	/	/	/	*g-AAT$_{DFM}$-14-5*	②
Glyma14g07960	14_6020513_Block_14_6019214_6022585	6019214	6022585	*2*	/	/	/	*g-DFM-14-3*	/	/	⑧
Glyma14g08220	Block_14_6246265_6255276	6246265	6255276	*3*	*g-DSF-14-2*	/	/	/	/	/	⑦
Glyma14g08990	14_6980197_Block_14_6977153_6982492	6977153	6982492	*2*	/	/	/	/	/	*g-AAT$_{DFM}$-14-6*	⑩
Glyma14g09160	Block_14_7156524_7165117	7156524	7165117	*3*	/	/	/	/	/	*g-AAT$_{DFM}$-14-7*	⑤
Glyma14g09720	14_7784672_Block_14_7783126_7789941	7783126	7789941	*2*	/	/	*g-AAT$_{DSF}$-14-4*	/	/	/	⑩
Glyma14g10790	14_8993214_Block_14_8983532_8993833	8983532	8993833	*2*	/	/	/	*g-DFM-14-4*	/	/	⑧
Glyma14g11910	14_10466685_Block_14_10466668_10467124	10466668	10467124	*2*	*g-DSF-14-3*	/	/	/	/	/	⑩
Glyma14g13178	Block_14_12402128_12404459	12402128	12404459	*3*	/	/	/	/	/	*g-AAT$_{DFM}$-14-8*	①
Glyma14g13884	Block_14_13496054_13501056	13496054	13501056	*3*	/	/	/	/	*g-ADL$_{DFM}$-14-1*	/	⑧
Glyma14g14000	Block_14_13739910_13746899	13739910	13746899	*3*	/	*g-ADL$_{DSF}$-14-3*	/	/	/	/	⑤/⑧
Glyma14g16655	14_18101116_Block_14_18100820_18101237	18100820	18101237	*2*	/	/	/	*g-DFM-14-5*	/	/	⑩
Glyma14g24140	Block_14_28802503_28809364	28802503	28809364	*3*	*g-DSF-14-4*	/	/	/	/	/	⑦

Glyma14g24480	Block_14_29369049_29373797	29369049	29373797	5	/	*g-ADL*$_{DSF}$*-14-4*	/	/	/	/	①/④/⑤
Glyma14g33601	Block_14_41615159_41617103	41615159	41617103	2	/	*g-ADL*$_{DSF}$*-14-5*	/	/	/	/	⑥
Glyma14g34340	Block_14_42804094_42808115	42804094	42808115	3	*g-DSF-14-5*	/	/	/	/	/	⑩
Glyma14g36980	14_46293036_Block_14_46290924_46296420	46290924	46296420	2	/	/	*g-AAT*$_{DSF}$*-14-5*	/	/	/	⑥
Glyma14g37100	Block_14_46401128_46404683	46401128	46404683	2	*g-DSF-14-6*	/	/	/	/	/	⑩
Glyma14g37230	14_46505411_Block_14_46504560_46509968	46504560	46509968	2	/	/	/	/	/	*g-AAT*$_{DFM}$*-14-9*	②
Glyma14g37330	Block_14_46632830_46636073	46632830	46636073	3	*g-DSF-14-7*	*g-ADL*$_{DSF}$*-14-6*	/	/	/	/	⑤/⑥
Glyma14g37400	Block_14_46677514_46682923	46677514	46682923	5	*g-DSF-14-8*	/	/	/	/	/	②
Glyma14g38570	Block_14_47699935_47706202	47699935	47706202	5	*g-DSF-14-9*	/	/	/	/	*g-AAT*$_{DFM}$*-14-10*	⑩
Glyma14g38670	Block_14_47831439_47847037	47831439	47847037	3	/	/	/	*g-DFM-14-6*	/	/	⑧
Glyma14g38910	Block_14_48074448_48076103	48074448	48076103	4	/	*g-ADL*$_{DSF}$*-14-7*	/	/	/	/	⑩
Glyma14g39230	14_48338138_Block_14_48337568_48342410	48337568	48342410	2	/	/	/	/	*g-ADL*$_{DFM}$*-14-2*	/	⑨
Glyma14g39375	Block_14_48487254_48491153	48487254	48491153	3	/	/	*g-AAT*$_{DSF}$*-14-6*	/	/	/	⑩
Glyma15g00460	15_225361_Block_15_223000_226044	223000	226044	2	/	/	*g-AAT*$_{DSF}$*-15-1*	/	/	/	⑧
Glyma15g02740	Block_15_1889553_1895693	1889553	1895693	3	/	/	/	/	/	*g-AAT*$_{DFM}$*-15-1*	⑤
Glyma15g03770	15_2637919_Block_15_2636612_2643403	2636612	2643403	2	*g-DSF-15-1*	/	/	/	/	/	⑩
Glyma15g04891	15_3468251_Block_15_3463074_3468299	3463074	3468299	2	/	/	*g-AAT*$_{DSF}$*-15-2*	/	/	/	⑩
Glyma15g08420	15_5968645_Block_15_5967239_5969370	5967239	5969370	2	*g-DSF-15-2*	/	*g-AAT*$_{DSF}$*-15-3*	/	*g-ADL*$_{DFM}$*-15-1*	/	③/⑧
Glyma15g09930	15_7174832_Block_15_7173630_7176788	7173630	7176788	2	/	/	/	/	/	*g-AAT*$_{DFM}$*-15-2*	⑩
Glyma15g12930	15_9626056_Block_15_9622982_9626519	9622982	9626519	2	/	/	*g-AAT*$_{DSF}$*-15-4*	/	/	/	①/⑤
Glyma15g13410	15_10030713_Block_15_10027724_10032923	10027724	10032923	2	/	/	*g-AAT*$_{DSF}$*-15-5*	/	/	/	⑩
Glyma15g14040	15_10603758_Block_15_10602778_10604820	10602778	10604820	2	/	/	/	/	/	*g-AAT*$_{DFM}$*-15-3*	⑩
Glyma15g15105	Block_15_11548096_11552739	11548096	11552739	3	/	/	/	/	/	*g-AAT*$_{DFM}$*-15-4*	⑦
Glyma15g16000	15_12337588_Block_15_12337020_12338452	12337020	12338452	2	/	*g-ADL*$_{DSF}$*-15-1*	/	/	/	/	⑩
Glyma15g16183	15_12505828_Block_15_12503995_12508047	12503995	12508047	2	/	/	/	/	/	*g-AAT*$_{DFM}$*-15-5*	⑩
Glyma15g17710	15_14114045_Block_15_14112549_14117855	14112549	14117855	2	/	/	/	*g-DFM-15-1*	/	/	⑧
Glyma15g17910	15_14405044_Block_15_14401251_14407375	14401251	14407375	2	*g-DSF-15-3*	/	/	/	/	/	⑩
Glyma15g18280	Block_15_14974894_14976281	14974894	14976281	3	*g-DSF-15-4*	/	/	/	/	*g-AAT*$_{DFM}$*-15-6*	⑩
Glyma15g18840	15_15753798_Block_15_15746068_15759417	15746068	15759417	2	/	/	*g-AAT*$_{DSF}$*-15-6*	/	/	/	⑩
Glyma15g20200	15_18001506_Block_15_18000477_18002377	18000477	18002377	2	/	*g-ADL*$_{DSF}$*-15-2*	/	/	/	/	①
Glyma15g20935	15_19013138_Block_15_19011461_19013314	19011461	19013314	2	/	*g-ADL*$_{DSF}$*-15-3*	/	/	/	/	①/④/⑤
Glyma15g21101	Block_15_19312624_19320437	19312624	19320437	5	/	/	/	*g-DFM-15-2*	/	/	⑦
Glyma15g21400	15_19627890_Block_15_19627448_19628298	19627448	19628298	2	/	/	/	*g-DFM-15-3*	/	/	⑩
Glyma15g25690	Block_15_27227614_27231975	27227614	27231975	6	/	*g-ADL*$_{DSF}$*-15-4*	/	/	/	/	②/⑦
Glyma15g34653	Block_15_39178579_39182949	39178579	39182949	4	/	/	/	/	*g-ADL*$_{DFM}$*-15-2*	/	⑥
Glyma15g34840	Block_15_39414035_39430956	39414035	39430956	3	*g-DSF-15-5*	/	*g-AAT*$_{DSF}$*-15-7*	/	/	/	②/⑧
Glyma15g35941	Block_15_40858558_40869376	40858558	40869376	5	/	/	*g-AAT*$_{DSF}$*-15-8*	/	/	/	⑩
Glyma15g38060	Block_15_44140868_44144263	44140868	44144263	3	/	*g-ADL*$_{DSF}$*-15-5*	/	/	/	/	③/⑦
Glyma15g40635	Block_15_47614494_47615232	47614494	47615232	3	/	*g-ADL*$_{DSF}$*-15-6*	/	/	/	/	⑩
Glyma15g40740	Block_15_47715638_47720661	47715638	47720661	4	/	/	/	*g-DFM-15-4*	/	/	⑧
Glyma15g40860	Block_15_47868869_47873242	47868869	47873242	2	*g-DSF-15-6*	/	/	/	/	/	③
Glyma15g42762	Block_15_50194791_50195479	50194791	50195479	4	/	/	*g-AAT*$_{DSF}$*-15-9*	*g-DFM-15-5*	/	/	⑩
Glyma15g43010	Block_15_50437028_50445529	50437028	50445529	2	/	/	*g-AAT*$_{DSF}$*-15-10*	/	/	/	⑩
Glyma15g43289	Block_15_50799235_50850850	50799235	50850850	3	/	*g-ADL*$_{DSF}$*-15-7*	/	/	/	/	⑧
Glyma16g00480	16_171389_Block_16_169598_172510	169598	172510	2	/	/	*g-AAT*$_{DSF}$*-16-1*	/	/	/	⑤
Glyma16g01570	16_1144494_Block_16_1143927_1144717	1143927	1144717	2	/	/	/	*g-DFM-16-1*	/	/	⑩
Glyma16g01590	Block_16_1161767_1173675	1161767	1173675	3	*g-DSF-16-1*	/	/	/	/	/	⑥/⑧
Glyma16g01640	16_1210399_Block_16_1208340_1211609	1208340	1211609	2	/	/	/	*g-DFM-16-2*	/	/	①
Glyma16g03050	Block_16_2639945_2645061	2639945	2645061	3	/	/	/	/	*g-ADL*$_{DFM}$*-16-1*	/	②/⑦
Glyma16g03320	16_2833761_Block_16_2829076_2834759	2829076	2834759	2	*g-DSF-16-2*	*g-ADL*$_{DSF}$*-16-1*	*g-AAT*$_{DSF}$*-16-2*	/	/	/	①/③/⑧
Glyma16g03860	16_3220100_Block_16_3217456_3225112	3217456	3225112	2	/	/	*g-AAT*$_{DSF}$*-16-3*	/	/	/	⑩
Glyma16g06061	Block_16_5494992_5499137	5494992	5499137	2	*g-DSF-16-3*	/	/	/	/	/	⑩
Glyma16g07081	16_6380074_Block_16_6379430_6380140	6379430	6380140	2	/	/	/	/	/	*g-AAT*$_{DFM}$*-16-1*	⑩
Glyma16g07920	Block_16_7170521_7173384	7170521	7173384	5	/	/	/	/	*g-ADL*$_{DFM}$*-16-2*	/	④⑧
Glyma16g08430	Block_16_7802131_7816483	7802131	7816483	4	/	/	*g-AAT*$_{DSF}$*-16-4*	/	/	/	⑤
Glyma16g21340	16_24112677_Block_16_24104853_24115455	24104853	24115455	2	/	/	/	/	*g-ADL*$_{DFM}$*-16-3*	/	①
Glyma16g25185	Block_16_29113287_29120351	29113287	29120351	5	/	*g-ADL*$_{DSF}$*-16-2*	/	/	/	/	⑩
Glyma16g25500	16_29515255_Block_16_29512183_29516034	29512183	29516034	2	/	/	/	*g-DFM-16-3*	/	*g-AAT*$_{DFM}$*-16-2*	⑩
Glyma16g26070	Block_16_30236691_30240706	30236691	30240706	3	/	/	/	/	*g-ADL*$_{DFM}$*-16-4*	/	⑧
Glyma16g26260	16_30397806_Block_16_30395343_30398007	30395343	30398007	2	*g-DSF-16-4*	/	/	/	/	/	⑤
Glyma16g27521	Block_16_31521384_31526237	31521384	31526237	5	/	/	/	/	*g-ADL*$_{DFM}$*-16-5*	/	⑥
Glyma16g28010	Block_16_31958024_31960129	31958024	31960129	6	/	/	/	*g-DFM-16-4*	/	/	⑩
Glyma16g28900	Block_16_32848703_32854454	32848703	32854454	6	/	/	/	/	/	*g-AAT*$_{DFM}$*-16-3*	⑥
Glyma16g28910	Block_16_32858500_32864802	32858500	32864802	4	*g-DSF-16-5*	/	/	/	/	/	⑥

Glyma16g28950	16_32886079_Block_16_32884194_32887373	32884194	32887373	2	/	/	*g-AAT*$_{DSF}$*-16-5*	/	/	/	⑩
Glyma16g29380	Block_16_33209634_33211251	33209634	33211251	5	/	/	/	/	*g-ADL*$_{DFM}$*-16-6*	/	⑩
Glyma16g31862	Block_16_35127702_35136429	35127702	35136429	6	/	/	/	/	*g-ADL*$_{DFM}$*-16-7*	/	⑤
Glyma16g32650	Block_16_35816783_35821972	35816783	35821972	3	/	/	*g-AAT*$_{DSF}$*-16-6*	/	/	/	①
Glyma16g32900	16_36008357_Block_16_36004646_36008731	36004646	36008731	2	/	/	/	/	/	*g-AAT*$_{DFM}$*-16-4*	⑧
Glyma16g33100	Block_16_36147325_36152970	36147325	36152970	4	/	*g-ADL*$_{DSF}$*-16-3*	/	/	*g-ADL*$_{DFM}$*-16-8*	*g-AAT*$_{DFM}$*-16-5*	⑧
Glyma16g33320	Block_16_36274053_36275350	36274053	36275350	4	/	/	/	*g-DFM-16-5*	/	/	①
Glyma16g33480	Block_16_36382669_36389982	36382669	36389982	3	/	*g-ADL*$_{DSF}$*-16-4*	/	/	/	/	⑩
Glyma16g33490	16_36402209_Block_16_36393979_36402415	36393979	36402415	2	/	/	/	/	/	*g-AAT*$_{DFM}$*-16-6*	⑥
Glyma16g33881	16_36637389_Block_16_36635980_36642060	36635980	36642060	2	*g-DSF-16-6*	/	/	/	/	/	⑧
Glyma16g34500	16_37145466_Block_16_37141578_37146092	37141578	37146092	2	/	/	*g-AAT*$_{DSF}$*-16-7*	/	/	/	⑥
Glyma17g01160	Block_17_665467_668559	665467	668559	3	/	/	/	/	*g-ADL*$_{DFM}$*-17-1*	*g-AAT*$_{DFM}$*-17-1*	⑥
Glyma17g01430	17_882135_Block_17_881726_889368	881726	889368	2	/	*g-ADL*$_{DSF}$*-17-1*	/	/	/	/	⑤
Glyma17g02580	17_1671484_Block_17_1670664_1675695	1670664	1675695	2	*g-DSF-17-1*	/	/	/	/	/	①/⑧
Glyma17g02610	17_1695837_Block_17_1695425_1698332	1695425	1698332	2	/	/	/	/	*g-ADL*$_{DFM}$*-17-2*	/	⑩
Glyma17g03020	Block_17_2013037_2021174	2013037	2021174	3	*g-DSF-17-2*	/	/	/	/	/	①/⑧
Glyma17g03310	17_2199947_Block_17_2199774_2207217	2199774	2207217	2	/	/	/	/	/	*g-AAT*$_{DFM}$*-17-2*	⑤
Glyma17g03700	Block_17_2460791_2467923	2460791	2467923	3	*g-DSF-17-3*	/	/	/	/	*g-AAT*$_{DFM}$*-17-3*	①/⑦/⑧
Glyma17g04580	Block_17_3036494_3043507	3036494	3043507	3	/	*g-ADL*$_{DSF}$*-17-2*	/	/	*g-ADL*$_{DFM}$*-17-3*	/	⑩
Glyma17g04661	17_3116147_Block_17_3107379_3117305	3107379	3117305	2	/	/	/	*g-DFM-17-1*	/	/	⑩
Glyma17g05595	Block_17_3919983_3925841	3919983	3925841	3	/	/	*g-AAT*$_{DSF}$*-17-1*	/	/	/	⑩
Glyma17g07475	17_5476527_Block_17_5475423_5477748	5475423	5477748	2	/	/	*g-AAT*$_{DSF}$*-17-2*	/	/	/	⑥
Glyma17g07520	17_5533847_Block_17_5530231_5535051	5530231	5535051	2	/	/	/	/	*g-ADL*$_{DFM}$*-17-4*	/	⑩
Glyma17g08460	17_6262188_Block_17_6261605_6265100	6261605	6265100	2	*g-DSF-17-4*	*g-ADL*$_{DSF}$*-17-3*	*g-AAT*$_{DSF}$*-17-3*	/	/	/	⑩
Glyma17g08761	Block_17_6450810_6453048	6450810	6453048	3	*g-DSF-17-5*	/	/	/	/	/	①/⑤
Glyma17g09500	Block_17_7036098_7037342	7036098	7037342	3	*g-DSF-17-6*	*g-ADL*$_{DSF}$*-17-4*	*g-AAT*$_{DSF}$*-17-4*	/	/	/	⑩
Glyma17g13030	17_9933234_Block_17_9932589_9937345	9932589	9937345	2	*g-DSF-17-7*	/	/	/	/	/	⑩
Glyma17g13151	17_10076088_Block_17_10072523_10076834	10072523	10076834	2	*g-DSF-17-8*	/	/	/	/	/	⑧
Glyma17g13260	17_10133112_Block_17_10133099_10138244	10133099	10138244	2	*g-DSF-17-9*	/	/	/	/	*g-AAT*$_{DFM}$*-17-4*	⑤
Glyma17g14370	17_11127728_Block_17_11125908_11127930	11125908	11127930	2	/	/	/	*g-DFM-17-2*	/	/	⑤
Glyma17g14700	17_11420924_Block_17_11420112_11426915	11420112	11426915	2	/	*g-ADL*$_{DSF}$*-17-5*	/	/	/	/	⑤
Glyma17g15350	17_12054147_Block_17_12051979_12058670	12051979	12058670	2	/	*g-ADL*$_{DSF}$*-17-6*	/	/	/	/	⑩
Glyma17g18472	17_15859300_Block_17_15858189_15860141	15858189	15860141	2	*g-DSF-17-10*	/	/	/	/	/	⑩
Glyma17g19084	Block_17_16775473_16789795	16775473	16789795	4	/	/	/	/	*g-ADL*$_{DFM}$*-17-5*	*g-AAT*$_{DFM}$*-17-5*	②
Glyma17g19660	Block_17_17984224_17998298	17984224	17998298	3	/	/	/	*g-DFM-17-3*	/	/	⑧
Glyma17g20091	Block_17_18616129_18625258	18616129	18625258	2	/	/	/	/	*g-ADL*$_{DFM}$*-17-6*	/	⑩
Glyma17g20310	Block_17_19036301_19056856	19036301	19056856	3	/	/	/	*g-DFM-17-4*	/	/	⑩
Glyma17g33290	17_36989961_Block_17_36984683_36991283	36984683	36991283	2	/	/	/	/	/	*g-AAT*$_{DFM}$*-17-6*	⑩
Glyma17g34321	Block_17_38197208_38207675	38197208	38207675	4	/	/	/	*g-DFM-17-5*	/	/	⑧
Glyma17g34420	17_38383987_Block_17_38383284_38385387	38383284	38385387	2	/	/	/	/	*g-ADL*$_{DFM}$*-17-7*	/	⑩
Glyma17g35000	Block_17_38955181_38964990	38955181	38964990	3	/	/	/	/	/	*g-AAT*$_{DFM}$*-17-7*	⑥
Glyma17g35130	17_39128805_Block_17_39128793_39130600	39128793	39130600	2	/	*g-ADL*$_{DSF}$*-17-7*	/	/	/	/	⑩
Glyma18g01200	Block_18_614911_619917	614911	619917	2	/	/	*g-AAT*$_{DSF}$*-18-1*	/	/	/	⑩
Glyma18g01330	18_692942_Block_18_692176_695176	692176	695176	2	/	/	/	*g-DFM-18-1*	/	/	③/⑨
Glyma18g03180	Block_18_2092774_2099488	2092774	2099488	3	/	/	*g-AAT*$_{DSF}$*-18-2*	/	/	/	⑩
Glyma18g04820	Block_18_3568173_3572993	3568173	3572993	3	/	*g-ADL*$_{DSF}$*-18-1*	/	/	/	/	①
Glyma18g05730	Block_18_4386790_4391397	4386790	4391397	2	/	/	/	*g-DFM-18-2*	/	/	⑧
Glyma18g06250	18_4826439_Block_18_4825078_4827763	4825078	4827763	2	/	/	/	*g-DFM-18-3*	/	/	⑩
Glyma18g07900	Block_18_6642314_6646565	6642314	6646565	4	*g-DSF-18-1*	/	/	/	/	/	⑥
Glyma18g08180	Block_18_6886734_6894553	6886734	6894553	3	/	*g-ADL*$_{DSF}$*-18-2*	/	/	/	/	⑩
Glyma18g08410	Block_18_7172244_7175673	7172244	7175673	5	*g-DSF-18-2*	/	*g-AAT*$_{DSF}$*-18-3*	/	/	/	⑥
Glyma18g08440	Block_18_7189698_7194501	7189698	7194501	3	/	/	*g-AAT*$_{DSF}$*-18-4*	/	/	/	⑧
Glyma18g10975	Block_18_9819657_9820874	9819657	9820874	5	*g-DSF-18-3*	/	/	/	/	/	⑩
Glyma18g13175	18_12638179_Block_18_12637749_12639473	12637749	12639473	2	/	/	/	*g-DFM-18-4*	/	/	⑩
Glyma18g16761	Block_18_17397537_17415871	17397537	17415871	4	/	*g-ADL*$_{DSF}$*-18-3*	/	/	/	/	⑧
Glyma18g16780	18_17457536_Block_18_17457285_17459790	17457285	17459790	2	/	/	/	*g-DFM-18-5*	/	/	⑥
Glyma18g17395	Block_18_18652381_18659467	18652381	18659467	4	*g-DSF-18-4*	/	*g-AAT*$_{DSF}$*-18-5*	/	/	/	⑧
Glyma18g17515	Block_18_18777277_18788681	18777277	18788681	3	*g-DSF-18-5*	/	*g-AAT*$_{DSF}$*-18-6*	/	/	/	⑩
Glyma18g18220	Block_18_19601073_19605391	19601073	19605391	3	/	/	/	*g-DFM-18-6*	/	/	⑩
Glyma18g18230	Block_18_19605936_19613492	19605936	19613492	3	/	/	/	/	*g-ADL*$_{DFM}$*-18-1*	/	⑩
Glyma18g18931	18_20445481_Block_18_20444942_20447244	20444942	20447244	2	/	/	*g-AAT*$_{DSF}$*-18-7*	/	/	/	⑨
Glyma18g20146	Block_18_22012942_22022208	22012942	22022208	5	/	/	/	*g-DFM-18-7*	/	/	⑩
Glyma18g20820	Block_18_23290240_23305385	23290240	23305385	3	/	/	*g-AAT*$_{DSF}$*-18-8*	/	/	/	⑥
Glyma18g26120	18_30001588_Block_18_30001494_30003858	30001494	30003858	2	/	*g-ADL*$_{DSF}$*-18-4*	*g-AAT*$_{DSF}$*-18-9*	/	/	/	⑧

Glyma18g32696	18_38131474_Block_18_38129417_38133046	38129417	38133046	2	/	/	/	/	/	*g-AAT*$_{DFM}$*-18-1*	⑤
Glyma18g38570	Block_18_46204949_46211919	46204949	46211919	3	/	*g-ADL*$_{DSF}$*-18-5*	/	/	/	/	⑧
Glyma18g42375	Block_18_51370172_51397245	51370172	51397245	5	/	/	/	/	*g-ADL*$_{DFM}$*-18-2*	/	⑤
Glyma18g44761	Block_18_54485987_54490833	54485987	54490833	4	/	/	/	/	*g-ADL*$_{DFM}$*-18-3*	/	①/②/⑧
Glyma18g45930	18_55633624_Block_18_55633146_55633688	55633146	55633688	2	/	/	/	/	*g-ADL*$_{DFM}$*-18-4*	/	⑦
Glyma18g46220	Block_18_55972986_55978204	55972986	55978204	4	*g-DSF-18-6*	/	/	/	/	/	③/⑤
Glyma18g47720	18_57297218_Block_18_57296483_57300364	57296483	57300364	2	/	*g-ADL*$_{DSF}$*-18-6*	/	/	/	/	⑧
Glyma18g48980	Block_18_58376606_58381414	58376606	58381414	3	/	/	*g-AAT*$_{DSF}$*-18-10*	/	/	/	⑧
Glyma18g50725	Block_18_59759202_59760572	59759202	59760572	3	*g-DSF-18-7*	/	/	/	/	/	⑩
Glyma18g50910	Block_18_59918843_59927288	59918843	59927288	5	/	/	*g-AAT*$_{DSF}$*-18-11*	/	/	/	①/⑤
Glyma18g51230	Block_18_60165720_60171309	60165720	60171309	3	/	/	/	/	/	*g-AAT*$_{DFM}$*-18-2*	⑥
Glyma18g51380	18_60294442_Block_18_60291630_60294654	60291630	60294654	2	/	/	/	/	*g-ADL*$_{DFM}$*-18-5*	/	⑥
Glyma18g51970	Block_18_60708698_60714044	60708698	60714044	5	/	/	/	/	/	*g-AAT*$_{DFM}$*-18-3*	⑨
Glyma18g52250	Block_18_60909376_60911025	60909376	60911025	6	/	*g-ADL*$_{DSF}$*-18-7*	/	/	/	/	⑩
Glyma18g52381	Block_18_61002939_61004309	61002939	61004309	4	/	/	*g-AAT*$_{DSF}$*-18-12*	/	/	/	⑩
Glyma18g53545	Block_18_61809358_61858898	61809358	61858898	3	/	/	/	/	*g-ADL*$_{DFM}$*-18-6*	/	①/⑤
Glyma18g53770	Block_18_62047725_62052506	62047725	62052506	3	/	/	/	/	/	*g-AAT*$_{DFM}$*-18-4*	⑩
Glyma18g53823	Block_18_62079452_62111100	62079452	62111100	5	/	/	/	*g-DFM-18-8*	/	/	④/⑥
Glyma19g01536	Block_19_1154863_1156699	1154863	1156699	2	/	/	/	*g-DFM-19-1*	/	/	⑩
Glyma19g03440	Block_19_3454616_3467505	3454616	3467505	4	/	*g-ADL*$_{DSF}$*-19-1*	/	/	/	/	③/⑧
Glyma19g03563	Block_19_3570374_3577489	3570374	3577489	3	/	/	/	*g-DFM-19-2*	/	/	①
Glyma19g03590	Block_19_3628118_3637375	3628118	3637375	4	/	*g-ADL*$_{DSF}$*-19-2*	/	/	*g-ADL*$_{DFM}$*-19-1*	/	⑤
Glyma19g09700	19_11441616_Block_19_11439881_11441814	11439881	11441814	2	/	/	/	/	/	*g-AAT*$_{DFM}$*-19-1*	⑩
Glyma19g18581	Block_19_22679426_22681115	22679426	22681115	3	/	/	/	/	/	*g-AAT*$_{DFM}$*-19-2*	⑦/⑧
Glyma19g23640	Block_19_29052814_29053161	29052814	29053161	4	/	*g-ADL*$_{DSF}$*-19-3*	*g-AAT*$_{DSF}$*-19-1*	/	/	/	②/⑤
Glyma19g23740	19_29216496_Block_19_29215166_29216998	29215166	29216998	2	/	/	/	*g-DFM-19-3*	*g-ADL*$_{DFM}$*-19-2*	/	⑩
Glyma19g25360	19_31573885_Block_19_31571762_31579663	31571762	31579663	2	/	/	/	/	*g-ADL*$_{DFM}$*-19-3*	/	①/⑤/⑨
Glyma19g25980	Block_19_32318849_32323452	32318849	32323452	3	/	/	/	*g-DFM-19-4*	/	/	⑩
Glyma19g26950	Block_19_34125594_34127499	34125594	34127499	4	/	/	/	*g-DFM-19-5*	/	/	⑤/⑧
Glyma19g30690	Block_19_38342154_38346150	38342154	38346150	3	/	/	/	*g-DFM-19-6*	*g-ADL*$_{DFM}$*-19-4*	/	⑩
Glyma19g30750	19_38383377_Block_19_38380326_38388648	38380326	38388648	2	/	/	/	/	/	*g-AAT*$_{DFM}$*-19-3*	⑩
Glyma19g32540	Block_19_40279022_40285753	40279022	40285753	3	/	/	/	/	/	*g-AAT*$_{DFM}$*-19-4*	⑩
Glyma19g32600	Block_19_40353381_40355435	40353381	40355435	3	*g-DSF-19-1*	/	/	/	/	/	①/②/⑤
Glyma19g33000	Block_19_40675292_40679560	40675292	40679560	3	*g-DSF-19-2*	/	/	/	/	*g-AAT*$_{DFM}$*-19-5*	⑧
Glyma19g33210	Block_19_40823339_40832377	40823339	40832377	3	/	/	/	*g-DFM-19-7*	*g-ADL*$_{DFM}$*-19-5*	/	⑥
Glyma19g33760	Block_19_41347340_41350200	41347340	41350200	3	/	/	/	*g-DFM-19-8*	/	/	⑩
Glyma19g34740	19_42346078_Block_19_42344178_42346426	42344178	42346426	2	/	/	/	*g-DFM-19-9*	*g-ADL*$_{DFM}$*-19-6*	*g-AAT*$_{DFM}$*-19-6*	⑤/⑧
Glyma19g35130	19_42689150_Block_19_42688105_42690289	42688105	42690289	2	*g-DSF-19-3*	/	/	/	/	/	⑥
Glyma19g36710	19_43972602_Block_19_43971782_43978119	43971782	43978119	2	/	/	*g-AAT*$_{DSF}$*-19-2*	/	/	/	②/⑤
Glyma19g37230	Block_19_44449368_44454119	44449368	44454119	4	/	*g-ADL*$_{DSF}$*-19-4*	/	/	/	/	⑩
Glyma19g37840	19_44939715_Block_19_44938084_44939959	44938084	44939959	2	/	/	*g-AAT*$_{DSF}$*-19-3*	/	/	/	⑩
Glyma19g38331	Block_19_45251572_45253628	45251572	45253628	2	/	/	*g-AAT*$_{DSF}$*-19-4*	/	/	/	⑧
Glyma19g39460	19_46127170_Block_19_46122809_46127574	46122809	46127574	2	/	*g-ADL*$_{DSF}$*-19-5*	/	/	/	/	⑤
Glyma19g40710	19_47046223_Block_19_47045778_47047319	47045778	47047319	2	/	/	*g-AAT*$_{DSF}$*-19-5*	/	*g-ADL*$_{DFM}$*-19-7*	/	⑩
Glyma19g42340	19_48358156_Block_19_48357660_48363317	48357660	48363317	2	/	/	*g-AAT*$_{DSF}$*-19-6*	/	/	/	①/⑧
Glyma19g42710	19_48606661_Block_19_48604283_48607757	48604283	48607757	2	*g-DSF-19-4*	/	/	/	/	/	⑥
Glyma19g43320	19_49010497_Block_19_49003312_49011107	49003312	49011107	2	/	*g-ADL*$_{DSF}$*-19-6*	/	/	/	/	⑧
Glyma19g44310	Block_19_49775282_49780336	49775282	49780336	3	/	*g-ADL*$_{DSF}$*-19-7*	/	/	/	/	②/⑦
Glyma19g45170	19_50409190_Block_19_50408788_50410434	50408788	50410434	2	/	/	/	*g-DFM-19-*	/	/	⑩
Glyma19g45260	Block_19_50448994_50455339	50448994	50455339	3	/	/	/	*g-DFM-19-*	/	/	⑥
Glyma20g00565	Block_20_307927_343640	307927	343640	2	/	/	*g-AAT*$_{DSF}$*-20-1*	/	/	/	⑩
Glyma20g01960	Block_20_1475786_1485655	1475786	1485655	3	/	/	/	/	/	*g-AAT*$_{DFM}$*-20-1*	⑩
Glyma20g02500	Block_20_2105970_2116545	2105970	2116545	3	/	/	/	/	*g-ADL*$_{DFM}$*-20-1*	/	⑩
Glyma20g03100	Block_20_2835013_2837565	2835013	2837565	5	/	/	/	*g-DFM-20-1*	*g-ADL*$_{DFM}$*-20-2*	/	⑩
Glyma20g03330	Block_20_3084883_3101046	3084883	3101046	3	/	*g-ADL*$_{DSF}$*-20-1*	/	/	/	/	⑩
Glyma20g04761	20_5050178_Block_20_5050083_5052549	5050083	5052549	2	/	*g-ADL*$_{DSF}$*-20-2*	/	/	/	/	⑤/⑦
Glyma20g08091	Block_20_11238590_11252212	11238590	11252212	4	*g-DSF-20-1*	*g-ADL*$_{DSF}$*-20-3*	/	/	/	/	⑥
Glyma20g18724	Block_20_26281781_26283603	26281781	26283603	4	*g-DSF-20-2*	/	/	/	/	/	⑩
Glyma20g20053	Block_20_28438662_28457888	28438662	28457888	3	/	/	*g-AAT*$_{DSF}$*-20-2*	/	/	/	①/⑤
Glyma20g21151	Block_20_30190512_30193319	30190512	30193319	6	/	*g-ADL*$_{DSF}$*-20-4*	/	/	/	/	⑥
Glyma20g23440	20_33308606_Block_20_33308277_33308965	33308277	33308965	3	/	/	/	/	*g-ADL*$_{DFM}$*-20-3*	/	⑩
Glyma20g23960	Block_20_33681687_33686199	33681687	33686199	2	/	/	/	/	/	*g-AAT*$_{DFM}$*-20-2*	⑩
Glyma20g24830	Block_20_34480819_34487611	34480819	34487611	6	/	/	*g-AAT*$_{DSF}$*-20-3*	/	/	/	⑦
Glyma20g24860	Block_20_34504617_34506172	34504617	34506172	3	/	/	*g-AAT*$_{DSF}$*-20-4*	/	/	/	⑩

Glyma20g25100	Block_20_34802615_34816002	34802615	34816002	*3*	/	/	/	/	*g-ADL$_{DFM}$-20-4*	/	⑥
Glyma20g25790	Block_20_35429047_35431858	35429047	35431858	*4*	*g-DSF-20-3*	/	/	/	/	/	⑨
Glyma20g25800	Block_20_35432475_35451008	35432475	35451008	*4*	/	*g-ADL$_{DSF}$-20-5*	/	/	/	/	⑩
Glyma20g26220	Block_20_35724559_35730480	35724559	35730480	*5*	/	*g-ADL$_{DSF}$-20-6*	/	/	/	/	①/②/⑧
Glyma20g26360	Block_20_35844700_35852990	35844700	35852990	*2*	/	/	*g-AAT$_{DSF}$-20-5*	/	/	/	⑩
Glyma20g26460	Block_20_35932379_35934804	35932379	35934804	*3*	/	/	/	/	*g-ADL$_{DFM}$-20-5*	/	⑩
Glyma20g26640	20_36016966_Block_20_36016916_36018502	36016916	36018502	*2*	/	/	/	*g-DFM-20-2*	/	/	⑩
Glyma20g27700	20_36788337_Block_20_36787573_36792384	36787573	36792384	*2*	/	/	/	*g-DFM-20-3*	/	/	⑥/⑧
Glyma20g27950	20_36941504_Block_20_36940856_36943164	36940856	36943164	*2*	/	/	/	/	*g-ADL$_{DFM}$-20-6*	/	⑧
Glyma20g30150	20_38870210_Block_20_38869243_38872002	38869243	38872002	*2*	/	/	/	/	/	*g-AAT$_{DFM}$-20-3*	⑤
Glyma20g31060	Block_20_39733750_39740377	39733750	39740377	*3*	/	/	/	/	/	*g-AAT$_{DFM}$-20-4*	⑩
Glyma20g31551	20_40162565_Block_20_40161972_40167214	40161972	40167214	*2*	/	*g-ADL$_{DSF}$-20-7*	/	/	/	/	⑤/⑦
Glyma20g32390	Block_20_40994842_41005860	40994842	41005860	*3*	/	*g-ADL$_{DSF}$-20-8*	/	/	*g-ADL$_{DFM}$-20-7*	/	⑨
Glyma20g32540	20_41157466_Block_20_41155444_41158105	41155444	41158105	*2*	/	/	/	/	*g-ADL$_{DFM}$-20-8*	/	⑤
Glyma20g32720	Block_20_41349894_41355859	41349894	41355859	*3*	/	/	/	/	*g-ADL$_{DFM}$-20-9*	/	⑩
Glyma20g32980	Block_20_41596150_41604390	41596150	41604390	*4*	/	*g-ADL$_{DSF}$-20-9*	/	/	/	/	①/⑧
Glyma20g33235	20_41824369_Block_20_41824338_41824778	41824338	41824778	*2*	*g-DSF-20-4*	/	/	/	/	/	⑩
Glyma20g33361	20_41958317_Block_20_41958296_41958857	41958296	41958857	*2*	*g-DSF-20-5*	/	/	/	/	/	⑦
Glyma20g33430	Block_20_42022850_42030461	42022850	42030461	*4*	/	*g-ADL$_{DSF}$-20-10*	/	/	/	/	⑤
Glyma20g34860	Block_20_43160296_43164385	43160296	43164385	*4*	/	/	/	/	/	*g-AAT$_{DFM}$-20-5*	⑥
Glyma20g34910	20_43227587_Block_20_43226352_43228993	43226352	43228993	*2*	/	/	/	*g-DFM-20-4*	/	/	①
Glyma20g35670	Block_20_43892894_43898014	43892894	43898014	*4*	/	/	/	*g-DFM-20-5*	/	/	⑤
Glyma20g36400	20_44526859_Block_20_44524581_44527566	44524581	44527566	*2*	/	/	/	/	/	*g-AAT$_{DFM}$-20-6*	⑩
Glyma20g37960	20_45697660_Block_20_45697077_45700299	45697077	45700299	*2*	*g-DSF-20-6*	/	/	/	/	/	⑧
Glyma20g38090	Block_20_45790561_45791426	45790561	45791426	*3*	/	*g-ADL$_{DSF}$-20-11*	/	/	/	/	⑩
Glyma20g38570	Block_20_46131896_46134314	46131896	46134314	*3*	*g-DSF-20-7*	/	/	/	/	/	②/⑦
Total 661				*1876*	141	130	130	135	124	129	789

Note: DSF: days from sowing-to-flowering; ADL$_{DSF}$: DSF required accumulative day-length; AAT$_{DSF}$: DSF required accumulative active temperature; DFM: days from flowering-to-maturity; ADL$_{DFM}$: DFM required accumulative day-length; AAT$_{DFM}$: DFM required accumulative active temperature.

Gene group: Gene Ontology groups. The four Gene Ontology categories with their groups are as follows: Category I: Genes related to flowering, seed and stem development, or response to light and temperature stimulation, including Group ①, genes related to flower development and growth; Group ②, genes related to light and circadian rhythm; and Group ③, genes related to temperature response. Category II: Translocation signal transduction; defense response; and genes related to DNA methylation, transcription, RNA processing, and chromosome modification, including Group ④, genes related to histone variants and chromosome modification; Group ⑤, genes related to DNA methylation, transcription, and RNA processing; and Group ⑥, genes related to signal transduction and transport. Category III: Primary metabolism genes related to secondary metabolism, including Group ⑦, genes related to plant hormones; Group ⑧, genes related to protein and lipid metabolism; and Group ⑨, genes related to sugar metabolism. Category IV: Genes related to biological processes and unknown functions, including Group ⑩, genes related to other processes or unannotated.

Table S9 Important and active gene–alleles for six DSF- and DFM-related traits in the WSGP.

Gene	Allele code	Allele effect	Gene Type				Gene	Allele code	Allele effect	Gene Type			
			LC	HBC	EM	SH				LC	HBC	EM	SH
Glyma01g22830	$^{\Psi}$**a0**	–123.88	√		√	√	*Glyma17g08460*	§**a0**	20.29	√			√
	$^{\Psi}$**a1**	123.88	√		√	√		§**a1**	–20.29	√			√
Glyma02g04190	§**a0**	–8.90	√		√	√	*Glyma17g09500*	§**a0**	–9.90	√		√	√
	§**a2**	14.66	√		√	√		§**a1**	–8.76	√		√	√
	$^{\Psi}$**a0**	–106.92			√	√		§**a2**	18.67	√		√	√
	$^{\Psi}$**a2**	156.96			√	√		$^{\phi}$**a0**	–177.50	√		√	√
	$^{\phi}$**a0**	–289.27	√		√	√		$^{\phi}$**a2**	312.82	√		√	√
	$^{\phi}$**a1**	–184.67	√		√	√	*Glyma18g08410*	§**a4**	7.78	√			√
	$^{\phi}$**a2**	473.94	√		√	√		$^{\phi}$**a4**	171.11	√			√
Glyma02g06420	§**a2**	–7.46		√	√		*Glyma18g17395*	§**a2**	–7.34	√		√	√

Glyma04g16180	$^{\Psi}$**a0**	−88.59		√	√	
	$^{\Psi}$**a3**	186.07		√	√	
Glyma04g36240	§**a1**	9.54		√	√	√
	§**a2**	−12.41		√	√	√
	$^{\phi}$**a1**	312.65		√	√	√
	$^{\phi}$**a2**	−405.98		√	√	√
Glyma06g23580	§**a2**	12.73	√			√
	§**a3**	−7.08	√			√
	$^{\phi}$**a2**	267.12	√			√
	$^{\phi}$**a3**	−195.23	√			√
Glyma06g36380	$^{\phi}$**a0**	−163.07	√			√
	$^{\phi}$**a1**	163.07	√			√
Glyma08g27633	$^{\Psi}$**a0**	−106.70	√		√	
	$^{\Psi}$**a1**	−113.23	√		√	
	$^{\Psi}$**a3**	239.12	√		√	
Glyma09g28620	$^{\phi}$**a3**	160.36			√	√
Glyma10g26450	§**a4**	6.62	√		√	√
	$^{\phi}$**a0**	−174.83	√		√	√
	$^{\phi}$**a1**	−141.07	√		√	√
	$^{\phi}$**a4**	349.77	√		√	√
Glyma11g14500	§**a1**	−6.46			√	√
	§**a2**	12.39			√	√
	$^{\phi}$**a1**	−334.51	√		√	√
	$^{\phi}$**a2**	458.51	√		√	√
Glyma11g19670	§**a0**	−6.33			√	√
	§**a2**	7.69			√	√
Glyma12g08000	§**a2**	20.83	√		√	√
	§**a3**	−21.69	√		√	√
	$^{\phi}$**a2**	151.62	√		√	√
	$^{\phi}$**a3**	−267.39	√		√	√
Glyma13g07110	§**a2**	6.71	√	√	√	√
	$^{\Psi}$**a0**	−141.00	√	√	√	√
	$^{\Psi}$**a2**	212.08	√	√	√	√
	$^{\phi}$**a2**	228.74	√	√	√	√
Glyma15g34840	$^{\phi}$**a1**	−202.98	√			√
	$^{\phi}$**a2**	239.63	√			√
Glyma17g08460	$^{\Psi}$**a0**	−110.00	√			√
	$^{\Psi}$**a1**	110.00	√			√
	§**a3**	14.95	√		√	√
	$^{\phi}$**a2**	−204.51	√		√	√
	$^{\phi}$**a3**	291.53	√		√	√
Glyma19g03590	$^{\Psi}$**a2**	109.55		√	√	
20	Total 62 (30, 32) [a]		Inherent 35 (23, 12)		Emerged 27 (7, 20)	
Glyma07g09420	$^{¥}$**a4**	9.80			√	√
	$^{£}$**a4**	190.08	√		√	√
Glyma07g10541	$^{£}$**a0**	−117.13			√	√
Glyma10g29970	$^{¥}$**a3**	−7.71			√	√
Glyma12g06620	$^{¥}$**a1**	−9.23		√	√	√
	$^{¥}$**a0**	9.23		√	√	√
	$^{\$}$**a0**	275.51		√	√	√
	$^{\$}$**a1**	−275.51		√	√	√
Glyma13g25480	$^{¥}$**a1**	−9.68	√		√	√
	$^{£}$**a1**	−143.00	√		√	√
	$^{\$}$**a1**	−182.32	√		√	√
	$^{\$}$**a2**	354.21	√		√	√
	$^{¥}$**a4**	8.50	√		√	√
	$^{¥}$**a5**	9.42	√		√	√
Glyma16g07920	$^{£}$**a4**	−101.01		√	√	
Glyma16g25500	$^{\$}$**a0**	−208.33			√	√
	$^{\$}$**a1**	208.33			√	√
Glyma16g33100	$^{£}$**a3**	−142.33			√	√
Glyma17g01160	$^{£}$**a2**	−156.47			√	√
Glyma19g30690	$^{¥}$**a2**	−24.63	√		√	√
	$^{¥}$**a0**	7.69	√		√	√
	$^{¥}$**a1**	16.94	√		√	√
	$^{£}$**a2**	−214.52	√		√	√
	$^{£}$**a0**	101.63	√		√	√
	$^{£}$**a1**	112.88	√		√	√
Glyma19g30750	$^{\$}$**a1**	−482.69	√		√	
	$^{\$}$**a0**	482.69	√		√	
Glyma19g34740	$^{£}$**a0**	−117.59	√		√	√
	$^{£}$**a1**	117.59	√		√	√
	$^{\$}$**a0**	−211.85			√	√
	$^{\$}$**a1**	211.85			√	√
12	Total 31 (16, 15)		Inherent 22 (9, 13)		Emerged 9 (7, 2)	

Note: a0–a7 are the alleles of each gene, arranged in rising order (negative to positive) according to their effect value.
[a]: The number outside parentheses is the number of alleles, and the number inside parentheses is the number of negative and positive alleles; §: represents the allelic change in DSF; $^{\Psi}$: represents the allelic change in ADL_{DSF}; $^{\phi}$: represents the allelic change in AAT_{DSF}; $^{¥}$: represents the allelic change in DFM; $^{£}$: represents the allelic change in ADL_{DFM}; $^{\$}$: represents the allelic change in AAT_{DFM}. LC: represents the gene with $R^2 \geq 1.5\%$. HBC: genes with the top 10% of BC scores in PPI network genes; here, BC is betweenness centrality, a centrality measurement and assignment. EM: genes containing alleles that only appear in subpopulations "A", "B", "C", and "D" compared with subpopulation "O". SH: genes shared by DSF or DFM and required accumulative day-length and accumulative effective temperature.
